# Molecular Phylogenetics and Micromorphology of Australasian Stipeae (Poaceae, Subfamily Pooideae), and the Interrelation of Whole-Genome Duplication and Evolutionary Radiations in This Grass Tribe

**DOI:** 10.3389/fpls.2020.630788

**Published:** 2021-01-22

**Authors:** Natalia Tkach, Marcin Nobis, Julia Schneider, Hannes Becher, Grit Winterfeld, Surrey W. L. Jacobs, Martin Röser

**Affiliations:** ^1^Department of Systematic Botany, Institute of Biology, Geobotany and Botanical Garden, Martin Luther University Halle-Wittenberg, Halle (Salle), Germany; ^2^Institute of Botany, Faculty of Biology, Jagiellonian University, Kraków, Poland; ^3^Institute of Evolutionary Biology, School of Biological Sciences, University of Edinburgh, Edinburgh, United Kingdom; ^4^National Herbarium, Royal Botanic Gardens, Sydney, NSW, Australia

**Keywords:** *Anemanthele*, *Austrostipa*, chromosome number, dysploidy, lemma micromorphology, phylogeny, taxonomy, whole-genome duplication

## Abstract

The mainly Australian grass genus *Austrostipa* (tribe Stipeae) comprising approximately 64 species represents a remarkable example of an evolutionary radiation. To investigate aspects of diversification, macro- and micromorphological variation in this genus, we conducted molecular phylogenetic and scanning electron microscopy (SEM) analyses including representatives from most of *Austrostipa*’s currently accepted subgenera. Because of its taxonomic significance in Stipeae, we studied the lemma epidermal pattern (LEP) in 34 representatives of *Austrostipa.* Plastid DNA variation within *Austrostipa* was low and only few lineages were resolved. Nuclear ITS and *Acc1* yielded comparable groupings of taxa and resolved subgenera *Arbuscula*, *Petaurista*, and *Bambusina* in a common clade and as monophyletic. In most of the *Austrostipa* species studied, the LEP was relatively uniform (typical maize-like), but six species had a modified cellular structure. The species representing subgenera *Lobatae*, *Petaurista*, *Bambusina* as well as *A. muelleri* from subg. *Tuberculatae* were well-separated from all the other species included in the analysis. We suggest recognizing nine subgenera in *Austrostipa* (with number of species): *Arbuscula* (4), *Aulax* (2), *Austrostipa* (36), *Bambusina* (2), *Falcatae* (10), *Lobatae* (5), *Longiaristatae* (2), *Petaurista* (2) and the new subgenus *Paucispiculatae* (1) encompassing *A. muelleri*. Two paralogous sequence copies of *Acc1*, forming two distinct clades, were found in polyploid *Austrostipa* and *Anemanthele*. We found analogous patterns for our samples of *Stipa* s.str. with their *Acc1* clades strongly separated from those of *Austrostipa* and *Anemanthele*. This underlines a previous hypothesis of Tzvelev (1977) that most extant Stipeae are of hybrid origin. We also prepared an up-to-date survey and reviewed the chromosome number variation for our molecularly studied taxa and the whole tribe Stipeae. The chromosome base number patterns as well as dysploidy and whole-genome duplication events were interpreted in a phylogenetic framework. The rather coherent picture of chromosome number variation underlines the enormous phylogenetic and evolutionary significance of this frequently ignored character.

## Introduction

Feathergrasses (tribe Stipeae) have long attracted the interest of scientists not only because of their enormous morphological variation but also because of their worldwide ecological significance as important constituents of grasslands (steppes, prairies) under mesic to xeric, sometimes in rather cold climates, where they are often an important source of food for livestock (e.g., Kellogg, 2015). The delineation of this tribe, its taxonomic structure with regards to major lineages and the circumscription of its genera has been investigated during the past three decades using morphological, anatomical and, more recently, molecular phylogenetic approaches (Freitag, 1975, 1985; Tzvelev, 1976; Barkworth, 1983, 1990, 1993, 2007; Vickery et al., 1986; Barkworth and Everett, 1987; Jacobs and Everett, 1996; Edgar and Connor, 2000; Jacobs et al., 2000, 2007; Barkworth and Torres, 2001; Peñailillo, 2002, 2003; Soreng et al., 2003; Wu and Phillips, 2006; Cialdella et al., 2007, 2010, 2014; Barkworth et al., 2008; Romaschenko et al., 2008, 2010, 2011, 2012, 2014; Barber et al., 2009; Everett et al., 2009; Schneider et al., 2009, 2011; Vázquez Pardo and Gutiérrez Esteban, 2011; Hamasha et al., 2012; Sclovich et al., 2015; Winterfeld et al., 2015; Krawczyk et al., 2017, 2018; Nobis et al., 2019a,b, 2020; Peterson et al., 2019).

The tribe Stipeae includes approximately 530 species in 33 genera and has almost worldwide distribution (Peterson et al., 2019). It has major centers of radiation in Eurasia (especially in *Stipa* L. s.str., *Piptatherum* P.Beauv., *Achnatherum* P.Beauv.) and the Americas [in *Eriocoma* Nutt., *Nassella* (Trin.) É.Desv., *Jarava* Ruiz & Pav., *Pappostipa* (Speg.) Romasch., P.M.Peterson & Soreng, *Piptochaetium* J.Presl]. *Austrostipa* S.W.L.Jacobs & J.Everett and the endemic New Zealand genus *Anemanthele* Veldkamp (one species) are together with New Zealand *Achnatherum petriei* (Buchanan) S.W.L.Jacobs & J.Everett the only indigenous representatives of this tribe in the Australian Plant Kingdom.

*Austrostipa* is the third largest genus in the Stipeae and encompasses approximately 64 species, most of which occur in Australia and Tasmania (Vickery et al., 1986; Jacobs and Everett, 1996; Everett et al., 2009; Williams, 2011). Only one species, *A. stipoides* (Hook.f.) S.W.L.Jacobs & J.Everett, is considered native to New Zealand; it is also present in southeastern Australia. Other species of Australian origin are naturalized in New Zealand (Jacobs et al., 1989; Edgar and Connor, 2000). One species, *A. scabra* (Lindl.) S.W.L.Jacobs & J.Everett, is present on Easter Island; it is thought to have been introduced there after 1860 (Everett and Jacobs, 1990). Ecologically, *Austrostipa* is adapted to the warm- to hot-summer Mediterranean climate of SW, S and SE Australia and the more Oceanic climate of SE Australia and Tasmania. This rather isolated southern outpost of Stipeae is widely separated from temperate Eurasia and the Americas, both regions with high diversity in Stipeae genera. *Austrostipa* displays a tremendous morphological diversity in, for example, habit, growth form, the size and form of individual structures such as spikelets, glumes, etc. Moreover, the rich evolutionary diversification developed sympatrically, primarily in a comparatively narrow coastal strip of S Australia. This region has Mediterranean-type to steppe-like climate with open vegetation, similar to characterizing other areas of stipoids diversity. Many *Austrostipa* species seem to be edaphically specialized, being restricted to specific soil types (Everett et al., 2009; Williams, 2011).

Characters of the lemma, visible even under low magnification, are taxonomically important in Poaceae and frequently used in species identification. The taxonomic value of micromorphological characters of the lemma epidermis is also substantial in many genera of grasses (for example, Thomasson, 1978, 1981, 1986; Terrell and Wergin, 1981; Barkworth and Everett, 1987; Valdés-Reyna and Hatch, 1991; Snow, 1996; Acedo and Llamas, 2001; Terrell et al., 2001; Mejía Saulés and Bisby, 2003; Ortúñez and de la Fuente, 2010; Nobis, 2013). Thomasson (1978, 1981) was the first to use lemma epidermal characters in the Stipeae, demonstrating the value of such features as the presence of hooks, the shape of the long cells and the presence of silica cells in elucidating the phylogeny of the tribe. More recently, Romaschenko et al. (2010, 2012) have described two major lemma epidermal patterns in the tribe: *Stipa*-like, also called saw-like, dominated by long fundamental cells and hooks, and *Achnatherum*-like, also called maize-like, dominated by short fundamental cells and paired with silica cells. Several authors have shown out that, even though LEP is relatively uniform within a genus, it may still be useful in identifying particular species as well as in delineating relationships among and between different subgenera or sections (Ortúñez and de la Fuente, 2010; Nobis, 2013; Olonova et al., 2016; Nobis et al., 2019b), but lemmas of relatively few *Austrostipa* species had been studied prior to Bustam’s (2010; 2012) work.

Most research (Barkworth et al., 2008; Hamasha et al., 2012; Romaschenko et al., 2012) supports Jacobs and Everett (1996) in recognizing *Austrostipa* as separate from, and only distantly related to, *Stipa* s.str. Morphologically, *Austrostipa* has several floret characteristics (e.g., long, sharp calluses, lemmas are often dark and have tough margins, glabrous and prow-tipped paleas) that, although not individually unique to the genus, in combination distinguish it from other genera, including the rather similar and poorly understood genus *Achnatherum* (Jacobs and Everett, 1996). The closest extant relatives of *Austrostipa* within the Stipeae, however, have not yet been unequivocally identified. Analyses of morphological and anatomical data placed *Austrostipa* in a clade together with *Achnatherum* and *Ptilagrostis* Griseb. (Jacobs and Everett, 1996; Jacobs et al., 2000, Figure 2). Previous molecular phylogenetic studies showed *Austrostipa* forming a clade together with the main part of *Achnatherum*, the American genera *Nassella*, *Jarava*, and several smaller genera (for example, *Amelichloa* Arriaga & Barkworth, *Celtica* F.M.Vázquez & Barkworth, *Stipellula* Röser & Hamasha), which represented one of the well supported major lineages within the tribe (Barkworth et al., 2008; Romaschenko et al., 2008, 2010, 2012; Cialdella et al., 2010; Hamasha et al., 2012). Most studies sampled only one or a few species of *Austrostipa*, making it hard to assess the monophyly of this genus. One species of *Austrostipa* was sampled for the internal transcribed spacer (ITS) regions of nrDNA by Hsiao et al. (1999), 13 for ITS and five plastid DNA regions by Romaschenko et al. (2008, 2010, 2012), six for ITS1 and seven for four plastid DNA regions by Barkworth et al. (2008), two for four plastid DNA regions by Cialdella et al. (2010), five for ITS and two for one plastid DNA region by Hamasha et al. (2012) as well as 25 for ITS and one plastid DNA region by Winterfeld et al. (2015). The ITS studies of Jacobs et al. (2000, 2007) encompassed 15 and 37 species, respectively. While monophyly of *Austrostipa* was supported by the former study, sequences of some species of *Achnatherum*, *Nassella*, and *Stipa* were interspersed in the *Austrostipa* clade of the latter. In both studies, the New Zealand endemic *Anemanthele* was included in the *Austrostipa* clade, but its position was unstable. The most comprehensive study conducted so far included 31 taxa for ITS and 52 for two plastid DNA regions (Syme et al., 2012).

Overall variation between individual *Austrostipa* ITS sequences was low and the differences between sequences from different accessions of the same species was often not much smaller than between sequences of different species (Jacobs et al., 2007). This overall low variation made it difficult to compare their results with classification of *Austrostipa* into 13 subgenera (Table 1; Jacobs and Everett, 1996; Everett et al., 2009). The main characters employed were growth form, branching of the culms, characters of the spikelets (glumes, lemmas, awns, paleas) and the formation of dispersal units (whole panicle or florets). Some of the subgenera were reflected in the ITS data (for example, subg. *Falcatae* S.W.L.Jacobs & J.Everett), whereas others were mixed up (for example, subg. *Austrostipa* and subg. *Tuberculatae* S.W.L.Jacobs & J.Everett or subg. *Arbuscula* S.W.L.Jacobs & J.Everett and subg. *Bambusina* S.W.L.Jacobs & J.Everett, respectively), or were entirely unresolved (Jacobs et al., 2007; Syme et al., 2012). The plastid DNA analyses resolved two main clades, neither of which corresponded to the recognized subgenera, and further resolution was low (Syme et al., 2012). By using a combination of morphological and molecular approaches, this study addresses the main phylogenetic and evolutionary problems regarding *Austrostipa*, namely its monophyly and its internal phylogenetic structure. These questions are treated using on a broader sample of *Austrostipa* taxa by generating a taxonomically overlapping set of nr ITS and plastid DNA sequences of the 3′*trnK* region. Both molecular markers are frequently utilized and well-established in molecular phylogenetic studies (Baldwin et al., 1995; Liang and Hilu, 1996), although ITS from the repetitive 18S–26S nrDNA can be polymorphic in individual genomes for several reasons. This may lead to paralogous sequence relationships that can potentially confound phylogenetic reconstruction (Buckler et al., 1997; Álvarez and Wendel, 2003; Bailey et al., 2003; Razafimandimbison et al., 2004; Bayly and Ladiges, 2007; Nieto Feliner and Rosselló, 2007; Schneider et al., 2009, 2011). Nonetheless, ITS is a nuclear marker useful to investigate. As a second nuclear marker we studied the single-copy gene *Acc1* encoding plastid acetyl-CoA carboxylase 1 (Huang et al., 2002; Fan et al., 2007, 2009; Sha et al., 2010; Hochbach et al., 2015). The 3′*trnK* region, comprising the 3′part of the chloroplast *matK* gene with following intron and 3′*trnK* exon, was selected as sequence marker from the plastid DNA mainly because of its comparatively high substitution rate. Moreover, these sequences are straightforward to align and are already available in many potential outgroup taxa from within Stipeae and neighboring tribes (Döring et al., 2007; Schneider et al., 2009, 2011, 2012; Hamasha et al., 2012; Blaner et al., 2014; Wölk and Röser, 2014, 2017; Hochbach et al., 2015, 2018; Tkach et al., 2020).

**TABLE 1:** Overview of sampling density among the 13 previous subgenera of *Austrostipa* used for molecular phylogenetic analyses.

Subgenus	3′*trnK*	ITS	*Acc1*
*Arbuscula* (4)	3	3	2
*Aulax* (2)	1	2	1
*Austrostipa* (7)	6	6	5
*Bambusina* (2)	2	2	2
*Ceres* (6)	5	5	1
*Eremophilae* (6)	2	3	1
*Falcatae* (10)	7	9	2
*Lancea* (7)	4	4	2
*Lanterna* (3)	1	1	1
*Lobatae* (6)	4	4	2
*Longiaristatae* (2)	1	2	1
*Petaurista* (2)	2	2	1
*Tuberculatae* (7)	5	5	1

*The number of species sampled relative to the total number of species (in brackets) according to Jacobs and Everett (1996), Everett et al. (2009), and Williams (2011) is shown for the three DNA regions analyzed.*

The sequence data from the nuclear and the plastid genome are used to examine the potential role of hybridization, reticulation and the origin of polyploidy in *Austrostipa*. The results of the phylogenetic analyses were further used to discuss the cytogenetic characteristics of this genus and other stipoids. To this end, we conducted an up-to-date survey of chromosome numbers in the Stipeae and discussed the chromosome base number(s), dysploid variation and the evolutionary role of whole-genome duplications in this tribe.

## Materials and Methods

### Plant Material

The sample for the molecular phylogenetic study included 51 species and subspecies of *Austrostipa*. Geographic origin, collector or seed exchange locality and herbarium vouchers for the taxa used in this study are listed in Supplementary Appendix 1. For half of the species more than one specimen was included. Sampling density among the 13 subgenera for the analyzed DNA regions 3′*trnK* (3′part of the chloroplast *matK* gene with the following intron and 3′*trnK* exon), ITS and *Acc*1 is summarized in Table 1. The dataset of the 3′*trnK* region encompassed 43, that of ITS 48 *Austrostipa* species, representing 71 and 75% of the total species number, respectively. All subgenera except for subg. *Lanterna* S.W.L.Jacobs & J.Everett were represented by at least half their species. For the analysis of the nuclear single-copy gene *Acc*1 sequence data we selected at least one specimen of each subgenus and studied a total of 22 (33%) species. In addition, we included representatives of eight other stipoid genera [*Achnatherum*, *Anemanthele*, *Celtica*, *Nassella*, *Neotrinia* (Tzvelev) M.Nobis, P.D.Gudkova & A.Nowak, *Oloptum* Röser & Hamasha, *Stipa*, *Stipellula*] that previous studies have shown to be most closely related to *Austrostipa* (Jacobs et al., 2000, 2007; Barkworth et al., 2008; Romaschenko et al., 2008, 2010, 2012; Hamasha et al., 2012). Genera from the tribes Bromeae (*Bromus* L.), Duthieeae (*Anisopogon* R.Br.) and Triticeae (*Henrardia* C.E.Hubb., *Hordeum*, *Secale* L.) were chosen as outgroups for phylogenetic reconstructions based on studies of phylogenetic relationships within subf. Pooideae (for example, Catalán et al., 1997; Hilu et al., 1999; Mathews et al., 2000; Soreng and Davis, 2000; GPWG (Grass Phylogeny Working Group), 2001; Davis and Soreng, 2007; Döring et al., 2007; Soreng et al., 2007; Schneider et al., 2009, 2011; Saarela et al., 2015, 2018). For the *Acc*1 dataset, data for selected outgroup species of Bromeae (*Bromus inermis* Leyss.) and Triticeae (*Henrardia persica* (Boiss.) C.E.Hubb., *Hordeum chilense* Roem. & Schult., *H. vulgare* L.) as well as some 3′*trnK* and ITS sequences were taken from ENA/GenBank (Supplementary Appendix 1).

Most plant material used in this study for DNA extraction was collected in the field in 2007 by SWLJ and Mary E. Barkworth (Logan, UT, United States) along with herbarium specimens and duplicates, which have been subsequently distributed to various herbaria (Supplementary Appendix 1). The leaf samples were preserved in saturated NaCl/CTAB buffer solution prepared according to Štorchová et al. (2000). Further leaf material for DNA extraction was collected from living pot plants grown from seeds stored at the Millennium Seed Bank (Wakehurst Place, Royal Botanic Gardens, Kew, United Kingdom). These caryopses were collected from natural populations with verified identifications and voucher specimens deposited at K and, in some instances, at PERTH (Supplementary Appendix 1). The pot plants were cultivated in the greenhouses of the Botanical Garden of the University Halle-Wittenberg (vouchers at HAL). Leaves for DNA extractions were silica gel-dried (Chase and Hills, 1991). These living plants were also used for cytogenetic studies by Winterfeld et al. (2015).

### DNA Extraction, PCR Amplification and Sequencing

For DNA extraction, leaves preserved in NaCl/CTAB buffer were removed from the solution, rinsed in water, immersed in liquid nitrogen, and then ground to fine powder using mortar and pestle. Silica gel-dried fresh leaves were shredded in a FastPrep FP 120 bead mill homogenizer (Qbiogene, Heidelberg, Germany). The ready-to-use NucleoSpin Plant Kit (Macherey-Nagel, Düren, Germany) was used for extraction.

**FIGURE 1:**
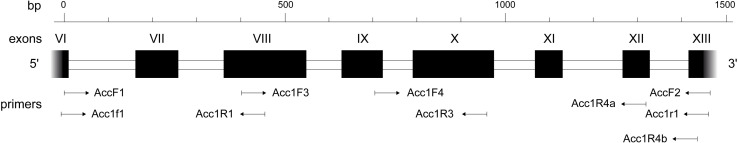
Schematic of the acetyl-CoA carboxylase gene (*Acc1*) modified from Huang et al. (2002) with designations, locations, and directions of the primers used.

The ITS and 3′*trnK* region were amplified and sequenced as in our previous studies with primers listed in Table 2 (Schneider et al., 2009, 2011, 2012; Hamasha et al., 2012; Winterfeld et al., 2015). The amplification of *Acc*1 (exons 6–13 and intervening introns) was carried out using primers also listed in Table 2. An overview of the gene *Acc1* is shown in Figure 1 together with the locations, directions and designations of the primers used in this study.

**TABLE 2:** Primers used to amplify and sequence the plastid 3′*trnK* region, nuclear ITS1–5.8S gene–ITS2 and the *Acc1* gene (exons 6-13 and intervening introns).

DNA region and primer name	5′–Primer sequence–3′	References
**3′*trnK* region**		
PO-matK 1300F	TCAGATTGGGATATTCTTGATCG	Döring et al., 2007; Schneider et al., 2009
psbA-R	CGCGTCTCTCTAAAATTGGAGTCAT	Johnson and Soltis, 1994
**ITS1–5.8S gene–ITS2**		
ITS-A	GGAAGGAGAAGTCGTAACAAGG	Blattner, 1999
ITS-B	CTTTTCCTCCGCTTATTGATATG	Blattner, 1999
ITS-C	GCAATTCACACCAAGTATCGC	Blattner, 1999
ITS-D	CTCTCGGCAACGGATATCTCG	Blattner, 1999
***Acc1***		
AccF1	CCCAATATTTATCATGAGACTTGCA	Huang et al., 2002; Fan et al., 2009; Sha et al., 2010
AccF2	CAACATTTGAATGAATHCTCCACG	Huang et al., 2002; Fan et al., 2009; Sha et al., 2010
Acc1f1	GTTCCTGGCTCCCCAATATTTATC	Huang et al., 2002; Hand et al., 2010
Acc1r1	TTCAAGAGATCAACTGTGTAATCA	Huang et al., 2002; Hand et al., 2010
Acc1F3	ATTGAGGAAGGRCCAGWTACTG	This study
Acc1F4	GTTGCAGTTGGAATGGGTAT	This study
Acc1R1	CCACAGCCTTAGCAAGCCTCC	This study
Acc1R3	GTTATCCTAACTGCTACACAA	This study
Acc1R4a	CAAAACTGAGAATCAGCA	This study
Acc1R4b	TTGGTTATTGCWGCTGATCTAG	This study

*For *Acc1* primer positions see Figure 1.*

**FIGURE 2:**
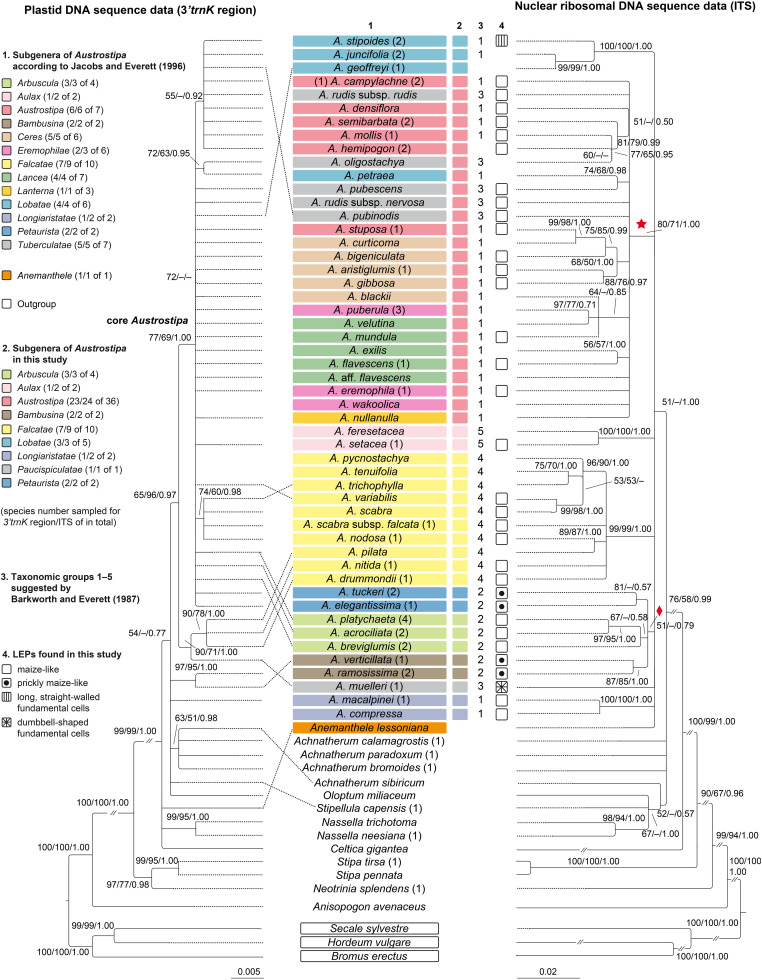
Maximum likelihood phylograms of *Austrostipa* species, *Anemanthele lessoniana* and exemplary other taxa of tribe Stipeae inferred from plastid (3′*trnK* region) and nuclear ribosomal (ITS1–5.8S gene–ITS2) DNA sequences with *Bromus erectus* (Bromeae), *Hordeum vulgare*, *Secale sylvestre* (both Triticeae), and *Anisopogon avenaceus* (Duthieeae) used as outgroup. Reduced datasets with each taxon represented by a single representative accession. ML and MP bootstrap support values ≥ 50% as well as Bayesian PP ≥ 0.5 are indicated on the branches. Clades with ML support < 50% are collapsed. The asterisked clade in the ITS tree was recovered also in the *Acc1* gene tree of Figure 3 (copy types A and B) as well as the clade with diamond (copy type A). The taxonomic groupings of the *Austrostipa* species according to different treatments are marked by different colors or numbers in columns 1–3. The LEPs studied are represented in column 4. Numbers in brackets after taxon names refer to individual accessions as listed in the Supplementary Appendix 1. *A*., *Austrostipa*.

For all studied DNA regions, the PCR reactions of 20 μl usually contained 0.5 μM of each primer, 2 μl of 10 × PCR buffer, 1.9 mM MgCl_2_, 0.8–1 U *Taq* DNA polymerase (all MP Biomedicals, Heidelberg, Germany), 5% DMSO (AppliChem, Darmstadt, Germany), 100 μM dNTPs (GeneCraft, Lüdinghausen, Germany), 1–2 μl of template DNA (∼50 ng) and distilled water.

For DNA samples, which were obtained by extraction from leaves preserved in saturated NaCl/CTAB buffer solution, the PCR reaction was performed with 3 min at 94°C, followed by 35 cycles of 30 s at 94°C, 30 s–2 min at 50°C, 5 min at 68°C, and a final extension for 20 min at 68°C.

The DNA extracted from silica gel-dried leaf material was amplified by the following PCR program: 3 min at 94°C, followed by 35 cycles of 30 s at 94°C, 30 s–2 min at 50°C, 2 min at 72°C, and a final extension for 20 min at 72°C. PCR products of *Acc*1 were column-purified with the NucleoSpin Extract II Kit (Macherey-Nagel).

Due to the presence of different *Acc1* copies in *Austrostipa* species, *Anemanthele lessoniana* (Steud.) Veldkamp and other polyploids (Supplementary Appendix 2; Winterfeld et al., 2015), *Acc1* amplicons were cloned into the pGEM-T Easy Vector (Promega, Mannheim, Germany) according to the manufacturer’s protocol. In the next step 10–30 individual white colonies containing the insert were picked. The isolation of plasmid DNA was performed with the Wizard Plus SV Minipreps DNA Purification System (Promega). The insert of the purified plasmid DNA was sequenced using the standard primers T7 and SP6. The sequencing was performed by StarSEQ GmbH (Mainz, Germany) or Eurofins MWG Operon (Ebersberg, Germany).

### Alignment and Phylogenetic Analysis

All sequences were edited by eye in Sequencher v.5.0 (Gene Codes, Ann Arbor, MI, United States). The automatically performed alignments by using ClustalW2 (Larkin et al., 2007) were manually adjusted in Geneious v.9.1.6^1^ (Kearse et al., 2012). We identified few double peaks in chromatograms of the ITS dataset already documented in our previous study (Winterfeld et al., 2015). It was possible to edit these single nucleotide positions by IUPAC code and include all obtained ITS sequences.

All clone-derived sequences of the *Acc1* dataset were visually checked for the presence of chimerical sequences or PCR artifacts (see Brassac et al., 2012). Furthermore, we tested the protein sequence of the exon regions (696 bp) for each clone and compared the translation to the *Acc1* sequence of the diploid outgroup, *Bromus inermis*, taken from ENA/GenBank (Supplementary Appendix 1). We excluded chimerical sequences and clones different from that of *Bromus inermis* in more than 20 amino acid positions of the exon regions. To reduce the number of singletons in the alignment, we summarized for each specimen highly similar *Acc1* sequences of the remaining individual clones to consensus sequences.

Sequences of ITS and 3′*trnK* region as well as the individual *Acc*1 clones used for assembling consensus sequences were submitted to ENA/GenBank under the accession numbers LR989057–LR989267 (Supplementary Appendix 1).

All DNA sequence datasets were analyzed using the phylogenetic approaches of maximum likelihood (ML), maximum parsimony (MP), and Bayesian inference (BI) following Tkach et al. (2019, 2020). The trees were visualized with FigTree v.1.4.3^2^. Support values are cited in the text in the following sequence: ML bootstrap support/MP bootstrap support/Bayesian posterior probability (PP).

### Morphological Analyses

We scored 65 species and subspecies of *Austrostipa* for nine morphological characters commonly used in identification keys in 1–5 specimens each or gathered the information from morphological descriptions of the taxa (Everett et al., 2009). The characters studied were: mean length of ligules of the culm leaves; surface of inflorescence branches (glabrous, with prickles or macrohairs); mean length of glumes, calli, lemmas, lemma lobes and awns; mean length ratio lemma:palea; shape of palea apex (without or with 2–4 teeth) (Table 3, Supplementary Table 1, and Supplementary Appendix 1). These characters were chosen to evaluate morphological groupings of *Austrostipa* taxa proposed by Jacobs and Everett (1996) and Everett et al. (2009).

**TABLE 3:** Morphological characters and character states.

	Character states
**Macromorphological characters**	
Ligules	Mean length [mm]
Glumes	Mean length [mm]
Callus	Mean length [mm]
Lemma	Mean length [mm]
Lemma lobes	Mean length [mm]
Awn	Mean length [mm]
Length lemma:palea	Ratio
Inflorescence branches	Glabrous or scabrous (0); pilose (1)
Palea apex	2–4-toothed (0); without teeth (1)
**Micromorphological characters of the lemma epidermis**	
Fundamental cells	Wider than long to as long as wide (1); up to 1.8 times longer than wide (2); 2–3 times longer than wide (3)
Hooks	Present (1); absent (2)
Silica cells	Elongated, 2–3 times longer than wide (1); ovate to elliptic, up to 1.5 times longer than wide (2)
Cork cells	Present (1); absent (2)

### Lemma Micromorphology

The ultrastructure of the lemma epidermis was studied in 34 taxa (species and subspecies) of *Austrostipa* (Supplementary Appendix 1). For scanning electron microscopy (SEM), dry samples were coated with a thin layer of gold using a JFC-1100E ion sputter (JEOL), then observed and photographed on a Hitachi S-4700 scanning electron microscope. Four diagnostic micromorphological characters, namely fundamental cells, silica cells, cork cells and hooks were recorded. We examined the middle part of the abaxial lemma surface as being the least variable. It differs from the upper part, in which a variable admixture of hooks, prickles and macrohairs is usually observed.

### Numerical Analyses

The numerical analyses were performed on the same 34-taxa set based on (1) four above-mentioned micromorphological characters, and (2) a combination of four micromorphological with eight macromorphological characters (Table 3 and Supplementary Table 2). Each taxon was treated as an Operational Taxonomic Unit (OTU), in accordance with the methods used in numerical taxonomy (Sokal and Sneath, 1963). The similarities among OTUs were calculated using Gower’s general similarity coefficient. Cluster analysis, using PAST software (Hammer et al., 2001), was performed on all OTUs to estimate morphological similarities among the species.

### Chromosome Numbers in Stipeae

To address the significance of the cytogenetic data in *Austrostipa* and relatives in a phylogenetic context of Stipeae, we extensively surveyed the published chromosome numbers. We prepared a comprehensive up-to-date list of chromosome numbers of Stipeae taxa (164 species, 22 infraspecific taxa) with currently accepted taxon names and painstakingly regarded nomenclature and synonyms used in the original publications (Supplementary Appendix 2). Because the secondary literature frequently had reported incorrect numbers or wrongly cited the actual authors of the chromosome counts, we checked more than 150 original references. A few original publications we could not examine are identified as such in the references list of Supplementary Appendix 2.

To infer the evolutionary history of chromosome and genomic characters of special interest utilizing a simple cladistics analysis, we mapped chromosome base numbers, occurrence of dysploidy and whole-genome duplications in the evolution of tribe Stipeae on a molecular phylogenetic cladogram simplified and modified from the plastid DNA tree of Romaschenko et al. (2012) and the plastid/nuclear DNA tree (concatenated data of congruent taxa) of Hamasha et al. (2012). The treatment of genera and estimated number of species largely followed Peterson et al. (2019).

## Results

### Molecular Phylogenetics

We analyzed a dataset of 110 DNA sequences for the 3′*trnK* region and 111 for ITS, respectively. The *Acc1* dataset comprised a total of 266 clone-derived sequences. After evaluation of all clones of the polyploid genera *Austrostipa* and *Anemanthele*, we created 61 consensus sequences for the final dataset. We obtained two or three distinct *Acc1* consensus sequences for each *Austrostipa* species with the exception of *A. breviglumis* (J.M.Black) S.W.L.Jacobs & J.Everett, which had only one consensus sequence. For tetraploid *Stipa capillata* L. and *S. tirsa* Steven (both 2*n* = 44; Supplementary Appendix 2) we identified two different *Acc1* copy types after analyzing the clone sequences. For diploid *Achnatherum paradoxum* (L.) Banfi, Galasso & Bartolucci and *A. sibiricum* (L.) Keng ex Tzvelev (both 2*n* = 24) as well as polyploid *Nassella trichotoma* (Nees) Hack. & Arechav. (2*n* = 36, 38; Supplementary Appendix 2), only one sequence with clear peaks in the chromatograms was identified from direct sequencing of the PCR products.

The topology of the trees inferred by ML, MP, and BI analyses were largely identical although their statistical supports differed slightly. Figure 2 shows trees with plastid and nuclear ITS DNA data reduced to a single accession per taxon. The complete phylograms with all studied accessions are presented in Supplementary Figures 1, 2.

#### Plastid DNA Analysis – 3′*trnK* Region

The plastid 3′*trnK* region DNA sequence dataset (sequence lengths 579*–*798 bp) for 63 taxa of the reduced dataset (each species or subspecies represented by only one accession) included 832 aligned positions, of which 139 were variable (17%) and 59 parsimony-informative (7.0%).

*Austrostipa* is characterized by the occurrence of at least three different plastid DNA types (Figure 2). After branching of the outgroup taxa (*Bromus erectus* Huds., *Hordeum vulgare*, *Secale sylvestre* Host, and *Anisopogon avenaceus* R.Br. next to the stipoid taxa), a clade formed by *Neotrinia splendens* (Trin.) M.Nobis, P.D.Gudkova & A.Nowak, *Stipa pennata* L. and *S. tirsa* was sister to all other Stipeae sampled (100/100/1.00) (Figure 2). *Anemanthele lessoniana*, *Celtica gigantea* (Link) F.M.Vázquez & Barkworth, *Nassella neesiana* (Trin. & Rupr.) Barkworth, and *N. trichotoma* stood in a polytomy with *Oloptum miliaceum* (L.) Röser & Hamasha, *Stipellula capensis* (Thunb.) Röser & Hamasha, a clade of four *Achnatherum* species [*A. bromoides* (L.) P.Beauv., *A. calamagrostis* (L.) P.Beauv., *A. paradoxum*, *A. sibiricum*], three *Austrostipa* species [*A. macalpinei* (Reader) S.W.L.Jacobs & J.Everett, *A. ramosissima* (Trin.) S.W.L.Jacobs & J.Everett, *A. verticillata* (Nees ex Spreng.) S.W.L.Jacobs & J.Everett] and the remainder of the latter genus (Figure 2).

*Austrostipa drummondii* (Steud.) S.W.L.Jacobs & J.Everett, *A. muelleri* (Tate) S.W.L.Jacobs & J.Everett, *A. nitida* (Summerh. & C.E.Hubb.) S.W.L.Jacobs & J.Everett and *A. pilata* (S.W.L.Jacobs & J.Everett) S.W.L.Jacobs & J.Everett formed a supported clade (97/95/1.00), which was sister to a large polytomy of all other species studied (77/69/1.00), here termed ’core *Austrostipa*’ clade. Among them, *A. oligostachya* (Hughes) S.W.L.Jacobs & J.Everett and *A. petraea* (Vickery) S.W.L.Jacobs & J.Everett formed a moderately supported species pair (72/63/0.95). Groups of varying size and mostly low support were formed by (1) *A. nodosa* (S.T.Blake) S.W.L.Jacobs & J.Everett, *A. scabra*, *A. scabra* subsp. *falcata* (Hughes) S.W.L.Jacobs & J.Everett, *A. trichophylla* (Benth.) S.W.L.Jacobs & J.Everett (74/60/0.98), and (2) *A. campylachne* (Nees) S.W.L.Jacobs & J.Everett, *A. densiflora* (Hughes) S.W.L.Jacobs & J.Everett, *A. hemipogon* (Benth.) S.W.L.Jacobs & J.Everett, *A. juncifolia* (Hughes) S.W.L.Jacobs & J.Everett, *A. mollis* (R.Br.) S.W.L.Jacobs & J.Everett, *A. pubinodis* (Trin. & Rupr.) S.W.L.Jacobs & J.Everett, *A. rudis* (Spreng.) S.W.L.Jacobs & J.Everett subsp. *rudis*, *A. semibarbata* (R.Br.) S.W.L.Jacobs & J.Everett and *A. stipoides* (55/−/0.92) (Figure 2).

#### Nuclear DNA – ITS

The reduced nr ITS DNA sequence dataset for 68 taxa (each species or subspecies represented by only one accession) included 644 aligned positions (sequence lengths 500*–*627 bp), of which 263 (41%) were variable and 185 (29%) parsimony-informative.

Following the outgroup taxa (*Bromus erectus*, *Hordeum vulgare*, *Secale sylvestre*, and *Anisopogon avenaceus* next to the stipoid taxa), representatives from several Stipeae genera including *Achnatherum*, *Celtica*, *Nassella*, *Neotrinia*, *Oloptum*, *Stipa*, and *Stipellula* were next to a clade of *Anemanthele* and *Austrostipa* (51/−/1.00) (Figure 2). Overall resolution within this clade was low, however, several supported groups of species or species pairs could be discerned, for example, that of (1) *A. compressa*, *A. macalpinei* (100/100/1.00), (2) *A. ramosissima*, *A. verticillata* (87/85/1.00), (3) *A. acrociliata* (Reader) S.W.L.Jacobs & J.Everett, *A. breviglumis*, *A. platychaeta* (Hughes) S.W.L.Jacobs & J.Everett (67/−/0.58), (4) *A. elegantissima* (Labill.) S.W.L.Jacobs & J.Everett, *A. tuckeri* (F.Muell.) S.W.L.Jacobs & J.Everett (87/−/0.57), (5) a larger clade encompassing *A. drummondii*, *A. nitida*, *A. nodosa*, *A. pilata*, *A. pycnostachya* (Benth.) S.W.L.Jacobs & J.Everett, *A. scabra*, *A. scabra* subsp. *falcata, A. trichophylla*, *A. tenuifolia* (Steud.) S.W.L.Jacobs & J.Everett, *A. variabilis* (Hughes) S.W.L.Jacobs & J.Everett (99/99/1.00), (6) *A. feresetacea* (Vickery, S.W.L.Jacobs & J.Everett) S.W.L.Jacobs & J.Everett, *A. setacea* (R.Br.) S.W.L.Jacobs & J.Everett (100/100/1.00) and (7) *A. geoffreyi* S.W.L.Jacobs & J.Everett, *A. juncifolia*, *A. stipoides* (100/100/1.00). The remaining species formed a larger clade asterisked in Figure 2 (80/71/1.00). More or less supported internal clades consisted of (1) *A. exilis* (Vickery) S.W.L.Jacobs & J.Everett and *A. flavescens* (Labill.) S.W.L.Jacobs & J.Everett (56/57/1.00), (2) *A. puberula* (Steud.) S.W.L.Jacobs & J.Everett and *A. velutina* (Vickery, S.W.L.Jacobs & J.Everett) S.W.L.Jacobs & J.Everett (97/77/0.71) together with *A*. *blackii* (C.E.Hubb.) S.W.L.Jacobs & J.Everett and *A*. *mundula* (J.M.Black) S.W.L.Jacobs & J.Everett (64/−/0.85), (3) *A*. *aristiglumis* (F.Muell.) S.W.L.Jacobs & J.Everett and *A*. *gibbosa* (Vickery) S.W.L.Jacobs & J.Everett (88/76/0.97), *A*. *curticoma* (Vickery) S.W.L.Jacobs & J.Everett and *A. stuposa* (Hughes) S.W.L.Jacobs & J.Everett (99/98/1.00) together with *A. bigeniculata* (Hughes) S.W.L.Jacobs & J.Everett (68/76/1.00), (5) *A. petraea* and *A. pubescens* (R.Br.) S.W.L.Jacobs & J.Everett (74/68/0.98), and (6) *A*. *hemipogon*, *A*. *mollis*, *A*. *oligostachya*, *A*. *semibarbata* (81/79/0.99).

#### Nuclear DNA – Single-Copy Locus *Acc1*

The *Acc1* DNA dataset of 73 sequences from 33 species and subspecies included 1512 aligned positions (sequence lengths 1329–1458 bp), of which 568 were variable (38%) and 348 parsimony-informative (23%).

**FIGURE 3:**
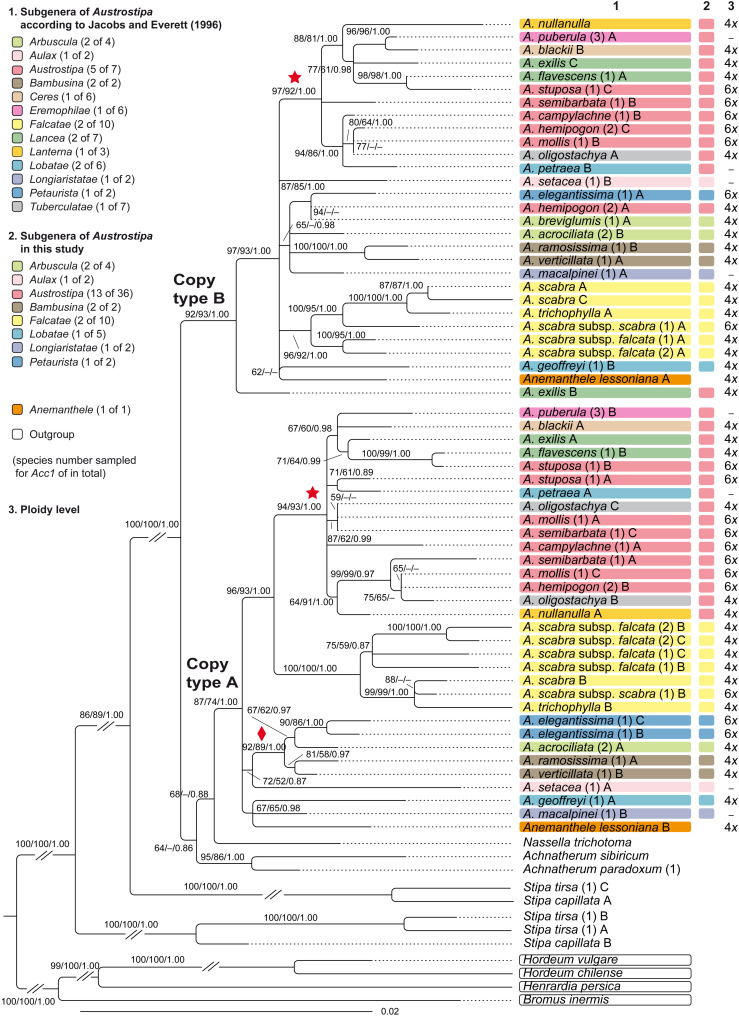
Maximum likelihood phylogram of *Austrostipa* species, *Anemanthele lessoniana* and exemplary other taxa of tribe Stipeae (*Achnatherum* spp., *Nassella trichotoma*, *Stipa* spp.) inferred from DNA sequences of the nuclear single-copy locus *Acc*1 (exon 6–13) with *Bromus inermis* (Bromeae), *Henrardia persica* and *Hordeum* spp. (both Triticeae) used as outgroup. ML and MP bootstrap support values ≥ 50% as well as Bayesian PP ≥ 0.5 are indicated on the branches. Clades with ML support < 50% are collapsed. The asterisked clade within the copy type A and B clades was recovered also in the ITS tree of Figure 2 as well as the clade with diamond. The taxonomic groupings of the *Austrostipa* species according to Jacobs and Everett (1996) and this study are marked by different colors in columns 1 and 2. The ploidy levels of *Austrostipa* taxa and *Anemanthele lessoniana* is given in column 3 according to Winterfeld et al. (2015) and Supplementary Appendix 2. Numbers in brackets after taxon names refer to individual accessions, and different *Acc1* sequence copies are labeled by uppercase letters as listed in the Supplementary Appendix 1. *A*., *Austrostipa*.

The phylogram of the single-copy region *Acc1* with *Bromus inermis*, *Henrardia persica*, *Hordeum chilense*, and *H. vulgare* as outgroup showed species of *Stipa* s.str. sister to a clade comprising all other stipoid taxa (*Achnatherum*, *Anemanthele*, *Austrostipa, Nassella*) (Figure 3). We identified two different copy types of *Acc1* for the tetraploids *S. capillata* and *S. tirsa* (Figure 3), which resulted in the formation of two separate clades that were not sister (both 100/100/1.00). One of these *Stipa* copy type clades was sister to the strongly supported clade with all *Acc1* copy types of *Achnatherum*, *Anemanthele* and *Austrostipa* (100/100/1.00). The *Acc1* copy types of *Anemanthele* and *Austrostipa* segregated into two lineages, copy type A and B in Figure 3. The diploids of *Achnatherum* (*A. paradoxum*, *A. sibiricum*) as well as polyploid *Nassella trichotoma* (see Supplementary Appendix 2) had only a single copy type of *Acc1*. They formed a basal grade to the well-supported copy type A Australasian clade of *Anemanthele* and *Austrostipa* (87/74/1.00).

Copy type A clade comprised three subclades in a polytomy, namely (1) *Anemanthele lessoniana*, *Austrostipa geoffreyi*, and *A. macalpinei* (67/65/0.98), (2) *A. acrociliata*, *A. elegantissima*, *A. ramosissima*, *A. setacea*, and *A. verticillata* (92/89/1.00) and (3) a clade (96/93/1.00) with all six *A. scabra* accessions plus *A. trichophylla* (100/100/1.00) and another clade (94/93/100) with some well-supported minor lineages. Copy type B clade showed *A. exilis* sister to a larger polytomy encompassing *Anemanthele lessoniana* and the remaining species of *Austrostipa*, organized in several minor lineages. *Austrostipa breviglumis*, which had only one *Acc1* clone sequence, was placed in the copy type B clade. In both clades (copy type A and B), the accessions of *A. scabra* and *A. trichophylla* as well as *A. ramosissima* and *A. verticillata* formed supported clades, respectively. These clades, however, were differently placed in the copy type A and B clades, whose general topology was not fully corresponding.

### Morphological Analyses

#### Lemma Epidermal Patterns

**FIGURE 4:**
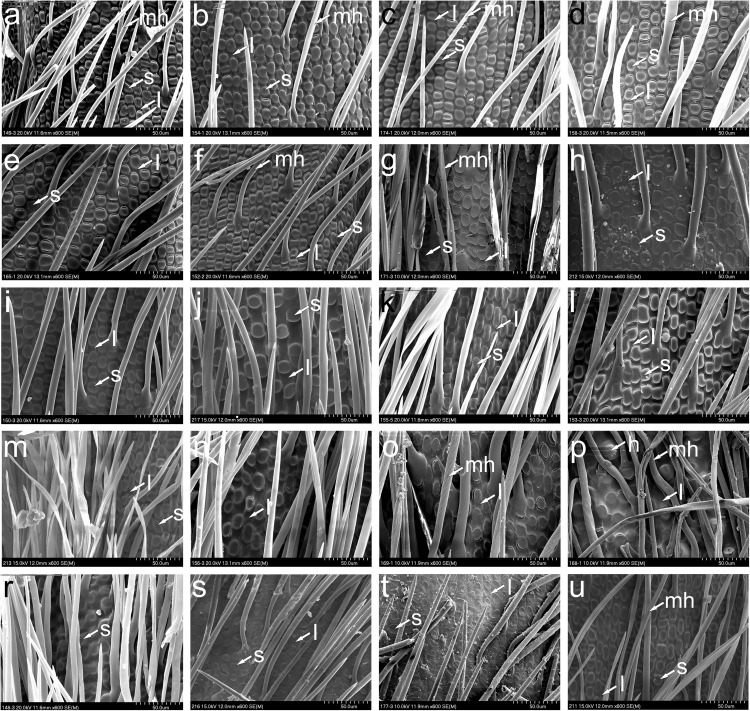
SEM morphology of lemma epidermal patterns in *Austrostipa*. **(a)** *A. nitida*; **(b)** *A. nodosa*; **(c)** *A. scabra* subsp. *scabra*; **(d)** *A. scabra* subsp. *falcata*; **(e)** *A. variabilis*; **(f)** *A. drummondii*; **(g)** *A. stuposa*; **(h)** *A. campylachne*; **(i)** *A. mollis*; **(j)** *A. densiflora*; **(k)** *A. hemipogon*; **(l)** *A. semibarbata*; **(m)** *A. eremophila*; **(n)** *A. setacea*; **(o)** *A. bigeniculata*; **(p)** *A. aristiglumis*; **(r)** *A. gibbosa*; **(s)** *A. compressa*; **(t)** *A. macalpinei*; **(u)** *A. flavescens*. l, long cell (fundamental cell); s, silica cell; h, hook; mh, macrohair. The list of specimens studied is presented in Supplementary Appendix 1.

**FIGURE 5:**
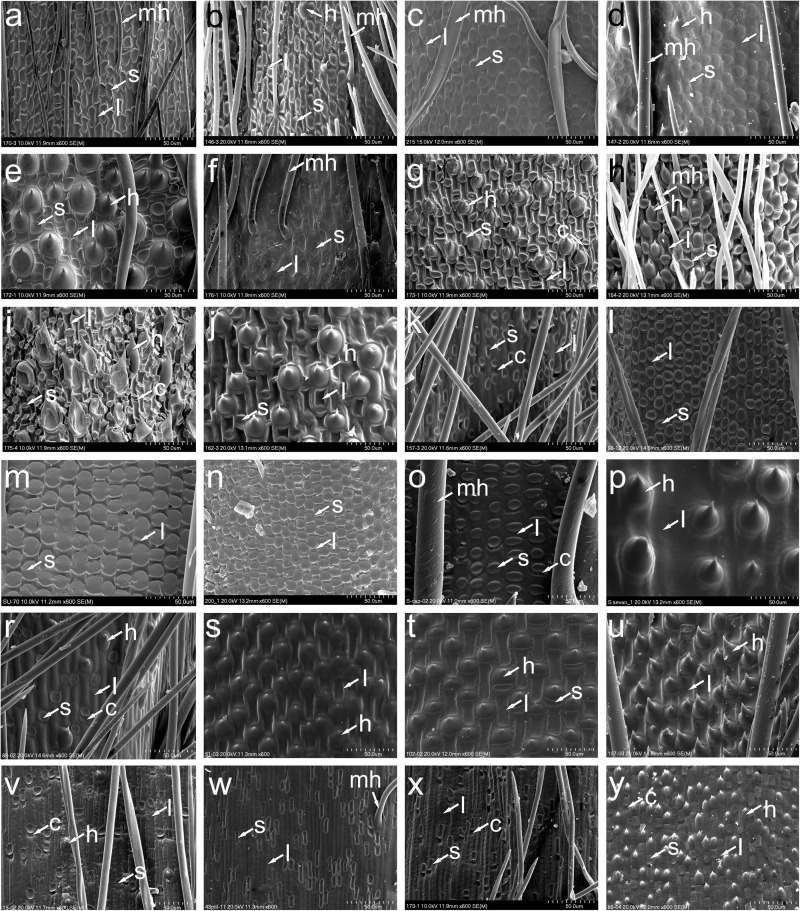
SEM morphology of lemma epidermal patterns of *Austrostipa* and exemplary other genera of Stipeae. **(a)** *A. breviglumis*; **(b)** *A. platychaeta*; **(c)** *A. rudis* subsp. *australis*; **(d)** *A. pubinodis*; **(e)** *A. pubescens*; **(f)** *A. muelleri*; **(g)** *A. ramosissima*; **(h)** *A. verticillata*; **(i)** *A. tuckeri*; **(j)** *A. elegantissima*; **(k)** *A. stipoides*; **(l)** *Achnatherum calamagrostis*; **(m)** *A. bromoides*; **(n)** *A. paradoxum*; **(o)** *Stipellula capensis*; **(p)** *Nassella neesiana*; **(r)** *Macrochloa tenacissima*; **(s)** *Stipa tirsa*; **(t)** *S. kirghisorum*; **(u)** *S. drobovii*; **(v)** *Neotrinia splendens*; **(w)** *Ptilagrostis mongholica*; **(x)** *P. concinna*; **(y)** *Orthoraphium roylei*. l, long cell (fundamental cell); s, silica cell; c, cork cell; h, hook; mh, macrohair. The list of specimens studied is presented in Supplementary Appendix 1.

The lemma epidermal patterns (LEP) found in *Austrostipa* taxa and other Stipeae are illustrated in Figures 4, 5. The patterns for the *Austrostipa* taxa are mapped onto the phylogenetic trees (Figure 2), except for *A. rudis* subsp. *australis* (J.Everett & S.W.L.Jacobs) S.W.L.Jacobs & J.Everett, for which there are no molecular data. Information on the studied specimens is contained in Supplementary Appendix 1. In *Austrostipa*, four LEPs were encountered.

In 28 of the 34 taxa of *Austrostipa* examined, the LEP was relatively uniform (Figures 2, 4a–u, 5a–d,f) and typical of achnatheroid grasses as seen, for example, in *Achnatherum*, *Anemanthele, Jarava* or *Stipellula* (Figures 5l–o). This maize-like LEP is characterized by wider than long, short or square to rectangular fundamental cells with undulate to almost straight side walls. Silica cells were very frequent, ovate to elongate, densely packed and regularly alternating with fundamental cells.

In four of the remaining six species, namely, *A. elegantissima, A. tuckeri*, *A. ramosissima*, and *A. verticillata* (Figures 2, 5g–j), the LEP was distinctively different due to the presence of numerous hooks and longer fundamental cells, here termed ‘prickly maize-like’ LEP, and hence reminiscent of the LEP found in Old World genera such as *Stipa, Neotrinia*, *Orthoraphium* Nees and *Ptilagrostis* (Figures 5s–y). The LEP observed in species of subgenera *Petaurista* S.W.L.Jacobs & J.Everett and *Bambusina* is characterized by short cells with hooks alternating with square or rectangular fundamental cells, ovate silica cells sometimes paired with cork cells, which, however, are generally sparse. Hooks were frequent in *A. pubescens* (subg. *Tuberculatae*; Figure 5e) but scattered in *A. campylachne* (subg. *Austrostipa*); Figure 4h, however, due to their short (wider than long) fundamental cells, we classified them to maize-like LEP group.

The LEP with straight- to slightly sinuously walled fundamental cells observed in *A. stipoides* (subg. *Lobatae* S.W.L.Jacobs & J.Everett; Figures 2, 5k) is characterized by its rectangular to elongated fundamental cells (1.5–4 times as long as wide), which often alternate with silica cells and cork cells as well as sometimes scattered hooks. The elongated silica cells were often associated with cork cells and frequently had 1-4 constrictions.

A characteristic LEP with dumbbell-shaped fundamental cells alternating with elongated silica cells was encountered only in *A. muelleri* (Figure 5f).

#### Combined Analysis of Micro- and Marcomorphological Characters

**FIGURE 6:**
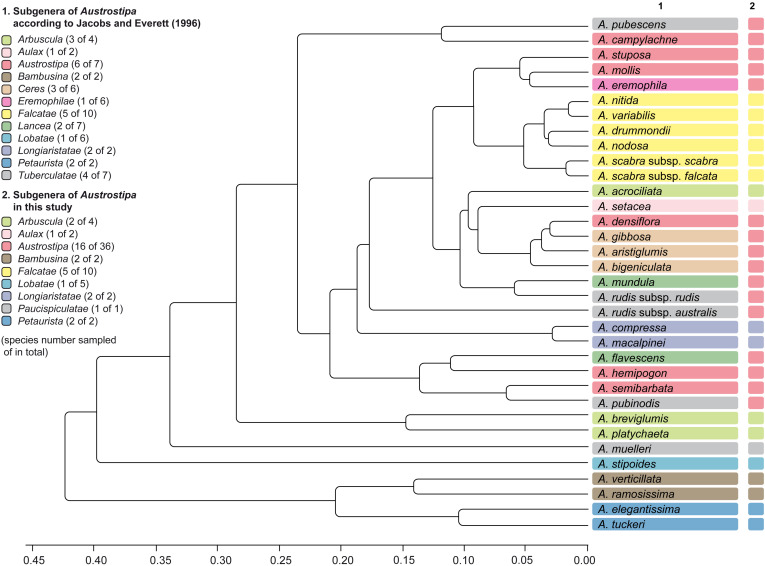
Cluster analysis (UPGMA) performed on eight macro- and four micromorphological characters for 34 *Austrostipa* taxa. See Supplementary Table 2 for the data matrix evaluated. The taxonomic groupings of the *Austrostipa* species according to Jacobs and Everett (1996) and this study are marked by different colors in columns 1 and 2. *A*., *Austrostipa*.

The species representing subgenera *Bambusina, Lobatae*, and *Petaurista* as well as *A. muelleri* were well separated from all other species in the cluster analysis (UPGMA), which was performed on a combined macro- and micromorphological 34-taxa dataset (Figure 6 and Supplementary Figure 2).

Similar results were obtained when the micro- and macromorphological characters were analyzed separately (34- and 65-taxa set, respectively; Supplementary Figures 3, 4 and Supplementary Tables 1, 2). The unique LEP of *A. stipoides* and presence of distinct lobes on the top of the lemma (*A. juncifolia*, *A. geoffreyi, A. petraea*) separated these four species of subg. *Lobatae* from the remaining subgenera of *Austrostipa* (Figure 6 and Supplementary Figures 3, 4). A cluster was formed by representatives of subgenera *Petaurista* and *Bambusina*, which had prickly maize-like LEP (Figure 6 and Supplementary Figure 3). The unique macromorphology of the inflorescences characterized by long pilose branches, which occurred exclusively in *A. elegantissima* and *A. tuckeri* (subg. *Petaurista*), resulted in a clear separation from the other subgenera of *Austrostipa* (Supplementary Figure 4). Due to its long apical lemma lobes as well as its particular LEP, *A. muelleri* was well-distinguished not only from the other representatives of subg. *Tuberculatae* but from all other studied species with achnatheroid, maize-like LEP (Figure 6 and Supplementary Figure 4). The species of the remaining subgenera of *Austrostipa* with maize-like LEP were grouped in several (sub)clusters in accordance to each of the three performed analyses, with rather weakly noticeable subgeneric ordination (Figure 6 and Supplementary Figures 3, 4).

### Chromosome Numbers and Whole-Genome Duplications in Stipeae

**FIGURE 7:**
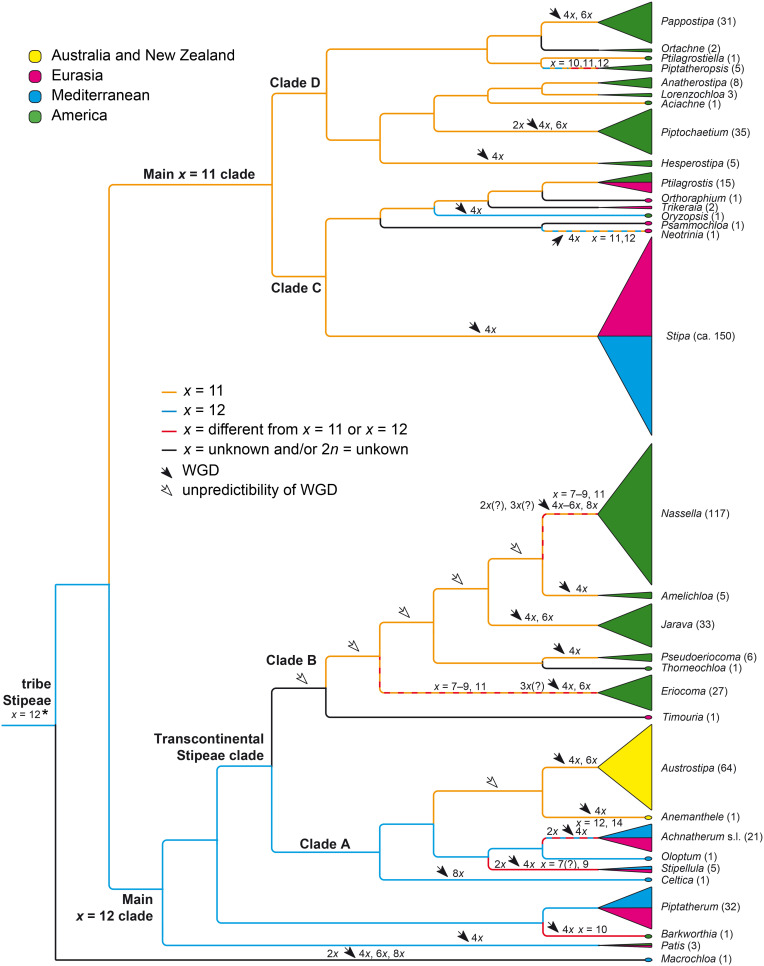
Chromosome base numbers, dysploidy and whole-genome duplications in the evolution of tribe Stipeae mapped on a simplified molecular phylogenetic tree with the genera of Stipeae and their approximate sizes. The cladogram is modified from the plastid DNA tree of Romaschenko et al. (2012) and the plastid/nuclear DNA tree (concatenated data of congruent taxa) of Hamasha et al. (2012). The treatment of genera and estimated number of species largely follows Peterson et al. (2019). For chromosome data see Supplementary Appendix 2. The species number of a genus is indicated after the genus name. Due to the unknown chromosome numbers in *Thorneochloa* and *Timouria* it is unclear at which split in Clade B whole-genome duplications occurred, potentially already in the common ancestor of all Clade B taxa (open arrows). The asterisk denotes our suggested most likely chromosome base number (see section “Discussion”). WGD, whole-genome duplication.

The chromosome numbers of species and genera in Stipeae are listed in Supplementary Appendix 2 (bold print). For each of the 33 genera, the most frequently found chromosome numbers are underlined, if applicable. In six genera the chromosome numbers is yet unknown (*Ortachne* Nees, *Orthoraphium*, *Psammochloa* Hitchc., *Thorneochloa* Romasch., P.M.Peterson & Soreng, *Timouria* Roshev., *Trikeraia* Bor). Monoploid chromosome numbers, chromosome base numbers (*x* =) and ploidy levels deduced from the chromosome counts in the Supplementary Appendix 2 were added to Figure 7. This figure represents a simplified phylogenetic tree (cladogram) portraying the genera of Stipeae, their approximate sizes and distribution (see section “Materials and Methods” and legend to Figure 7 for further explanation).

*Piptatherum* (32 species) and *Ptilagrostis* (15) are the largest genera with only diploids known (Supplementary Appendix 2). Only two small further genera have only diploids, namely *Ortachne* (2 species) and *Oloptum* (1 species). *Piptochaetium* (35 species) and *Achnatherum* (21 species) have prevailingly diploids but also polyploids (Supplementary Appendix 2). The 20 remaining genera are a consistently polyploid (2*n* = 4*x*–8*x*), including *Stipa* (ca. 150 species), the largest genus of Stipeae with seemingly consistently 2*n* = 4*x* = 44 and *Austrostipa* with almost consistently 2*n* = 4*x* = 44 and 6*x* = 66 (Supplementary Appendix 2 and Figure 7). *Nassella*, the second largest genus with 117 species, encompasses 4*x*, 6*x*, and 8*x* taxa and heteroploid crosses with seemingly 5*x* but also many species with lower chromosome numbers of 2*n* = 26–28 and 2*n* = 34–36 that might represent diploids or triploids derived from chromosome base numbers lower than *x* = 11, for example, *x* = 7–9 (Figure 7) or alternatively were derived from *x* = 11 via descending dysploidy (see below section “Discussion”). *Eriocoma* (27 species) also has a comparatively large range of chromosome numbers, however, the lowest numbers are 2*n* = 32–36 (Supplementary Appendix 2, see below).

The prevailing chromosome base number in Stipeae is *x* = 11, marked as orange lines in Figure 7. It occurs in Australasian *Austrostipa* and *Anemanthele*, in the genera of New World Clade B and the Main *x* = 11 clade but not consistently in four of their genera (*Eriocoma*, *Nassella*, *Neotrinia*, *Piptatheropsis* Romasch., P.M.Peterson & Soreng; hatched lines in Figure 7) and except for *Oryzopsis* Michx. with *x* = 12 (Figure 7). The number of *x* = 12 (blue lines) is less frequent and occurs in eight genera but not consistently in three of them (hatched lines). Interestingly, the *x* = 11 genera *Austrostipa* and *Anemanthele* are placed in a clade with mainly *x* = 12. *Barkworthia* Romasch., P.M.Peterson & Soreng (1 species) and *Stipellula* (5 species) consistently have deviant numbers of *x* = 10 and *x* = 7(?), 9, respectively (Supplementary Appendix 2 and Figure 7).

In some instances, the occurrence of whole genome duplications (WGD) could be labeled on the tree (filled arrows in Figure 7; see below section “Discussion”). Due to missing chromosome number information for *Thorneochloa* and *Timouria* and the uncertain occurrence of diploids in *Nassella* and *Eriocoma* (triploids as hybrids involving putative diploids), it is impossible to attach WGD in Clade B to particular nodes (open arrows in Figure 7).

## Discussion

### Molecular Phylogenetic Delineation of *Austrostipa*

Monophyly of *Austrostipa* was not clearly supported by any of the three DNA regions we investigated (plastid 3′*trnK* region: Figure 2 and Supplementary Figure 1; nr ITS region: Figure 2 and Supplementary Figure 2; the single-copy locus *Acc1*: Figure 3). The plastid DNA trees showed *Austrostipa* as paraphyletic. Most species (36/43) belonged either to the large clade of ‘core *Austrostipa*’ or to the clade comprising *A. drummondii*, *A. muelleri*, *A. nitida*, *A. pilata.* These two clades formed a polytomy with the three remaining species of *Austrostipa* (*A. macalpinei*, *A. ramosissima*, and *A. verticillata*) and three Eurasian stipoid genera (*Achnatherum*, *Oloptum*, and *Stipellula*). *Anemanthele*, however, was not part of this polytomy, but of the next lower one which also included the two representatives of the primarily South American genus *Nassella* and the western Mediterranean *Celtica gigantea*.

Possible explanations for the failure of the plastid data to support, even weakly, the monophyly of *Austrostipa* include incomplete lineage sorting (ILS) affecting the inheritance of plastids or genetic introgression from the Eurasian species into the three *Austrostipa* species in the lowest *Austrostipa-*containing clade (*A. macalpinei*, *A. ramosissima, A. verticillata*). This last seems unlikely, given the present day distribution of the species involved. Higher support for monophyly of *Austrostipa* (86/NA/1.00) was recorded for a set of 13 *Austrostipa* species using more than 6.600 aligned plastid bp (Romaschenko et al., 2012) and *Anemanthele* was weakly supported sister (52/NA/0.95).

Nuclear ITS grouped *Austrostipa* (all species) and *Anemanthele* in a single clade but support was minimal for the relationship (see reduced dataset with each species represented only by a single accession in Figure 2 and in Supplementary Figure 2 with all accessions studied). These results are similar to those obtained by Romaschenko et al. (2012).

*Austrostipa* and *Anemanthele* were alike in having two different copies of the nuclear gene *Acc1* (copy types A and B). These resolved together in two separate clades (Figure 3). The copies obtained for *Achnatherum paradoxum*, *A. sibiricum* and *Nassella trichotoma* were close to the copy type A clade; those for the two species of *Stipa* included (*S. capillata*, *S. tirsa*) divided likewise into two copy types, both of which were outside the two *Austrostipa* clades (Figure 3).

Our results, in failing to contradict or providing only weak support for the monophyly of *Austrostipa* and the closer relationship of *Austrostipa* to *Anemanthele* rather than non-Australasian stipoids, basically agrees with the findings of several previous studies regardless of taxon sampling (Jacobs et al., 2000, 2007; Barkworth et al., 2008; Romaschenko et al., 2010, 2012; Syme, 2011; Syme et al., 2012; Hamasha et al., 2012). The odd results for *Austrostipa stipoides* reported in two studies (Jacobs et al., 2007; Barkworth et al., 2008), which placed the species distant from other species of the genus, were from duplicate collections (Barkworth et al., 2008, p. 725) and were not corroborated by this study, in which three different collections were used (see Supplementary Appendix 1). Their plastid and nuclear DNA sequences clustered with those of other *Austrostipa* species (Figures 2, 3 and Supplementary Figures 1, 2), as did sequences from the specimens of *A. stipoides* studied by Syme (2011) and Syme et al. (2012).

### Phylogenetic Differentiation in *Austrostipa* and Taxonomy

All but one of the subgenera of *Austrostipa* were represented by two representatives for at least one of the sequences we examined (Table 1). The exception was subg. *Lanterna*, which means we cannot comment on its monophyly.

#### Comparison of Plastid and nr ITS Tree

The plastid and nr ITS trees showed slightly different placements of subgenera *Bambusina* and *Longiaristatae* S.W.L.Jacobs & J.Everett. *Austrostipa ramosissima* and *A. verticillata* (subg. *Bambusina*) and *A. macalpinei* (subg. *Longiaristatae*) were placed in the plastid DNA tree in a polytomy with the remainder of *Austrostipa* and other genera of Stipeae (*Achnatherum*, *Anemanthele*, *Oloptum*, and *Stipellula*). This was not reflected in the ITS tree, where all studied *Austrostipa* subgenera were resolved in a weakly supported clade together with *Anemanthele*. Both subgenera (*Bambusina, Longiaristatae*) resolved as monophyletic considering also *A. compressa* (subg. *Longiaristatae*), which was sampled only for ITS DNA. Sampling more DNA regions could improve overall resolution of the plastid DNA phylogenetic tree.

A small clade of *Austrostipa* species in the plastid DNA tree comprised species of two different subgenera, namely three species of the large subgenus *Falcatae* and *A. muelleri* of subg. *Tuberculatae* (see also below). Both subgenera were represented also in the ‘core *Austrostipa*’ clade of the plastid DNA tree with the remaining *Austrostipa* species (Figure 2 and Supplementary Figure 1). The subgenus *Falcatae*, however, was resolved in the ITS tree as monophyletic, whereas *Tuberculatae* were highly polyphyletic. In other words, subg. *Falcatae* is characterized by remarkable cytonuclear discordance, having at least two different chloroplast ‘types.’ Ancient polymorphism, hybridization and introgression may be its potential causes of such discordance as encountered in many groups of angio- and gymnosperms (Rieseberg and Soltis, 1991; Seehausen, 2004; Folk et al., 2017; Kawabe et al., 2018; Tkach et al., 2020).

The weakly supported clade marked by a diamond in the ITS tree of Figure 2 united species of three subgenera resolved as monophyletic: *Bambusina*, *Arbuscula*, and *Petaurista*. This diamond-marked clade, however, was not recovered in the plastid DNA tree, and altogether three different plastid types occurred in this instance.

Except for small subg. *Aulax* S.W.L.Jacobs & J.Everett with both of its species sampled for ITS, none of the further *Austrostipa* subgenera encompassing several species resolved in our plastid and ITS DNA analyses as monophyletic, viz. *Arbuscula*, *Austrostipa*, *Ceres* S.W.L.Jacobs & J.Everett, *Eremophilae* S.W.L.Jacobs & J.Everett, *Lancea* S.W.L.Jacobs & J.Everett, *Lobatae* and *Tuberculatae*, which were para- or polyphyletic or placed in polytomies (Figure 2 and Supplementary Figures 1, 2).

#### Single-Copy Locus *Acc1*

The sequences analyses of the *Acc1*, a gene represented by a single copy per monoploid genome (see section “Results”), corroborated monophyly of subgenera *Bambusina* and *Falcatae*, whereas subgenera *Arbuscula*, *Austrostipa*, *Lancea*, and *Lobatae* were non-monophyletic (Figure 3). Within copy type A clade, the asterisked clade supported by 94/93/1.00 (Figure 3) comprised species belonging to subgenera *Austrostipa*, *Ceres*, *Eremophilae*, *Lancea*, *Lanterna*, *Lobatae*, and *Tuberculatae*. This clade was largely reflected also in the copy type B topology (asterisked; 97/92/1.00). *Austrostipa exilis* (accession shown to be tetraploid with 2*n* = 44; Supplementary Appendix 2; Winterfeld et al., 2015) and *A. hemipogon* (accession shown to be hexaploid with 2*n* = 66; Supplementary Appendix 2; Winterfeld et al., 2015) have additional copies of *Acc1* gene copy type B. That for *A. exilis* was placed external to all other *Australasian* stipoids in the tree (Figure 3). The asterisked clades in *Acc1* copy type A and B clades corresponded well with the asterisked clade supported by 80/71/1.00 in the ITS tree (Figure 2), thus there is consistent phylogenetic signal in both nuclear markers studied.

Polyploidy, whether allo- or autopolyploidy, is difficult to recognize in Stipeae by ITS analysis. The occurrence of different *Acc1* copies (labeled as A, B, C in Figure 3) belonging to two copy types in the specimens of *Anemanthele* (4*x*), *Austrostipa* (4*x*–6*x*), and *Stipa* (4*x*) suggests consistent allopolyploidy of these genera. The presence of more than two *Acc1* copies in some tetraploids (e.g., *Austrostipa exilis*, *A. oligostachya*) rests presumably on duplicated gene loci. The data on the different gene copies provides molecular evidence of allopolyploidy in the mentioned genera of Stipeae. Allopolyploidy as suggested by sequence analyses of a nuclear gene (*At103*) has also been reported in the East Asian/North American stipoid genus *Patis* Ohwi (Romaschenko et al., 2014).

#### Phylogenetic Utility of Micromorphological Traits

In 32 of the 34 micromorphologically studied *Austrostipa* taxa, twelve of which were investigated for the first time for lemma epidermal characters, the LEP was maize-like, typical for achnatheroid grasses, with dominance of silica cells and with fundamental cells shorter, as long as wide up to 2–3 times longer than wide, in *A. densiflora* even (1–)2–4 times longer than wide (see also Bustam, 2012, Figure A2–7). The prevalence of this maize-like LEP corroborates the previous results for Australian feathergrasses (Barkworth and Everett, 1987; Romaschenko et al., 2010, 2012; Bustam, 2012).

*Austrostipa ramosissima* and *A. verticillata* (subg. *Bambusina*) as well as *A. elegantissima* and *A. tuckeri* (subg. *Petaurista*) have a large number of conspicuous hooks in the middle part of lemma in addition to rectangular fundamental cells and rounded silica cells associated with cork cells (prickly maize-like LEP), which was not seen in the other *Austrostipa* taxa characterized by typical maize-like LEP. In the upper part of the lemma, however, most *Austrostipa* taxa have a mixture of hooks alternating with shorter to equal, rarely somewhat longer than wide fundamental cells in addition to prickles, bicellular hairs and macrohairs.

These four species together with *A. acrociliata*, *A. breviglumis*, and *A. platychaeta* (subg. *Arbuscula*) were placed by Barkworth and Everett (1987) in their taxonomic group 2 of the Australian Stipeae (Figure 2), considering for classifications not only LEPs but also a set of macromorphological characters. This group 2 is reflected by the diamond-marked clade in our nr ITS tree (Figure 2). Based on extremely long fundamental cells, Barkworth and Everett (1987) distinguished their group 5 including two species *A. setacea* and *A. feresetacea*. This group (subg. *Aulax*) was corroborated as monophyletic based on the ITS data (Figure 2) but was not resolved by the cluster analyses using morphological characters (Supplementary Figure 4). According to Barkworth and Everett (1987), *A. setacea* and *A. feresetacea* should have fundamental cells 3–4 times longer than silica cells, however, they were shorter in our studied specimens of *A. setacea* (only 1–3 times longer), as depicted also in Bustam (2012, Figures A2–28). Unfortunately, *A. feresetacea* was not available for this study.

The LEPs of *A. stipoides* and *A. muelleri* were strikingly different from that of all other *Austrostipa* taxa. Having rather long fundamental cells with elongated silica cells associated with cork cells, the LEP of *A. stipoides* (SWF) was somewhat more similar to *Ptilagrostis* than to the other examined *Austrostipa* taxa. The overall appearance resembles the saw-like LEP but the side walls of the fundamental cells were straight to slightly sinuate, not deeply sinuous as in the typical saw-like LEP (Barkworth and Everett, 1987; Romaschenko et al., 2010, 2012; Nobis et al., 2019b,c; Figures 5r,v,w,x). *Austrostipa stipoides* was the only representative of subgenus *Lobatae* we studied for LEP. Two further species (*A. geoffreyi* and *A. juncifolia*) were studied by Bustam (2012), and their fundamental cells also seem to be two or more times longer than wide. However, the details of the lemma epidermis are hardly discernible on the photographs presented in this publication.

*Austrostipa muelleri*, characterized by a unique LEP with dumbbell-shaped fundamental cells and elongated silica cells, is the only species of traditional subgenus *Tuberculatae* with distinct apical lobes on the lemma apex, otherwise found only in subgenus *Lobatae*. This segregation seems to fit the placement of *A. muelleri* distant to remainder of the subgenus *Tuberculatae* in the phylogenetic trees (Figure 2; see below).

#### Delineation and Relationship of Subgenera

Despite limited resolution achieved by the sequenced plastid and nr DNA loci as well as the combined macro- and micromorphological analysis, some conclusions can be drawn with respect to the infrageneric taxonomy of *Austrostipa* and the validity of the altogether 13 subgenera presented in Vickery et al. (1986), Jacobs and Everett (1996), and Everett et al. (2009), all of which were included in this study.

(1) The small subgenera *Longiaristatae* (both species sampled, plastid DNA data missing for *A. compressa*) and *Bambusina* (both species sampled) belong to the early branching lineages within *Austrostipa* considering the plastid DNA tree. Subg. *Bambusina* assembled together with subgenera *Petaurista* (both species sampled) and *Arbuscula* (three of four species sampled) in the same ITS and in copy type A clades of the *Acc1* gene analyses marked by diamonds (Figures 2, 3). *Petaurista* and *Arbuscula* were placed in the ‘core *Austrostipa*’ clade of the plastid DNA tree distantly to the species of subg. *Bambusina*. Maintenance of subgenera *Petaurista* and *Arbuscula* is neither explicitly supported nor contradicted by our data. Thus, we argue that these four subgenera should remain unchanged.
(2) *Austrostipa muelleri* was placed distantly from all other taxa of subg. *Tuberculatae* (see below), in which it was accommodated (Jacobs and Everett, 1996; Everett et al., 2009). This deviating position was noted already previously (Jacobs et al., 2007, Figure 4; Syme et al., 2012). We propose placing *A. muelleri* by itself in a new subgenus (see below New names and combinations).
(3) Subg. *Falcatae* (9 of 10 species sampled, plastid DNA data missing for *A. pycnostachya* and *A. tenuifolia*) was supported because of the ITS and *Acc1* DNA data (Copies A and B) but it disintegrated into two lineages of the plastid DNA phylogeny. One group of species possessed the ‘core *Austrostipa*’ plastid, the other shared a deviant plastid type with *A. muelleri* (Figure 2 and Supplementary Figure 1). The placement of *A. pycnostachya* in the ITS clade of subg. *Falcatae* (Figure 2) supports the transfer of this species from subg. *Arbuscula*, in which it was placed by Jacobs and Everett (1996), to subg. *Falcatae* (Everett et al., 2009).
(4) Subg. *Aulax* (both species sampled, plastid DNA data missing for *A. feresetacea*) and subg. *Lobatae* (4 of 6 species sampled) could be maintained after excluding *A. petraea* from the latter (Figure 2). Segregation of *A. petraea* from the other species of subg. *Lobatae* was noted also by Syme et al. (2012). We found no support, however, for a placement of this species in subg. *Aulax* as suggested by the latter study (see Syme et al., 2012, Figure 1) but the taxonomic position of this comparatively narrowly distributed species of eastern South Australia should be reviewed in future investigations.
(5) The high-support clades asterisked in the ITS and *Acc1* phylograms (Figures 2, 3) encompass, apart from *A. petraea*, the species of subgenera *Austrostipa* (6 of 7 species sampled), *Ceres* (5 of 6 species sampled), *Eremophilae* (5 of 6 species sampled), *Lancea* (six of seven species sampled), *Lanterna* (1 of 3 species sampled) and *Tuberculatae* (5 of 7 species sampled). The asterisked clades showed several sister species relationships and minor lineages within and between subgenera (see above), but none of the subgenera mentioned was resolved as separate lineage, which is in agreement with the trees presented by Syme et al. (2012). For the time being it seems best to assign all these subgenera to a single, expanded and most likely monophyletic subgenus *Austrostipa*. This suggestion, however, should not be interpreted as attempt to supersede traditional morphology-based by molecular phylogenetic taxonomic concepts. It is rather a contribution to obtain monophyletic taxa, which can serve as reliable units addressing questions about character evolution and/or biogeography in *Austrostipa*, which have been barely touched upon to date.

Some of our suggestions for classification are not new, having been made in previous molecular phylogenetic studies of *Austrostipa*, for example, the maintenance of subgenera *Falcatae* (Jacobs et al., 2007; Bustam, 2010, 2012; Syme et al., 2012), *Longiaristatae* and *Lobatae* (Jacobs et al., 2007; Syme et al., 2012), the broadening of subg. *Austrostipa* to include also subgenera *Tuberculatae* (Jacobs et al., 2007; Syme et al., 2012) and *Eremophilae* (Syme et al., 2012), but our data do not support combining subgenera *Arbuscula* and *Bambusina*, a suggestion based on their similar habit (Jacobs et al., 2007).

In summary, we propose dividing of *Austrostipa* into the following nine subgenera (with number of species) (Table 4): *Arbuscula* (4), *Aulax* (2), *Austrostipa* (36), *Bambusina* (2), *Falcatae* (10), *Lobatae* (5), *Longiaristatae* (2), *Petaurista* (2) and *Paucispiculatae*, subg. nov., with *A. muelleri* (1).

**TABLE 4:** Subgenera and species of *Austrostipa* in this study and according to Jacobs and Everett (1996) supplemented by Williams (2011) and informal groups suggested by Barkworth and Everett (1987).

This study		Jacobs and Everett, 1996	Barkworth and Everett, 1987
***Arbuscula*** S.W.L.Jacobs & J.Everett			
	*A. acrociliata* (Reader) S.W.L.Jacobs & J.Everett	*Arbuscula*	2
	*A. breviglumis* (J.M.Black) S.W.L.Jacobs & J.Everett	*Arbuscula*	2
	*A. nullarborensis* (Vickery, S.W.L.Jacobs & J.Everett) S.W.L.Jacobs & J.Everett	*Arbuscula*	2
	*A. platychaeta* (Hughes) S.W.L.Jacobs & J.Everett	*Arbuscula*	2
***Aulax*** S.W.L.Jacobs & J.Everett			
	*A. feresetacea* (Vickery, S.W.L.Jacobs & J.Everett) S.W.L.Jacobs & J.Everett	*Aulax*	5
	*A. setacea* (R.Br.) S.W.L.Jacobs & J.Everett	*Aulax*	5
***Austrostipa***			
	*A. aphylla* (Rodway) S.W.L.Jacobs & J.Everett	*Tuberculatae*	3
	*A. aquarii* (Vickery, S.W.L.Jacobs & J.Everett) S.W.L.Jacobs & J.Everett	*Austrostipa*	1
	*A. aristiglumis* (F.Muell.) S.W.L.Jacobs & J.Everett	*Ceres*	1
	*A. bigeniculata* (Hughes) S.W.L.Jacobs & J.Everett	*Ceres*	1
	*A. blackii* (C.E.Hubb.) S.W.L.Jacobs & J.Everett	*Ceres*	1
	*A. campylachne* (Nees) S.W.L.Jacobs & J.Everett	*Austrostipa*	1
	*A. centralis* (Vickery, S.W.L.Jacobs & J.Everett) S.W.L.Jacobs & J.Everett	*Eremophilae*	1
	*A. crinita* (Gaudich.) S.W.L.Jacobs & J.Everett	*Lancea*	1
	*A. curticoma* (Vickery) S.W.L.Jacobs & J.Everett	*Ceres*	1
	*A. densiflora* (Hughes) S.W.L.Jacobs & J.Everett	*Austrostipa*	1
	*A. dongicola* (Vickery, S.W.L.Jacobs & J.Everett) S.W.L.Jacobs & J.Everett	*Ceres*	1
	*A. echinata* (Vickery, S.W.L.Jacobs & J.Everett) S.W.L.Jacobs & J.Everett	*Lancea*	1
	*A. eremophila* (Reader) S.W.L.Jacobs & J.Everett	*Eremophilae*	1
	*A. exilis* (Vickery) S.W.L.Jacobs & J.Everett	*Lancea*	1
	*A. flavescens* (Labill.) S.W.L.Jacobs & J.Everett	*Lancea*	1
	*A. gibbosa* (Vickery) S.W.L.Jacobs & J.Everett	*Ceres*	1
	*A. hemipogon* (Benth.) S.W.L.Jacobs & J.Everett	*Austrostipa*	–
	*A. lanata* (Vickery, S.W.L.Jacobs & J.Everett) S.W.L.Jacobs & J.Everett	*Lanterna*	1
	*A. metatoris* (J.Everett & S.W.L.Jacobs) S.W.L.Jacobs & J.Everett	*Eremophilae*	1
	*A. mollis* (R.Br.) S.W.L.Jacobs & J.Everett	*Austrostipa*	1
	*A. multispiculis* (J.M.Black) S.W.L.Jacobs & J.Everett	*Lancea*	1
	*A. mundula* (J.M.Black) S.W.L.Jacobs & J.Everett	*Lancea*	1
	*A. nivicola* (J.H.Willis) S.W.L.Jacobs & J.Everett	*Tuberculatae*	3
	*A. nullanulla* (J.Everett & S.W.L.Jacobs) S.W.L.Jacobs & J.Everett	*Lanterna*	1
	*A. oligostachya* (Hughes) S.W.L.Jacobs & J.Everett	*Tuberculatae*	3
	*A. petraea* (Vickery) S.W.L.Jacobs & J.Everett	*Lobatae*	1
	*A. plumigera* (Hughes) S.W.L.Jacobs & J.Everett	*Eremophilae*	1
	*A. puberula* (Steud.) S.W.L.Jacobs & J.Everett	*Eremophilae*	1
	*A. pubescens* (R.Br.) S.W.L.Jacobs & J.Everett	*Tuberculatae*	3
	*A. pubinodis* (Trin. & Rupr.) S.W.L.Jacobs & J.Everett	*Tuberculatae*	3
	*A. rudis* (Spreng.) S.W.L.Jacobs & J.Everett	*Tuberculatae*	3
	*A. rudis* subsp. *australis* (J.Everett & S.W.L.Jacobs) S.W.L.Jacobs & J.Everett	*Tuberculatae*	3
	*A. rudis* subsp. *nervosa* (Vickery) S.W.L.Jacobs & J.Everett	*Tuberculatae*	3
	*A. semibarbata* (R.Br.) S.W.L.Jacobs & J.Everett	*Austrostipa*	1
	*A. stuposa* (Hughes) S.W.L.Jacobs & J.Everett	*Austrostipa*	1
	*A. velutina* (Vickery, S.W.L.Jacobs & J.Everett) S.W.L.Jacobs & J.Everett	*Lancea*	1
	*A. vickeryana* (J.Everett & S.W.L.Jacobs) S.W.L.Jacobs & J.Everett	*Lanterna*	1
	*A. wakoolica* (Vickery, S.W.L.Jacobs & J.Everett) S.W.L.Jacobs & J.Everett	*Eremophilae*	1
***Bambusina*** S.W.L.Jacobs & J.Everett			
	*A. ramosissima* (Trin.) S.W.L.Jacobs & J.Everett	*Bambusina*	2
	*A. verticillata* (Nees ex Spreng.) S.W.L.Jacobs & J.Everett	*Bambusina*	2
***Falcatae*** S.W.L.Jacobs & J.Everett			
	*A. blakei* (Vickery, S.W.L.Jacobs & J.Everett) S.W.L.Jacobs & J.Everett	*Falcatae*	4
	*A. drummondii* (Steud.) S.W.L.Jacobs & J.Everett	*Falcatae*	4
	*A. nitida* (Summerh. & C.E.Hubb.) S.W.L.Jacobs & J.Everett	*Falcatae*	4
	*A. nodosa* (S.T.Blake) S.W.L.Jacobs & J.Everett	*Falcatae*	4
	*A. pilata* (S.W.L.Jacobs & J.Everett) S.W.L.Jacobs & J.Everett	*Falcatae*	4
	*A. pycnostachya* (Benth.) S.W.L.Jacobs & J.Everett	*Falcatae*	4
	*A. scabra* (Lindl.) S.W.L.Jacobs & J.Everett	*Falcatae*	4
	*A. scabra* subsp. *falcata* (Hughes) S.W.L.Jacobs & J.Everett	*Falcatae*	4
	*A. tenuifolia* (Steud.) S.W.L.Jacobs & J.Everett	*Falcatae*	4
	*A. trichophylla* (Benth.) S.W.L.Jacobs & J.Everett	*Falcatae*	4
	*A. variabilis* (Hughes) S.W.L.Jacobs & J.Everett	*Falcatae*	4
***Lobatae*** S.W.L.Jacobs & J.Everett			
	*A. bronwenae* A.R.Williams	*Lobatae*	–
	*A. geoffreyi* S.W.L.Jacobs & J.Everett	*Lobatae*	–
	*A. jacobsiana* A.R.Williams	*Lobatae*	–
	*A. juncifolia* (Hughes) S.W.L.Jacobs & J.Everett	*Lobatae*	1
	*A. stipoides* (Hook.f.) S.W.L.Jacobs & J.Everett	*Lobatae*	1
***Longiaristatae*** S.W.L.Jacobs & J.Everett			
	*A. compressa* (R.Br.) S.W.L.Jacobs & J.Everett	*Longiaristatae*	1
	*A. macalpinei* (Reader) S.W.L.Jacobs & J.Everett	*Longiaristatae*	1
***Petaurista*** S.W.L.Jacobs & J.Everett			
	*A. elegantissima* (Labill.) S.W.L.Jacobs & J.Everett	*Petaurista*	2
	*A. tuckeri* (F.Muell.) S.W.L.Jacobs & J.Everett	*Petaurista*	2
***Paucispiculatae***, subg. nov.			
	*A. muelleri* (Tate) S.W.L.Jacobs & J.Everett	*Tuberculatae*	3

### Chromosome Base Numbers and Whole-Genome Duplications in Stipeae

#### *Austrostipa* and *Anemanthele*

The somatic chromosome numbers of 2*n* = 44 and 2*n* = 66 were established in 18 and in seven *Austrostipa* species, respectively, as well as 2*n* = 44 in *Anemanthele lessoniana* in our previous study on chromosome numbers and karyotypes (Winterfeld et al., 2015). These results corroborated the earlier chromosome counts in *Austrostipa stipoides* (2*n* = 44; Murray et al., 2005) and *Anemanthele lessoniana* (2*n* = 40–44; Dawson and Beuzenberg, 2000; Edgar and Connor, 2000). In some accessions a certain degree of aneusomaty was noted, for example, 2*n* = 65, 66, 68, 70 in *Austrostipa semibarbata*, but usually the chromosome number showed less variation or was uniform in the metaphase plates of each accession studied. *Austrostipa* and *Anemanthele* thus encompass consistently polyploids with a chromosome base number of *x* = 11. Apart from the overall similarity of their karyotypes, this common base number supports a close relationship of both genera and makes a common ancestry of *Austrostipa* and *Anemanthele* likely, in addition to the relationship shown by the molecular phylogenetic data (Figures 2, 3) (Jacobs et al., 2007; Romaschenko et al., 2012).

#### Monoploid Chromosome Number Variation in Stipeae

##### Clade A

*Austrostipa* and *Anemanthele* were placed in a clade, in which otherwise the chromosome base number of *x* = 12 prevails (Clade A in Figure 7). This supports recognizing *x* = 11 as a synapomorphic character of both genera in this clade. The base number of *x* = 12 was found in the likely sister of *Austrostipa* and *Anemanthele*, namely a lineage formed by *Achnatherum* (2*n* = 2*x* = 24; rarely 2*n* = 28 and few polyploids; see Supplementary Appendix 2) and *Oloptum* (usually 2*n* = 2*x* = 24), whereas *Stipellula* most likely deviates from *x* = 12. Various somatic chromosome numbers have been reported for *S. capensis* (2*n* = 18, ca. 34, 36; Supplementary Appendix 2), 2*n* = 36 being the most frequent in the whole Mediterranean (Supplementary Appendix 2). 2*n* = 18 appears to be trustworthy for an accession from Gran Canaria, Canary Islands (Borgen, 1970 using the synonym *Stipa retorta* Cav.), making a derived monoploid chromosome number of *x* = 9 strongly conceivable for this species with annual life form, which is unusual in Stipeae. Moreover, 2*n* = 28, possibly pointing to *x* = 7, was reported in its congener *Stipellula parviflora* (Desf.) Röser & Hamasha (Supplementary Appendix 2). The clade of *Austrostipa*, *Anemanthele*, *Achnatherum*, *Oloptum* and *Stipellula* has highly polyploid, monospecific *Celtica* (usually 2*n* = 8*x* = 96; *x* = 12) as sister. Australian/New Zealand *Austrostipa* and *Anemanthele* therefore are related to a group of genera distributed in Eurasia, the Mediterranean and with few outliers in Tropical East and South Africa (Clayton, 1970, 1972; Freitag, 1989; Fish et al., 2015).

##### Clade B

Sister to all these Clade A genera is an almost exclusively and comparatively large New World lineage (Clade B) with a wide range of chromosome numbers (Supplementary Appendix 2 and Figure 7). Chromosome numbers of *Eriocoma* (2*n* = 32, 34, 36, 40, 44, 48, 64, 66, 68, 70) and very speciose *Nassella* (2*n* = 26, 28, 30, 32, 34, 36, 38, 40, 42, 56, 58, 60, 64, 66, 70, 82, 88; Supplementary Appendix 2) seem to be based prevailingly on *x* = 11, implying the occurrence of 4*x*, 6*x*, 8*x* and possibly also 3*x* and 5*x* ploidy levels and a certain degree of aneusomatic variation. Assuming that chromosome numbers of 2*n* = 32–34 are triploid numbers based on *x* = 11, the occurrence of triploids and pentaploids points toward heteroploid diploid-tetraploid and tetraploid-hexaploid hybridizations.

Also lower monoploid numbers such as *x* = 6 suggested by Stebbins and Love (1941, p. 379) for *Eriocoma*, and *x* = 7, 8 suggested by Barkworth (2007) for *Nassella* or *x* = 9 might occur in both genera, which means that accessions with 2*n* = 26, 28, 32, 36, 38 would represent tetraploids or hexaploids. Given the branching order in the phylogenetic scheme of Figure 7, such hypothetical monoploid chromosome sets of *Ericoma* and *Nassella* with *x* = 7–9 have originated secondarily from *x* = 11, the most likely original number of Clade B. In this phylogenetic context they do not give evidence of a sometimes suggested low ‘original’ base chromosome number of Stipeae (see Stebbins and Love, 1941; Johnson, 1972; Tzvelev, 1977). Numbers reported in *Amelichloa* (2*n* = 40, 44, 46), *Jarava* (2*n* = 36, 40, 44, 66), and *Pseudoeriocoma* Romasch., P.M.Peterson & Soreng (2*n* = 44, 46) seem to be based most likely on *x* = 11 if aneusomaty also plays some role here to explain the slightly varying chromosome numbers (Figure 7). Chromosome numbers are unknown in monospecific North American *Thorneochloa* and in *Timouria*, a Central to East Asian outlier of this otherwise American clade. In summary, we suggest a secondary reduction of chromosome numbers in *Eriocoma* and *Nassella* via aneusomaty, whereas the chromosome base number originally was *x* = 11 in Clade B and not lower (Figure 7). This supposed reductional dysploidy in *Nassella* would agree with the result that the species of *Nassella* with low chromosomes numbers [2*n* = 26, 28, 30 in *N. leptocoronata* (Roseng. & B.R.Arrill.) Barkworth, *N. neesiana* (Trin. & Rupr.) Barkworth, *N. longiglumis* (Phil.) Barkworth; González et al., 2017; Supplementary Appendix 2] have comparatively large chromosomes due to non-reciprocal translocations from chromosomes that finally became lost.

##### Transcontinental Stipeae clade

Both lineages of prevailingly *x* = 12 (Clade A) and *x* = 11 (Clade B), though with exceptions in *Stipellula* and species of *Eriocoma* and *Nassella*, constitute one of the major clades in Stipeae, which was named ‘Transcontinental Stipeae clade’ in Hamasha et al. (2012) to denote its representation on all continents including Australia and New Zealand (Figure 7), and it is congruent with the ‘achnatheroid clade’ of Romaschenko et al. (2012).

##### Main *x* = 12 clade

The Transcontinental Stipeae clade is allied with further genera of prevailingly *x* = 12, altogether forming the Main *x* = 12 clade of Stipeae (Figure 7), namely comparatively species-rich and consistently diploid *Piptatherum* from the Mediterranean and Eurasia (32 species; 2*n* = 2*x* = 24; Supplementary Appendix 2), East Asian/North American tetraploid *Patis* (three species; 2*n* = 4*x* = 46, 48) and monospecific North American *Barkworthia*, in which 2*n* = 4*x* = 40 was reported, implying a monoploid set of *x* = 10 (Supplementary Appendix 2) (Myers, 1947 citing an unpublished count of G.L. Stebbins). The Main *x* = 12 clade was recovered also in the plastid DNA and morphological study of Cialdella et al. (2007) in American Stipeae and was termed ‘Clade 2 or Aneuploid clade.’

##### Main *x* = 11 clade

This clade represents the second main clade of Stipeae and includes *Stipa* s.str., by far the largest genus of this tribe (Figure 7). It agrees with the ‘Clade 1 or *x* = 11 clade’ of Cialdella et al. (2007). Exceptions from *x* = 11 are seemingly scarce in this clade but were noted for North American monospecific *Oryzopsis* (only *O. asperifolia* Michx. with probably *x* = 12), monotypic Asian *Neotrinia* (uncertain *x* = 11 or 12) and some species of the North American genus *Piptatheropsis* (five species; Supplementary Appendix 1). In *P. pungens* (Torr. ex Spreng.) Romasch., P.M.Peterson & Soreng 2*n* = 22 and 24 were found, the latter number possibly caused by aneusomaty, whereas 2*n* = 2*x* = 20 was counted in mitotic and meiotic stages of two different accessions in *P. shoshoneana* (Curto & Douglass M.Hend.) Romasch., P.M.Peterson & Soreng (Curto and Henderson, 1998), which implies *x* = 10, and represents the lowest chromosome number of Stipeae in the New World as noted already by these authors.

##### Clades C and D

The Main *x* = 11 clade is geographically clearly structured because it is divided into the Eurasian/Mediterranean Clade C (*Neotrinia*, *Orthoraphium*, *Psammochloa*, *Stipa*, *Trikeraia*) and the New World Clade D (*Aciachne* Benth., *Anatherostipa* (Hack. ex Kuntze) Peñail., *Hesperostipa* (M.K.Elias) Barkworth, *Lorenzochloa* Reeder & C.Reeder, *Ortachne*, *Pappostipa*, *Piptatheropsis*, *Ptilagrostiella* Romasch., P.M.Peterson & Soreng; Figure 7). There are only few exceptions since monospecific *Oryzopsis*, widely distributed in woodland of North America, is nested in Eurasian Clade C and *Ptilagrostis* occurs in mountainous to alpine landscapes of both Central Asia and western North America (Figure 7).

#### Stipeae Chromosome Base Number

The occurrence of two main clades in Stipeae, one clade primarily with *x* = 12 harboring also *Austrostipa* and *Anemanthele*, which are characterized by a derived number of *x* = 11, the other with primary *x* = 11 and few exceptions (see above *Stipellula*, *Oryzopsis*, species of *Piptatheropsis* and possibly also of *Eriocoma* and *Nassella*; Figure 7), raises the question which one was the ‘original’ chromosome base number of the whole tribe Stipeae. Due to the tree topology with monospecific *Macrochloa* Kunth as sister to the remainder of the tribe (Figure 7), this question cannot be reliably answered because in *Macrochloa* chromosome counts are equivocal, some suggesting *x* = 12 and others *x* = 11 or *x* = 10 (Supplementary Appendix 2). We regard *x* = 12, as firstly proposed by Avdulov (1931, p. 130) and accepted also by Romaschenko et al. (2012), a bit more probable as original chromosome base number of Stipeae than *x* = 11. Interestingly, this is supported mainly by chromosome numbers represented in the presumably closely related tribes of Stipeae (see below).

Further research into chromosome numbers of Stipeae, especially the re-examination of questionable counts contained in the older karyological literature as cited in reference works of Darlington and Wylie (1956) and Fedorov (1969), particularly the reported low numbers, seems worthwhile. This problem is frequently encountered with older chromosome counts also in other plants groups, because counting was made using tissue sections, in which single chromosomes could easily become lost, instead of the nowadays employed and more reliable squashing technique.

#### The Lowest Chromosome Number in Stipeae

2*n* = 18 counted in a therefore diploid accession of *Stipellula capensis* from the Canary Islands (Borgen, 1970) seems to represent the lowest reliably known chromosome number of the whole tribe Stipeae (Supplementary Appendix 2). The chromosome number so far considered as lowest in Stipeae (Curto and Henderson, 1998; Barkworth, 2007) refers to a Crimean accession of *Achnatherum bromoides* with likewise 2*n* = 18 (Petrova, 1968), which was cited also in the reference works of Prokudin et al. (1977) and Agapova et al. (1993). This report appears to be questionable in view of the other chromosome counts available for *A. bromoides*, namely 2*n* = 24 (Ghukasyan, 2004) and repeatedly reported 2*n* = 28 (Vázquez and Devesa, 1996 and references therein). *Stipellula capensis*, however, is otherwise known from many tetraploid populations widespread in the Mediterranean (2*n* = 36; Supplementary Appendix 2) and has further chromosome numbers (see above and Supplementary Appendix 2), which are currently difficult to interpret (possible triploid hybrids, aneusomatic specimens, and partly probably erroneous counting).

#### Neighbor Tribes of Stipeae Also Have *x* = 12

Presumably close relatives of grass tribe Stipeae, which likewise belong to the rather early diverging lineages of grass subfamily Pooideae (Schneider et al., 2009, 2011; Romaschenko et al., 2012; Saarela et al., 2015), seem to share the chromosome base number of *x* = 12 with Stipeae (see above), even though only comparatively few counts are available: (1) monospecific *Ampelodesmos* Link [*A. mauritanicus* (Poir.) T.Durand & Schinz], regarded as either sole member of tribe Ampelodesmeae (GPWG (Grass Phylogeny Working Group), 2001; Soreng et al., 2017) or as morphologically anomalous genus of Stipeae (Decker, 1964; Barkworth, 2007; Schneider et al., 2009, 2011; Winterfeld et al., 2015) has 2*n* = 4*x* = 48 (Nilsson and Lassen, 1971; Schneider et al., 2011) or 2*n* = 8*x* = 96 (Myers, 1947 citing an unpublished count of G.L. Stebbins); (2) *Danthoniastrum compactum* (Boiss. & Heldr.) Holub, *Duthiea brachypodium* (P.Candargy) Keng & Keng f., *Sinochasea trigyna* Keng, *Stephanachne monandra* (P.C.Kuo & S.L.Lu) P.C.Kuo & S.L.Lu and *S. pappophorea* (Hack.) Keng (all tribe Duthieeae) all have 2*n* = 2*x* = 24 (Fedorov, 1969, p. 565 citing an unpublished count of L.A. Alexandrova; Winterfeld, 2006; Schneider et al., 2011; Zhang et al., 2018; with a discussion of a seemingly wrong previous chromosome counts of 2*n* = 14 in *Danthoniastrum compactum* of Kožuharov and Petrova, 1991), while *n* = 14 in was reported for *Duthiea bromoides* Hack. (Mehra and Sharma, 1975, 1977); and (3) monospecific *Phaenosperma* Munro ex Benth. (*P. globosum* Munro ex Benth.) of monogeneric tribe Phaenospermateae has 2*n* = 2*x* = 24 (Avdulov, 1931, p. 92; Tateoka, 1954, 1955, 1956; Schneider et al., 2011; Winterfeld et al., 2015; Zhang et al., 2018).

#### Whole-Genome Duplications in Stipeae

Although chromosome numbers are still unknown for a number of small genera encompassing only eight species (see above and Supplementary Appendix 2), the enormous significance of whole-genome duplications (Johnson, 1972) is clearly obvious in many genera of Stipeae, for which chromosome numbers are available. Diploids are by far the minority in this tribe and only four of 33 genera (12%) are consistently diploid and two further genera (6%) have diploid as well as polyploid species. It was pointed out already by Tzvelev (1977) that most extant Stipeae are polyploids and have hybrid origin as corroborated by our exemplary findings on the single-copy gene *Acc1* in *Austrostipa* and *Stipa* (Figure 3). Although no correlation between whole-genome duplication and diversification could be found in many tested clades of angiosperms (Clark and Donoghue, 2018), most, if not all of the larger, speciose genera of Stipeae have consistently polyploid species as far as known, for example, *Stipa* (>150 species) as delineated in the present (*Stipa* s.str.), *Nassella* (117), *Austrostipa* (64), *Eriocoma* (27), *Pappostipa* (31), etc., taking into account that relative low chromosome numbers in some species of *Nassella* and *Eriocoma* might be derived from polyploids (see above).

### Biogeographic Relations and Origin of *Austrostipa* and *Anemanthele*

*Austrostipa* and *Anemanthele* were previously considered as overall rather derived members of Stipeae (ITS analyses of Jacobs et al., 2000, 2007), having groups of American Stipeae like *Amelichloa*, *Jarava*, *Nassella*, American *Achnatherum*, whose species have meanwhile been transferred to *Eriocoma* and *Pseudoeriocoma* (Peterson et al., 2019), and Eurasian *Achnatherum* as phylogenetically close relatives. On the other hand, considerable differences in lemma epidermal patterns both within *Austrostipa* and between some of these genera were noted. Definitely most *Austrostipa* species have maize-like LEP as widespread among achnatheroids, occasionally showing variants such as the prickly maize-like or patterns with particularly shaped fundamental cells (see above), whereas some of the American genera have further maize-like types, for example, in *Amelichloa, Eriocoma*, or *Pseudoeriocoma* or strongly deviant LEPs as in *Nassella* with ladder-like LEP (Barkworth and Everett, 1987; Romaschenko et al., 2010, 2012).

**FIGURE 8:**
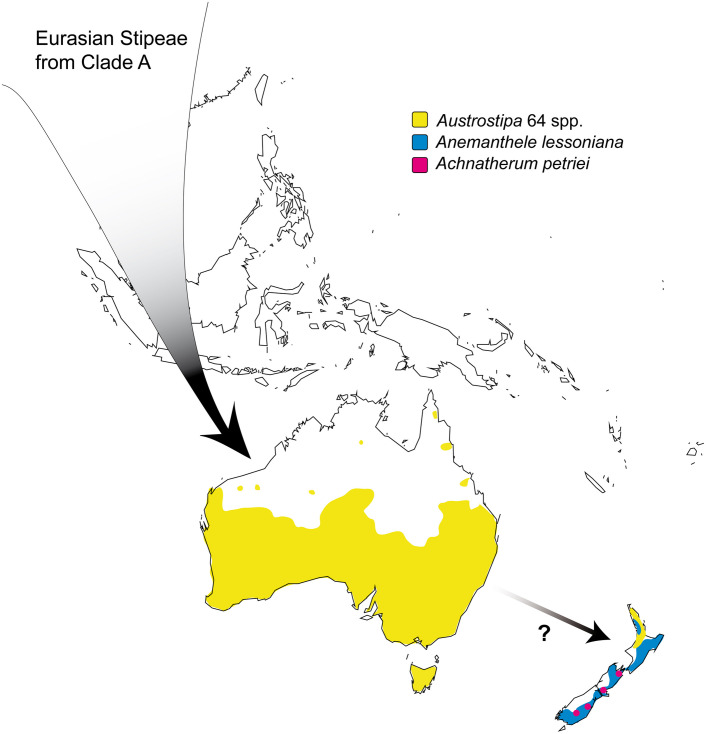
Simplified distributions ranges of *Austrostipa*, monogeneric *Anemanthele* (*A. lessoniana*; endemic New Zealand) and *Achnatherum petriei* (endemic New Zealand), the presumably sole autochthonous representative of this genus in Australasia. The arrows point to a hypothesized colonization of Australia/New Zealand by Central to East Asian ancestors belonging to Stipeae Clade A via long-distance dispersal. See text for comments on the phylogenetic relationships of the three genera and their distribution.

Considering *Austrostipa*, Tzvelev (1977) discussed a migration of Stipeae from South America via Antarctica to Australia as more likely than migration of Stipeae from north to south, i.e., from Eurasia to Australia. Nevertheless, he argued that the Australian feathergrasses were morphologically closer to Eurasian sections of *Stipa* than to sections of South American feathergrasses and cited among the examples also *Stipa* section *Stipella* Tzvelev which encompassed only *Stipa capensis* Thunb. (≡ *Stipellula capensis*) in his view (Tzvelev, 2011). Within the Transcontinental Stipeae clade, *Austrostipa* and *Anemanthele* are closely affiliated with Eurasian to Mediterranean genera, namely *Achnatherum* (including few eastern to South Africa species; see above), *Celtica*, *Oloptum* and also *Stipellula*, whereas they are distant to the American members of this clade (Figure 7). This implies that the ancestors of *Austrostipa* and *Anemanthele* with *x* = 11 came from the lineage with *x* = 12 (Clade A) and not the mainly American lineage with *x* = 11 (Clade B), and therefore acquired *x* = 11 in parallel to the American representatives of the Transcontinental Stipeae clade. The ancestors were most probably diploid like most if not all extant species of *Achnatherum* except for tetraploid South African *A. dregeanum*, comb. nov., with 2*n* = 48 (Supplementary Appendix 2). Whole-genome duplication could have followed later but probably preceded the evolutionary radiation of *Austrostipa*. Finally, the colonization of Australia started likely from Central/East Asia, where *Achnatherum* species, for example, are well represented in the present and the precursors of *Austrostipa* might have occurred as well. Judging from the current distribution, with no occurrences of *Achnatherum* in subtropical and tropical southeastern mainland Asia and Indonesia/New Guinea, long-distance dispersal from Central/East Asia to Australia as pictured in Figure 8 seems plausible. It has parallels considering, for example, the sister relation between *Duthiea* Hack. ex Procop.-Procop., a genus of China and the Himalayas, and *Anisopogon* from southeastern Australia in the neighboring tribe Duthieeae (plastid DNA data of Schneider et al., 2011).

New Zealand *Anemanthele* could have acquired and established its main apomorphic character, the occurrence of only a single stamen per floret, due to isolation and initial small population size (Veldkamp, 1985; Jacobs and Everett, 1996; Edgar and Connor, 2000). It bears morphological resemblance with *Achnatherum* rather than with *Austrostipa* as noted by Jacobs and Everett (1996, p. 582). It is not sure that the precursor of *Anemanthele* came from Australia as likely in *Austrostipa stipoides*, the only autochthonous species of this genus in New Zealand. *Achnatherum* is not represented in Australia by any autochthonous species, whereas New Zealand harbors an endemic species of *Achnatherum*, *A. petriei* (Figure 8) (Edgar and Connor, 2000), the presumably only autochthonous representative of this genus in whole Australasia. Chromosome counts reported 2*n* = 42 for this species (Supplementary Appendix 2), which is remarkable because otherwise 2*n* = 24 is characteristic of *Achnatherum* (Supplementary Appendix 2), seemingly except for *A. bromoides*, in which both 2*n* = 24 and 2*n* = 28 was reported (see above). The tetraploid number of *A. petriei* is most likely based on *x* = 11, if aneusomatic change from 44 to 42 is assumed, rather than on *x* = 12. *Achnatherum petriei* thus seems to share with *Austrostipa* and *Anemanthele* the chromosome base number of *x* = 11 representing most likely a synapomorphy within Clade A, and shares the ploidy level of 4*x*, both in marked contrast to typical *Achnatherum*. It can be assumed therefore that *Achnatherum petriei* might be a close relative of *Anemanthele* and *Austrostipa* pending further investigation.

## New Names and Combinations

***Achnatherum dregeanum*** (Steud.) Röser, Tkach & M.Nobis, **comb. nov.** ≡ *Stipa dregeana* Steud., Syn. Pl. Glumac. 1(2): 132. 1854.

*Note:* This South African species does not belong to *Stipa* as delineated by most current taxonomic treatments. It seems best to accommodate this species under *Achnatherum* pending further investigations. *Stipa dregeana* var. *dregeana* is considered as South African endemic (Fish et al., 2015), whereas Afro-montane var. *elongata* (Nees) Stapf (syn. *S. keniensis* (Pilg.) Freitag subsp. *keniensis*) occurs in Ethiopia and Tanzania (Freitag, 1989).

***Austrostipa*** subg. ***Paucispiculatae*** Röser, Tkach & M.Nobis, **subg. nov.** – Type: *A. muelleri* (Tate) S.W.L.Jacobs & J.Everett ≡ *Stipa muelleri* Tate in Trans. Proc. & Rep. Roy. Soc. South Australia 7: 70. 1885.

*Description*: Inflorescence reduced to 1–3 spikelets, lemma with maize-like epidermal pattern, with short dumbbell-shaped fundamental cells and frequent elongate to ovate silica cells, apex of lemma with two distinct, ca. 3 mm long lobes; spreading plants without basal tuft of leaves.

*Included species*: *A. muelleri.*

*Distribution*: Australia: SE South Australia and S Victoria.

*Etymology*: The epithet is derived from Latin ‘paucus’ (few) and ‘spicula, spiculatus’ (spikelet, with spikelets).

## Data Availability Statement

The datasets presented in this study can be found in online repositories. The names of the repository/repositories and accession number(s) can be found in the article/Supplementary Material.

## Author Contributions

MR, NT, SJ, and MN: research conception and design. SJ: field work. JS, NT, and HB: acquisition of molecular data and phylogenetic analysis. MN: acquisition and analysis of micromorphological and morphological data and scanning electron microscopy (SEM). MR, GW, and NT: acquisition and analysis of cytogenetic data. MR, NT, and MN: drafting of the manuscript. All authors: final critical revision.

## Conflict of Interest

The authors declare that the research was conducted in the absence of any commercial or financial relationships that could be construed as a potential conflict of interest.

## Footnotes

1 https://www.geneious.com

2 http://tree.bio.ed.ac.uk/software/figtree/

## Supplementary Material

Supplementary Figure 1 Maximum likelihood phylogram of all studied accessions of *Austrostipa* species inferred from plastid 3′*trnK* region DNA sequences with *Anisopogon avenaceus* (Duthieeae), *Bromus erectus* (Bromeae), *Hordeum vulgare*, and *Secale sylvestre* (both Triticeae) used as outgroup. ML and MP bootstrap support values ≥ 50% as well as Bayesian PP ≥ 0.5 are indicated on the branches. Clades with ML support < 50% are collapsed. The taxonomic groupings of the *Austrostipa* species according to Jacobs and Everett (1996) and this study are marked by different colors in columns 1 and 2. *A*., *Austrostipa*.

Supplementary Figure 2 Maximum likelihood phylogram of all studied accessions of *Austrostipa* species inferred from nuclear ribosomal ITS1–5.8S gene–ITS2 DNA sequences with *Anisopogon avenaceus* (Duthieeae), *Bromus erectus* (Bromeae), *Hordeum vulgare*, and *Secale sylvestre* (both Triticeae) used as outgroup. ML and MP bootstrap support values ≥ 50% as well as Bayesian PP ≥ 0.5 are indicated on the branches. Clades with ML support < 50% are collapsed. The taxonomic groupings of the *Austrostipa* species according to Jacobs and Everett (1996) and this study are marked by different colors in columns 1 and 2. *A*., *Austrostipa*.

Supplementary Figure 3 Cluster analysis (UPGMA) performed on four micromorphological characters of 34 *Austrostipa* taxa. See Supplementary Table 2 for the data matrix evaluated. The taxonomic groupings of the *Austrostipa* species according to Jacobs and Everett (1996) and this study are marked by different colors in columns 1 and 2. *A*., *Austrostipa*.

Supplementary Figure 4 Cluster analysis (UPGMA) performed on nine macromorphological characters of 65 *Austrostipa* taxa. See Supplementary Table 1 for the data matrix evaluated. The taxonomic groupings of the *Austrostipa* species according to Jacobs and Everett (1996) and this study are marked by different colors in columns 1 and 2. *A*., *Austrostipa*.

Supplementary Table 1 Data matrix used for macromorphological analysis. See Table 3 for measurements and character coding. *A., Austrostipa*.

Supplementary Table 2 Data matrix used for micromorphological and combined macro- and micromorphological analyses. See Table 3 for measurements and character coding. *A., Austrostipa*.

Supplementary Appendix 1 Taxa studied for DNA sequences, morphology and lemma micromorphology. Taxon, geographical origin, voucher information with collectors and herbarium code and ENA/GenBank accession numbers for plastid *matK* gene–3′*trnK* exon region; nuclear ribosomal ITS1–5.8S gene–ITS2 and nuclear single-copy gene *Acc1*. Sequences LR989057–LR989267 were newly generated for this study. A single asterisk (^∗^) indicates sequences previously generated in our lab, two asterisks (^∗∗^) indicate sequences taken from ENA/GenBank, a dash (–) missing data. The uppercase letters (A), (B) and (C) denote different *Acc1* sequence copies. BG, Botanical Garden; LEP, lemma epidermal pattern studied; LEP ill., LEP illustrated in Figures 4, 5; morph, morphological data for Supplementary Table 2 taken from Everett et al. (2009); MSB, Millennium Seed Bank, Kew, Wakehurst Place; NSW, New South Wales; NT, Northern Territory; SA, South Australia; TAS, Tasmania; VIC, Victoria; WA, Western Australia.

Supplementary Appendix 2 Annotated list of chromosome number reports for taxa of Stipeae. Underlined are the most frequent chromosome numbers in case of different counting, if this was stated in the original publications or if concluded from this survey (mostly for the genera). Original publications we did not examine are identified as such in the list of references below.

## References

[B1] Acedo C. Llamas F. (2001). Variation of micromorphological characters of lemma and palea in the genus *Bromus* (Poaceae). *Ann. Bot. Fenn.* 38 1–14. 10.25224/1097-993x-18.1.1

[B2] Agapova N. D. Arkharova K. B. Vakhtina E. A. Zemskova E. A. Tarvis L. V. (1993). *Chisla Khromosom Tsvetkovykh Rasteniy Flory SSSR: Moraceae–Zygophyllaceae [Chromosome Numbers in Flowering Plants of the Flora of the USSR: Moraceae–Zygophyllaceae].* St. Petersburg: Nauka.

[B3] Álvarez I. Wendel J. F. (2003). Ribosomal ITS sequences and plant phylogenetic inference. *Mol. Phylogenet. Evol.* 29 417–434. 10.1016/S1055-7903(03)00208-2 14615184

[B4] Avdulov N. (1931). Kario-sistematicheskoe issledovanie semeistva zlakov. [Karyosystematic study of the family of grasses.] *Trudy Prikl. Bot. Selekts. Suppl.* 44 1–428. 10.1017/cbo9780511525445.002

[B5] Bailey C. D. Carr T. G. Harris S. A. Hughes C. F. (2003). Characterization of angiosperm nrDNA: polymorphism, paralogy, and pseudogenes. *Mol. Phylogenet. Evol.* 29 435–455. 10.1016/j.ympev.2003.08.021 14615185

[B6] Baldwin B. G. Sanderson M. J. Porter J. M. Wojciechowski M. F. Campbell C. S. Donoghue M. J. (1995). The ITS region of nuclear ribosomal DNA: a valuable source of evidence on angiosperm phylogeny. *Ann. Missouri Bot. Gard.* 82 247–277. 10.2307/2399880

[B7] Barber J. C. Hames K. A. Cialdella A. M. Giussani L. M. Morrone O. (2009). Phylogenetic relationships of *Piptochaetium* Presl (Poaceae: Stipeae) and related genera as reconstructed from nuclear and chloroplast datasets. *Taxon* 58 375–380. 10.1002/tax.582005

[B8] Barkworth M. E. (1983). *Ptilagrostis* in North America and its relationship to other Stipeae. *Syst. Bot.* 8 395–419. 10.2307/2418359

[B9] Barkworth M. E. (1990). *Nassella* (Gramineae: Stipeae): revised interpretation and nomenclatural changes. *Taxon* 39 597–614. 10.2307/1223366

[B10] Barkworth M. E. (1993). North American Stipeae (Gramineae): taxonomic changes and other comments. *Phytologia* 74 1–25. 10.5962/bhl.part.2304 33311142

[B11] Barkworth M. E. (2007). “Stipeae Dumort.,” in *Flora of North America North of Mexico*, *Magnoliophyta: Commelinidae (in part): Poaceae*, Vol. 24 (part 1) eds Barkworth M. E. Capels K. M. Long S. Anderton L. K. Piep M. B. (New York, NY: Oxford University Press), 109–186.

[B12] Barkworth M. E. Arriaga M. O. Smith J. F. Jacobs S. W. L. Valdés-Reyna J. Bushman B. S. (2008). Molecules and morphology in South American Stipeae (Poaceae). *Syst. Bot.* 33 719–731. 10.1600/036364408786500235

[B13] Barkworth M. E. Everett J. (1987). “Evolution in the Stipeae: identification and relationships of its monophyletic taxa,” in *Grass Systematics and Evolution*, eds Soderstrom T. R. Hilu K. W. Campbell C. S. Barkworth M. E. (Washington, DC: Smithsonian Institution Press), 251–264.

[B14] Barkworth M. E. Torres M. A. (2001). Distribution and diagnostic characters of *Nassella* (Poaceae: Stipeae). *Taxon* 50 439–468. 10.2307/1223891

[B15] Bayly M. J. Ladiges P. Y. (2007). Divergent paralogues of ribosomal DNA in eucalypts (Myrtaceae). *Mol. Phylogenet. Evol.* 44 346–356. 10.1016/j.ympev.2006.10.027 17188000

[B16] Blaner A. Schneider J. Röser M. (2014). Phylogenetic relationships in the grass family (Poaceae) based on the nuclear single copy locus topoisomerase 6 compared to chloroplast DNA. *Syst. Biodivers.* 12 111–124. 10.1080/14772000.2014.890137

[B17] Blattner F. R. (1999). Direct amplification of the entire ITS region from poorly preserved plant material using recombinant PCR. *BioTechniques* 27 1180–1186. 10.2144/99276st04 10631497

[B18] Borgen L. (1970). Chromosome numbers of Macaronesian flowering plants. *Nytt Mag. Bot.* 17 145–161.

[B19] Brassac J. Jakob S. S. Blattner F. R. (2012). Progenitor-derivative relationships of *Hordeum* polyploids (Poaceae, Triticeae) inferred from sequences of TOPO6, a nuclear low-copy gene region. *PLoS One* 7:e333808. 10.1371/journal.pone.0033808 22479447 PMC3316500

[B20] Buckler E. S. Ippolito A. Holtsford T. P. (1997). The evolution of ribosomal DNA: divergent paralogues and phylogenetic implications. *Genetics* 145 821–832. 9055091 10.1093/genetics/145.3.821 PMC1207866

[B21] Bustam B. (2012). *Systematic Studies of* Austrostipa *(Australian Stipoid Grasses): Comparison and Combined Analyses of Non-molecular and Molecular Data.* Saarbrücken: Lambert Academic Publishing.

[B22] Bustam B. M. (2010). Systematic studies of Australian stipoid grasses (*Austrostipa*) based on micro-morphological and molecular characteristics. *Biodiversitas* 11 9–14. 10.13057/biodiv/d110103

[B23] Catalán P. Kellogg E. A. Olmstead R. G. (1997). Phylogeny of Poaceae subfamily Pooideae based on chloroplast *ndh*F gene sequences. *Mol. Phylogenet. Evol.* 8 150–166. 10.1006/mpev.1997.0416 9299221

[B24] Chase M. W. Hills H. H. (1991). Silica gel: an ideal material for field preservation of leaf samples for DNA studies. *Taxon* 40 215–220. 10.2307/1222975

[B25] Cialdella A. M. Guissani L. M. Aagesen L. Zuloaga F. O. Morrone O. (2007). A phylogeny of *Piptochaetium* (Poaceae: Pooideae: Stipeae) and related genera based on a combined analysis including *trnL–F, rpl16*, and morphology. *Syst. Bot*. 32 545–559. 10.1600/036364407782250607

[B26] Cialdella A. M. Salariato D. L. Aagesen L. Giussani L. M. Zuloaga F. O. Morrone O. (2010). Phylogeny of New World Stipeae (Poaceae): an evaluation of the monophyly of *Aciachne* and *Amelichloa*. *Cladistics* 26 563–578. 10.1111/j.1096-0031.2010.00310.x 34879600

[B27] Cialdella A. M. Sede S. M. Romaschenko K. Peterson P. M. Soreng R. J. Zuloaga F. O. (2014). Phylogeny of *Nassella* (Stipeae, Pooideae, Poaceae) based on analyses of chloroplast and nuclear ribosomal DNA and morphology. *Syst. Bot.* 39 814–828. 10.1600/036364414X681419

[B28] Clark J. W. Donoghue P. C. J. (2018). Whole-genome duplication and plant macroevolution. *Trends Plant Sci.* 23 933–945. 10.1016/j.tplants.2018.07.006 30122372

[B29] Clayton W. D. (1970). “Gramineae,” in *Flora of Tropical East Africa*, (part 1) eds Milne-Redhead E. Polhill R. M. (London: Crown Agents for Oversea Governments and Administrations), 1–176.

[B30] Clayton W. D. (1972). “Gramineae,” in *Flora of West Tropical Africa*, Vol. 3 ed. Hepper F. N. (London: Crown Agents for Oversea Governments and Administrations), 349–512.

[B31] Curto M. Henderson D. M. (1998). A new *Stipa* (Poaceae: Stipeae) from Idaho and Nevada. *Madroño* 45 57–63.

[B32] Darlington C. D. Wylie A. P. (1956). *Chromosome Atlas of Flowering Plants.* London: George Allen and Unwin Ltd.

[B33] Davis J. I. Soreng R. J. (2007). A preliminary phylogenetic analysis of the grass subfamily Pooideae subfamily Pooideae (Poaceae), with attention to structural features of the plastid and nuclear genomes, including an intron loss in GBSSI. *Aliso* 23 335–348. 10.5642/aliso.20072301.27

[B34] Dawson M. I. Beuzenberg E. J. (2000). Contributions to a chromosome atlas of the New Zealand flora. *N. Z. J. Bot.* 38 1–23. 10.1080/0028825X.2000.9512671

[B35] Decker H. F. (1964). Affinities of the grass genus *Ampelodesmos*. *Brittonia* 16 76–79. 10.2307/2805186

[B36] Döring E. Schneider J. Hilu K. W. Röser M. (2007). Phylogenetic relationships in the Aveneae/Poeae complex (Pooideae, Poaceae). *Kew Bull.* 62 407–424.

[B37] Edgar E. Connor H. E. (2000). *Flora of New Zealand*, *Gramineae*, Vol. 5 Lincoln: Manaaki Whenua Press.

[B38] Everett J. Jacobs S. W. L. (1990). Notes on *Stipa* (Poaceae) in Australia and Easter Island. *Telopea* 4 7–11. 10.7751/telopea19904912

[B39] Everett J. Jacobs S. W. L. Nairn L. (2009). “Tribe Stipeae,” in *Flora of Australia*, *Poaceae 2*, Vol. 44A ed. Wilson A. (Melbourne, VIC: CSIRO Publishing), 11–70.

[B40] Fan X. Sha L.-N. Yang R.-W. Zhang H.-Q. Kang H.-Y. Ding C.-B. (2009). Phylogeny and evolutionary history of *Leymus* (Triticeae; Poaceae) based on a single-copy nuclear gene encoding plastid acetyl-CoA carboxylase. *BMC Evol. Biol.* 9:247. 10.1186/1471-2148-9-24 19814813 PMC2770499

[B41] Fan X. Zhang H. Sha L. Zhang L. Yang R. Ding C. (2007). Phylogenetic analysis among *Hystrix*, *Leymus* and its affinitive genera (Poaceae: Triticeae) based on the sequences of a gene encoding plastid acetyl-CoA carboxylase. *Plant Sci.* 172 701–707. 10.1016/j.plantsci.2006.11.012

[B42] Fedorov A. A. (1969). *Chromosome Numbers of Flowering Plants.* Leningrad: Izdatelstvo Nauka.

[B43] Fish L. Mashau A. C. Moeaha M. J. Nembudani M. T. (2015). *Identification Guide to Southern African Grasses. An Identification Manual With Keys, Descriptions and Distributions. Strelitzia* 36 Pretoria: South African National Biodiversity Institute.

[B44] Folk R. A. Mandel J. R. Freudenstein J. V. (2017). Ancestral gene flow and parallel organellar genome capture result in extreme phylogenomic discord in a lineage of angiosperms. *Syst. Biol.* 66 320–337. 27637567 10.1093/sysbio/syw083

[B45] Freitag H. (1975). The genus *Piptatherum* (Gramineae) in southwest and south Asia. *Notes R. Bot. Gard. Edinburgh* 33 341–408.

[B46] Freitag H. (1985). The genus *Stipa* (Gramineae) in southwest and south Asia. *Notes R. Bot. Gard. Edinburgh* 42 355–489.

[B47] Freitag H. (1989). “*Piptatherum* and *Stipa* (Gramineae) in the Arabian Peninsula and tropical East Africa,” in *The Davis and Hedge Festschrift*, ed. Tan K. (Edinburgh: Edinburgh University Press), 115–132.

[B48] Ghukasyan A. G. (2004). Kariologisheskaya izuchennost zlakov (Poaceae) Armenii. [Extent of karyological study of Armenian grasses (Poaceae).]. *Fl. Rastitel nost Rastitel’nye Resursy Armenii Flora Veg. plant Resour. Armen.* 15 74–84.

[B49] González A. C. Vaio M. Porro V. Folle G. Mazzella C. (2017). Chromosome numbers, DNA content, morphological data, and nrITS sequence analyses in some species of Nassella (Trin.) E. Desv. and related genera (Stipeae, Poaceae). *Brazil. J. Bot.* 40 341–352. 10.1007/s40415-016-0337-0

[B50] GPWG (Grass Phylogeny Working Group) (2001). Phylogeny and subfamilial classification of the grasses (Poaceae). *Ann. Missouri Bot. Gard.* 88 373–457. 10.2307/3298585

[B51] Hamasha H. von Hagen K. B. Röser M. (2012). *Stipa* (Poaceae) and allies in the old world: molecular phylogenetics realigns genus circumscription and gives evidence on the origin of American and Australian lineages. *Plant Syst. Evol.* 298 351–367. 10.1007/s00606-011-0549-5

[B52] Hammer O. Harper D. A. T. Ryan P. D. (2001). PAST: paleontological statistic software package for education and data analysis. *Palaeontol*. *Electron.* 4 1–9.

[B53] Hand M. L. Cogan N. O. Stewart A. V. Forster J. W. (2010). Evolutionary history of tall fescue morphotypes inferred from molecular phylogenetics of the *Lolium*-*Festuca* species complex. *BMC Evol. Biol.* 10:303. 10.1186/1471-2148-10-303 20937141 PMC2958922

[B54] Hilu K. W. Alice L. A. Liang H. (1999). Phylogeny of the Poaceae inferred from *mat*K sequences. *Ann. Missouri Bot. Gard*. 86 835–851. 10.2307/2666171

[B55] Hochbach A. Linder P. H. Röser M. (2018). Nuclear genes, *mat*K and the phylogeny of the Poales. *Taxon* 67 521–536. 10.12705/673.5

[B56] Hochbach A. Schneider J. Röser M. (2015). A multi-locus analysis of phylogenetic relationships within grass subfamily Pooideae (Poaceae) inferred from sequences of nuclear single copy gene regions compared with plastid DNA. *Mol. Phylogenet. Evol.* 87 14–27. 10.1016/j.ympev.2015.03.010 25804934

[B57] Hsiao C. Jacobs S. W. L. Chatterton N. J. Asay K. H. (1999). A molecular phylogeny of the grass family (Poaceae) based on the sequences of nuclear ribosomal DNA (ITS). *Aust. Syst. Bot.* 11 667–688. 10.1071/SB97012

[B58] Huang S. Sirikhachornkit A. Su X. Faris J. Gill B. Haselkorn R. (2002). Genes encoding plastid acetyl-CoA carboxylase and 3-phosphoglycerate kinase of the *Triticum*/*Aegilops* complex and the evolutionary history of polyploid wheat. *Proc. Natl. Acad. Sci. U.S.A.* 99 8133–8138. 10.1073/pnas.072223799 12060759 PMC123033

[B59] Jacobs S. Bayer R. Everett J. Arriaga M. Barkworth M. Sabin-Badereau A. (2007). Systematics of the tribe Stipeae (Gramineae) using molecular data. *Aliso* 23 349–361. 10.5642/aliso.20072301.28

[B60] Jacobs S. W. L. Everett J. (1996). *Austrostipa*, a new genus, and new names for Australasian species formerly included in *Stipa* (Gramineae). *Telopea* 6 579–595. 10.7751/telopea19963026

[B61] Jacobs S. W. L. Everett J. Barkworth M. E. Hsiao C. (2000). “Relationships within the stipoid grasses (Gramineae),” in *Grasses, Systematics and Evolution*, eds Jacobs S. W. L. Everett J. (Melbourne, VIC: CSIRO Publishing), 75–82.

[B62] Jacobs S. W. L. Everett J. Connor H. E. Edgar E. (1989). Stipoid grasses in New Zealand. *N. Z. J. Bot.* 27 569–582. 10.1080/0028825X.1989.10414140

[B63] Johnson B. L. (1972). “Polyploidy as a factor in the evolution and distribution of grasses,” in *The Biology and Utilization of Grasses*, eds Younger V. B. McKell C. M. (New York, NY: Academic Press), 18–35. 10.1016/B978-0-12-774750-7.50008-7

[B64] Johnson L. A. Soltis D. E. (1994). *matK* DNA sequences and phylogenetic reconstruction in Saxifragaceae s.str. *Syst. Bot.* 19 143–156. 10.2307/2419718

[B65] Kawabe A. Nukii H. Furihata H. Y. (2018). Exploring the history of chloroplast capture in *Arabis* using whole chloroplast genome sequencing. *Int. J. Mol. Sci.* 19:602. 10.3390/ijms19020602 29463014 PMC5855824

[B66] Kearse M. Moir R. Wilson A. Stones-Havas S. Cheung M. Sturrock S. (2012). Geneious basic: an integrated and extendable desktop software platform for the organization and analysis of sequence data. *Bioinformatics* 28 1647–1649. 10.1093/bioinformatics/bts199 22543367 PMC3371832

[B67] Kellogg E. A. (2015). “The families and genera of vascular plants,” in *Flowering Plants: Monocots* Vol. 13 Poaceae. Cham: Springer.

[B68] Kožuharov S. I. Petrova A. V. (1991). Chromosome numbers of Bulgarian angiosperms. *Fitologija* 39 72–77.

[B69] Krawczyk K. Nobis M. Myszczyński K. Klichowska E. Sawicki J. (2018). Plastid super-barcodes as a tool for species discrimination in feather grasses (Poaceae: *Stipa*). *Sci. Rep.* 8:1924. 10.1038/s41598-018-20399-w 29386579 PMC5792575

[B70] Krawczyk K. Nobis M. Nowak A. Szczecińska M. Sawicki J. (2017). Phylogenetic implications of nuclear rRNA IGS variation in *Stipa* L. (Poaceae). *Sci. Rep.* 7:11506. 10.1038/s41598-017-11804-x 28912548 PMC5599551

[B71] Larkin M. A. Blackshields G. Brown N. P. Chenna R. McGettigan P. A. McWilliam H. (2007). ClustalW and ClustalX version 2.0. *Bioinformatics* 23 2947–2948. 10.1093/bioinformatics/btm404 17846036

[B72] Liang H. Hilu K. W. (1996). Application of the *matK* gene sequences to grass systematics. *Can. J. Bot.* 74 125–134. 10.1139/b96-017

[B73] Mathews S. Tsai R. C. Kellogg E. A. (2000). Phylogenetic structure in the grass family (Poaceae): evidence from the nuclear gene phytochrome B. *Am. J. Bot.* 87 96–107. 10.2307/2656688 10636833

[B74] Mehra P. N. Sharma M. L. (1975). IOPB chromosome number reports XLIX. *Taxon* 24 501–516. 10.1002/j.1996-8175.1975.tb00341.x

[B75] Mehra P. N. Sharma M. L. (1977). Cytological studies on some grasses of Kashmir. *Cytologia* 42 111–123. 10.1508/cytologia.42.111

[B76] Mejía Saulés T. Bisby F. A. (2003). Silica bodies and hooked papillae in lemmas of *Melica* species (Gramineae: Pooideae). *Bot. J. Linn. Soc.* 141 447–463. 10.1046/j.1095-8339.2003.00152.x

[B77] Murray B. G. de Lange P. J. Ferguson A. R. (2005). Nuclear DNA variation chromosome numbers and polyploidy in the endemic and indigenous grass flora of New Zealand. *Ann. Bot. (Oxford)* 96 1293–1305. 10.1093/aob/mci281 16243852 PMC4247080

[B78] Myers W. M. (1947). Cytology and genetics of forage grasses. *Bot. Rev. (Lancaster)* 13 319–422. 10.1007/bf02861547

[B79] Nieto Feliner G. Rosselló J. A. (2007). Better the devil you know? Guidelines for insightful utilization of nrDNA ITS in species-level evolutionary studies in plants. *Mol. Phylogenet. Evol.* 44 911–919. 10.1016/j.ympev.2007.01.013 17383902

[B80] Nilsson Ö. Lassen P. (1971). Chromosome numbers of vascular plants from Austria, Mallorca and Yugoslavia. *Bot. Not*. 124 270–276.

[B81] Nobis M. (2013). Taxonomic revision of the *Stipa lipskyi* group (Poaceae: *Stipa* section *Smirnovia*) in the Pamir Alai and Tian-Shan Mountains. *Plant. Syst. Evol.* 299 1307–1354. 10.1007/s00606-013-0799-5

[B82] Nobis M. Gudkova P. D. Baiakhmetov E. Żabicka J. Krawczyk K. Sawicki J. (2019a). Hybridisation, introgression events and cryptic speciation in *Stipa* (Poaceae): a case study of the *Stipa heptapotamica* hybrid-complex. *Perspect. Plant Ecol. Evol. Syst.* 39:125457 10.1016/j.ppees.2019.05.001

[B83] Nobis M. Gudkova P. D. Nowak A. (2019b). *Neotrinia* gen. nov. and *Pennatherum* sect. nov. in *Achnatherum* (Poaceae: Stipeae). *Turczaninowia* 22 37–41. 10.14258/turczaninowia.22.1.5

[B84] Nobis M. Gudkova P. D. Nowak A. Sawicki J. Nobis A. (2020). A synopsis of the genus *Stipa* (Poaceae) in Middle Asia, including a key to species identification, an annotated checklist, and phytogeographic analyses. *Ann. Missouri Bot. Gard.* 105 1–63. 10.3417/2019378 2019378

[B85] Nobis M. Gudkova P. D. Pendry C. (2019c). Synopsis of the tribe Stipeae (Poaceae) in Nepal. *PhytoKeys* 128 97–119. 10.3897/phytokeys.128.34637 31523155 PMC6711933

[B86] Olonova M. V. Barkworth M. E. Gudkova P. D. (2016). Lemma micromorphology and the systematics of Siberian species of *Stipa* (Poaceae). *Nordic J. Bot.* 34 322–334. 10.1111/njb.00881

[B87] Ortúñez E. de la Fuente V. (2010). Epidermal micromorphology of the genus *Festuca* L. (Poaceae) in the Iberian Peninsula. *Plant Syst. Evol.* 284 201–218. 10.1007/s00606-009-0248-7

[B88] Peñailillo P. (2002). El género *Jarava* Ruiz et Pav. (Stipeae-Poaceae): delimitación y nuevas combinaciones. *Gayana Bot.* 59 27–34.

[B89] Peñailillo P. (2003). “*Jarava* Ruiz et Pav.,” in *Catalogue of New World grasses (Poaceae): IV. subfamily Pooideae*, *Contr. U.S. Natl. Herb*, Vol. 48 eds Soreng R. J. Peterson P. M. Davidse G. Judziewicz E. J. Zuloaga F. O. Filgueiras T. S. (Washington, D.C: Smithsonian Institution), 402–409.

[B90] Peterson P. M. Romaschenko K. Soreng R. J. Valdés Reyna J. (2019). A key to the North American genera of Stipeae (Poaceae, Pooideae) with descriptions and taxonomic names for species of *Eriocoma*, *Neotrinia*, *Oloptum*, and five new genera: *Barkworthia*, ×*Eriosella*, *Pseudoeriocoma*, *Ptilagrostiella*, and *Thorneochloa*. *PhytoKeys* 126 89–125. 10.3897/phytokeys.126.34096 31360096 PMC6650443

[B91] Petrova O. A. (1968). “Khromosomnyy sostav nekotorykh zlakov flory Ukrainy v svyasi s usloviami ikh proizrastaniya. [Chromosomal composition of some Ukrainian grasses according to their growing conditions.],” in *Biologicheskaya Nauka v Universitetakh I Pedagogicheskikh Institutakh Ukrainy za 50 let. [Biological Science in Universities and Pedagogical Institutes of Ukraine for 50 years]*, ed. Nikitin V. N. (Charkov: Charkov University), 37–39.

[B92] Prokudin Yu. N Vovk A. G. Petrova O. A. Ermolenko E. D. Vernichenko Yu. V. (eds.) (1977). *Zlaki Ukrainy. [Grasses of Ukraine.].* Kiev: Naukova Dumka.

[B93] Razafimandimbison S. G. Kellogg E. A. Bremer B. (2004). Recent origin and phylogenetic utility of divergent ITS putative pseudogenes: a case study from the Naucleeae (Rubiaceae). *Syst. Biol.* 53 177–192. 10.1080/10635150490423278 15205048

[B94] Rieseberg L. H. Soltis D. E. (1991). Phylogenetic consequences of cytoplasmic gene flow in plants. *Evol. Trends Plant* 5 65–84.

[B95] Romaschenko K. Garcia-Jacas N. Peterson P. M. Soreng R. J. Vilatersana R. Susanna A. (2014). Miocene–Pliocene speciation, introgression, and migration of *Patis* and *Ptilagrostis* (Poaceae: Stipeae). *Mol. Phylogenet. Evol.* 70 244–259. 10.1016/j.ympev.2013.09.018 24096057

[B96] Romaschenko K. Peterson P. M. Soreng R. J. Futorna O. Susanna A. (2011). Phylogenetics of *Piptatherum* s.l. (Poaceae: Stipeae): evidence for a new genus, *Piptatheropsis*, and resurrection of *Patis*. *Taxon* 60 1703–1716. 10.1002/tax.606015

[B97] Romaschenko K. Peterson P. M. Soreng R. J. Garcia-Jacas N. Futoma O. Susanna A. (2008). Molecular phylogenetic analysis of the American Stipeae (Poaceae) resolves *Jarava* sensu lato polyphyletic: evidence for a new genus, *Pappostipa*. *J. Bot. Res. Inst. Texas* 2 165–192.

[B98] Romaschenko K. Peterson P. M. Soreng R. J. Garcia-Jacas N. Futorna O. Susanna A. (2012). Systematics and evolution of the needle grasses (Poaceae: Pooideae: Stipeae) based on analysis of multiple chloroplast loci, ITS, and lemma micromorphology. *Taxon* 61 18–44. 10.1002/tax.611002

[B99] Romaschenko K. Peterson P. M. Soreng R. J. Garcia-Jacas N. Susanna A. (2010). “Phylogenetics of Stipeae (Poaceae, Pooideae) based on plastid and nuclear DNA sequences,” in *Diversity, Phylogeny, and Evolution in the Monocotyledons*, eds Seberg O. Petersen G. Barfod A. F. Davis J. I. (Aarhus: Aarhus University Press), 511–537.

[B100] Saarela J. M. Burke S. V. Wysocki W. P. Barrett M. D. Clark L. G. Craine J. M. (2018). A 250 plastome phylogeny of the grass family (Poaceae): topological support under different data partitions. *PeerJ* 6:e4299. 10.7717/peerj.4299 29416954 PMC5798404

[B101] Saarela J. M. Wysocki W. P. Barrett C. F. Soreng R. J. Davis J. I. Clark L. G. (2015). Plastid phylogenomics of the coolseason grass subfamily: clarification of relationships among early-diverging tribes. *AoB Plants* 7:plv046. 10.1093/aobpla/plv046 25940204 PMC4480051

[B102] Schneider J. Döring E. Hilu K. W. Röser M. (2009). Phylogenetic structure of the grass subfamily Pooideae based on comparison of plastid *matK* gene–3’*trnK* exon and nuclear ITS sequences. *Taxon* 58 405–424. 10.1002/tax.582008

[B103] Schneider J. Winterfeld G. Hoffmann M. H. Röser M. (2011). Duthieeae, a new tribe of grasses (Poaceae) identified among the early diverging lineages of subfamily Pooideae: molecular phylogenetics, morphological delineation, cytogenetics, and biogeography. *Syst. Biodivers.* 9 27–44. 10.1080/14772000.2010.544339

[B104] Schneider J. Winterfeld G. Röser M. (2012). Polyphyly of the grass tribe Hainardieae (Poaceae: Pooideae): identification of its different lineages based on molecular phylogenetics, including morphological and cytogenetic characteristics. *Organ. Divers. Evol.* 12 113–132. 10.1007/s13127-012-0077-3

[B105] Sclovich S. E. Giussani L. M. Cialdella A. M. Sede S. M. (2015). Phylogenetic analysis of *Jarava* (Poaceae, Pooideae, Stipeae) and related genera: testing the value of the awn indumentum in the circumscription of *Jarava*. *Plant Syst. Evol.* 301 1625–1641. 10.1007/s00606-014-1175-9

[B106] Seehausen O. (2004). Hybridization and adaptive radiation. *Trends Ecol. Evol.* 19 198–207. 10.1016/j.tree.2004.01.003 16701254

[B107] Sha L. Fan X. Yang R. Kang H. Ding C. Zhang L. (2010). Phylogenetic relationships between *Hystrix* and its closely related genera (Triticeae; Poaceae) based on nuclear *Acc1*, *DMC1* and chloroplast *trnL–F* sequences. *Mol. Phylogenet. Evol.* 54 327–335. 10.1016/j.ympev.2009.05.005 19435606

[B108] Snow N. (1996). The phylogenetic utility of lemmatal micromorphology in *Leptochloa* s.l. and related genera in subtribe Eleusininae (Poaceae, Chloridoideae, Eragrostideae). *Ann. Missouri Bot. Gard.* 83 504–529. 10.2307/2399991

[B109] Sokal R. R. Sneath P. H. (1963). *Principles of Numerical Taxonomy.* San Francisco, CA: W.H. Freeman.

[B110] Soreng R. J. Davis J. I. (2000). “Phylogenetic structure in Poaceae subfamily Pooideae as inferred from molecular and morphological characters: misclassification versus reticulation,” in *Grasses: Systematics and Evolution*, eds Jacobs S. W. L. Everett J. (Melbourne, VIC: CSIRO Publishing), 61–74.

[B111] Soreng R. J. Davis J. I. Voionmaa M. A. (2007). A phylogenetic analysis of Poaceae tribe Poeae sensu lato based on morphological characters and sequence data from three plastid-encoded genes: evidence for reticulation, and a new classification of the tribe. *Kew Bull.* 62 425–454.

[B112] Soreng R. J. Peterson P. M. Davidse G. Judziewicz E. J. Zuloaga F. O. Filgueiras T. S. (2003). Catalogue of New World grasses (Poaceae): IV. subfamily Pooideae. *Contr. U.S. Natl. Herb.* 48 1–730.

[B113] Soreng R. J. Peterson P. M. Romaschenko K. Davidse G. Teisher J. K. Clark L. G. (2017). A worldwide phylogenetic classification of the Poaceae (Gramineae) II: an update and a comparison of two 2015 classifications. *J. Syst. Evol.* 55 259–290. 10.1111/jse.12262

[B114] Stebbins G. L. Love R. M. (1941). A cytological study of California forage grasses. *Am. J. Bot.* 28 371–382. 10.1002/j.1537-2197.1941.tb07983.x

[B115] Štorchová H. Hrdličková R. Chrtek J. Tetera M. Fitze D. Fehrer J. (2000). An improved method of DNA isolation from plants collected in the field and conserved in saturated NaCl/CTAB solution. *Taxon* 49 79–84. 10.2307/1223934

[B116] Syme A. E. (2011). Diversification rates in the Australasian endemic grass *Austrostipa*: 15 million years of constant evolution. *Plant Syst. Evol.* 298 221–227. 10.1007/s00606-011-0539-7

[B117] Syme A. E. Murphy D. J. Holmes G. D. Gardner S. Fowler R. Cantrill D. J. (2012). An expanded phylogenetic analysis of *Austrostipa* (Poaceae: Stipeae) to test infrageneric relationships. *Aust. Syst. Bot*. 25 1–10. 10.1071/SB10049

[B118] Tateoka T. (1954). Karyotaxonomic studies in Poaceae, II. *Rep. Annu. Natl. Inst. Genet.* 5 68–69.

[B119] Tateoka T. (1955). Karyotaxonomy in Poaceae III. Further studies of somatic chromosomes. *Cytologia* 20 296–306. 10.1508/cytologia.20.296

[B120] Tateoka T. (1956). Notes on some grasses I. *Bot. Mag. (Tokyo)* 69 311–315. 10.15281/jplantres1887.69.311

[B121] Terrell E. E. Peterson P. M. Wergin W. P. (2001). Epidermal features and spikelet micromorphology in *Oryza* and related genera (Poaceae: Oryzeae). *Smithsonian Contr. Bot.* 91 1–50. 10.5479/si.0081024X.91

[B122] Terrell E. E. Wergin W. P. (1981). Epidermal features and silica deposition in lemmas and awns of *Zizania* (Gramineae). *Am. J. Bot.* 68 697–707. 10.1002/j.1537-2197.1981.tb12402.x

[B123] Thomasson J. R. (1978). Epidermal patterns of the lemma in some fossil and living grasses and their phylogenetic significance. *Science* 199 975–977. 10.1126/science.199.4332.975 17752369

[B124] Thomasson J. R. (1981). Micromorphology of the lemma in *Stipa robusta* and *Stipa viridula* (Gramineae: Stipeae): taxonomic significances. *S. W. Naturalist* 26 211–214. 10.2307/3671126

[B125] Thomasson J. R. (1986). Lemma epidermal features in the North American species of *Melica*, and selected species of *Briza*, *Catabrosa*, *Glyceria*, *Neostapfia*, *Pleuropogon* and *Schizachne* (Gramineae). *Syst. Bot.* 11 253–262. 10.2307/2419112

[B126] Tkach N. Röser M. Suchan T. Cieślak E. Schönswetter P. Ronikier M. (2019). Contrasting evolutionary origins of two mountain endemics: *Saxifraga wahlenbergii* (Western Carpathians) and *S. styriaca* (Eastern Alps). *BMC Evol. Biol.* 19:18. 10.1186/s12862-019-1355-x 30634910 PMC6329101

[B127] Tkach N. Schneider J. Döring E. Wölk A. Hochbach A. Nissen J. (2020). Phylogenetic lineages and the role of hybridization as driving force of evolution in grass supertribe Poodae. *Taxon* 69 234–277. 10.1002/tax.12204

[B128] Tzvelev N. N. (1976). *Zlaki SSSR. [Grasses of the Soviet Union.]*. Leningrad: Nauka.

[B129] Tzvelev N. N. (1977). “O proiskhozhdenii i evolutsii kovyley (*Stipa* L.). [On the origin and evolution of feathergrasses (*Stipa* L.).],” in *Problemy Ekologii, Geobotaniki, Botanicheskoi Geografii i Floristiki*, eds Lebedev D. V. Karamysheva Z. V. (Leningrad: Academiya Nauk SSSR), 139–150.

[B130] Tzvelev N. N. (2011). Zametki o tribe kovylevykh (Stipeae Dumort., Poaceae). [Notes on the tribe Stipeae Dumort. (Poaceae).]. *Novosti Sist. Vyssh. Rast.* 43 20–29.

[B131] Valdés-Reyna J. Hatch S. L. (1991). Lemma micromorphology in the Eragrostideae (Poaceae). *Sida* 14 531–549.

[B132] Vázquez F. M. Devesa J. A. (1996). Revisión del género *Stipa* L. y *Nassella* Desv. (Poaceae) en la Península Ibérica e Islas Baleares. *Acta Bot. Malac.* 21 125–189. 10.24310/abm.v21i0.8674

[B133] Vázquez Pardo F. M. Gutiérrez Esteban M. (2011). Classification of species of *Stipa* with awns having plumose distal segments. *Telopea* 13 155–176. 10.7751/telopea20116012

[B134] Veldkamp J. F. (1985). *Anemanthele* Veldk. (Gramineae: Stipeae), a new genus from New Zealand. *Acta Bot. Neerl.* 34 105–109. 10.1111/j.1438-8677.1985.tb01857.x

[B135] Vickery J. W. Jacobs S. W. L. Everett J. (1986). Taxonomic studies in *Stipa* (Poaceae) in Australia. *Telopea* 3 1–132. 10.7751/telopea19864701

[B136] Williams A. R. (2011). *Austrostipa* (Poaceae) subgenus *Lobatae* in Western Australia. *Telopea* 13 177–192. 10.7751/telopea20115013

[B137] Winterfeld G. (2006). Molekular-cytogenetische Untersuchungen an Hafergräsern (Aveneae) und anderen Poaceae. *Stapfia* 86 1–170.

[B138] Winterfeld G. Schneider J. Becher H. Dickie J. Röser M. (2015). Karyosystematics of the Australasian stipoid grass *Austrostipa* and related genera: chromosome sizes, ploidy, chromosome base numbers, and phylogeny. *Aust. Syst. Bot*. 28 145–159. 10.1071/SB14029

[B139] Wölk A. Röser M. (2014). Polyploid evolution, intercontinental biogeographical relationships and morphology of the recently described African oat genus *Trisetopsis* (Poaceae). *Taxon* 63 773–788. 10.12705/634.1

[B140] Wölk A. Röser M. (2017). Hybridization and long-distance colonization in oat-like grasses of South and East Asia, including an amended circumscription of *Helictotrichon* and the description of the new genus *Tzveleviochloa* (Poaceae). *Taxon* 66 20–43. 10.12705/661.2

[B141] Wu Z.-Y. Phillips S. M. (2006). “Stipeae,” in *Flora of China*, Vol. 2 eds Wu Z.-Y. Raven P. H. Hong D. Y. (Beijing: Science Press), 188–212.

[B142] Zhang Z.-S. Li L.-L. Chen W.-L. (2018). Chromosome number and karyotype of Phaenospermateae and Duthieeae (Poaceae), with reference to their systematic implications. *Nordic J. Bot.* 36:e01918 10.1111/njb.01918

