# Supplementary material for: Molecular Phylogenetics and Micromorphology of Australasian Stipeae (Poaceae, Subfamily Pooideae), and the Interrelation of Whole-Genome Duplication and Evolutionary Radiations in This Grass Tribe
Source: Front Plant Sci. 2021 Jan 22;11:630788. doi: 10.3389/fpls.2020.630788 (PMC7862344; doi:10.3389/fpls.2020.630788)

Supplementary Figure 2.
Maximum likelihood phylogram of all studied accessions of *Austrostipa* species inferred from ribosomal ITS1–5.8S gene–ITS2 DNA sequences with *Anisopogon avenaceus* (Duthieeae), *Bromus erectus* (Bromeae), *Hordeum vulgare* and *Secale sylvestre* (both Triticeae) used as outgroup. ML and MP bootstrap support values ≥ 50% as well as Bayesian PP ≥ 0.5 are indicated on the branches. Clades with ML support < 50% are collapsed. The taxonomic groupings of the *Austrostipa* species according to Jacobs and Everett (1996) and in this study are marked by different colors in columns 1 and 2. *A., Austrostipa*.

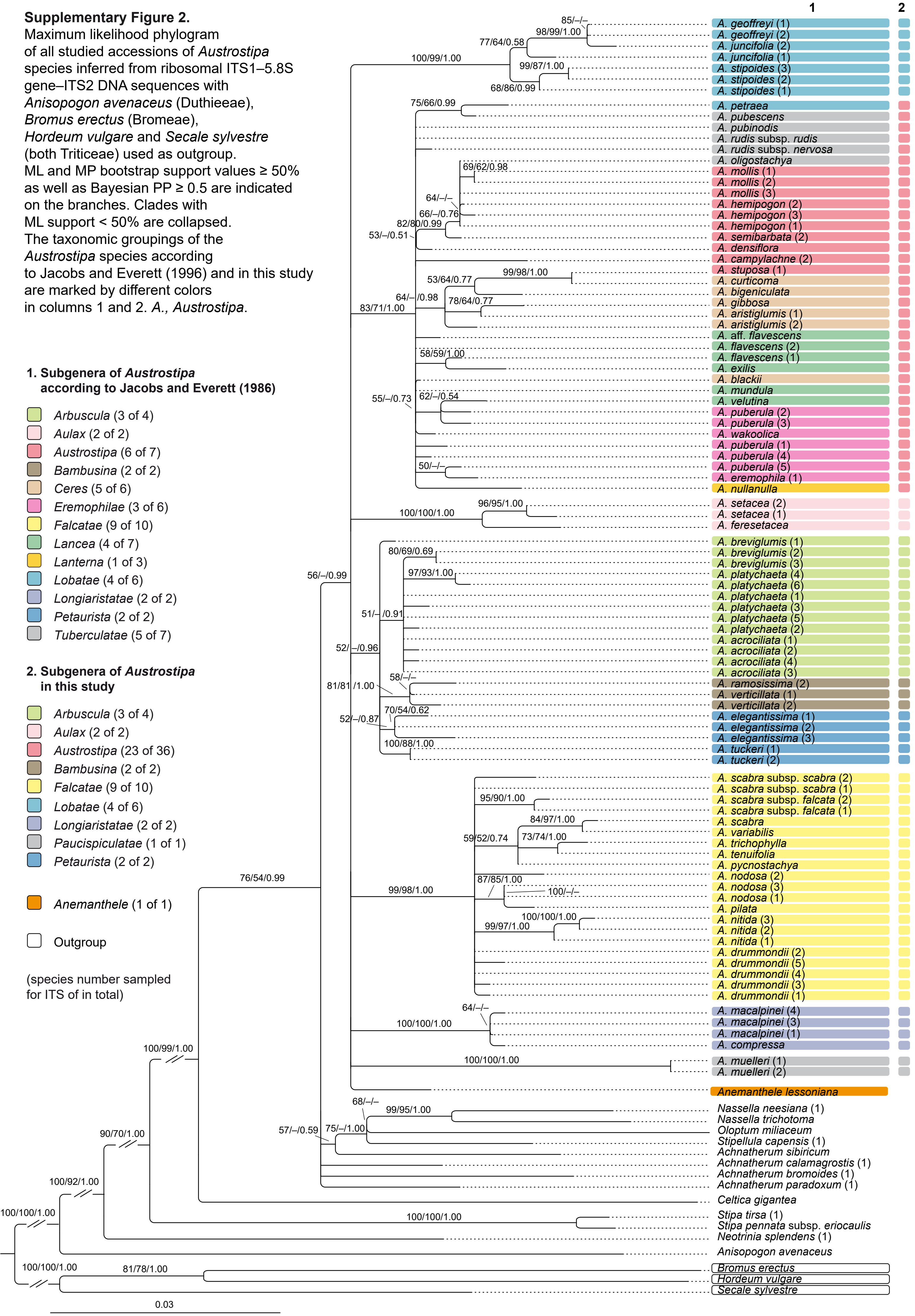

Supplement: Supplementary Figure 2 — Maximum likelihood phylogram of all studied accessions of Austrostipa species inferred from nuclear ribosomal ITS1–5.8S gene–ITS2 DNA sequences with Anisopogon avenaceus (Duthieeae), Bromus erectus (Bromeae), Hordeum vulgare, and Secale sylvestre (both Triticeae) used as outgroup. ML and MP bootstrap support values ≥ 50% as well as Bayesian PP ≥ 0.5 are indicated on the branches. Clades with ML support < 50% are collapsed. The taxonomic groupings of the Austrostipa species according to Jacobs and Everett (1996) and this study are marked by different colors in columns 1 and 2. A., Austrostipa. [file Image_2.pdf]
