# Supplementary material for: Molecular Phylogenetics and Micromorphology of Australasian Stipeae (Poaceae, Subfamily Pooideae), and the Interrelation of Whole-Genome Duplication and Evolutionary Radiations in This Grass Tribe
Source: Front Plant Sci. 2021 Jan 22;11:630788. doi: 10.3389/fpls.2020.630788 (PMC7862344; doi:10.3389/fpls.2020.630788)

Supplementary Figure 3.
Cluster analysis (UPGMA) performed on four micromorphological characters of 34 *Austrostipa* taxa. See **Supplementary Table 2** for the data matrix evaluated. The subgenera of *Austrostipa* are marked by different colors. *A., Austrostipa*.

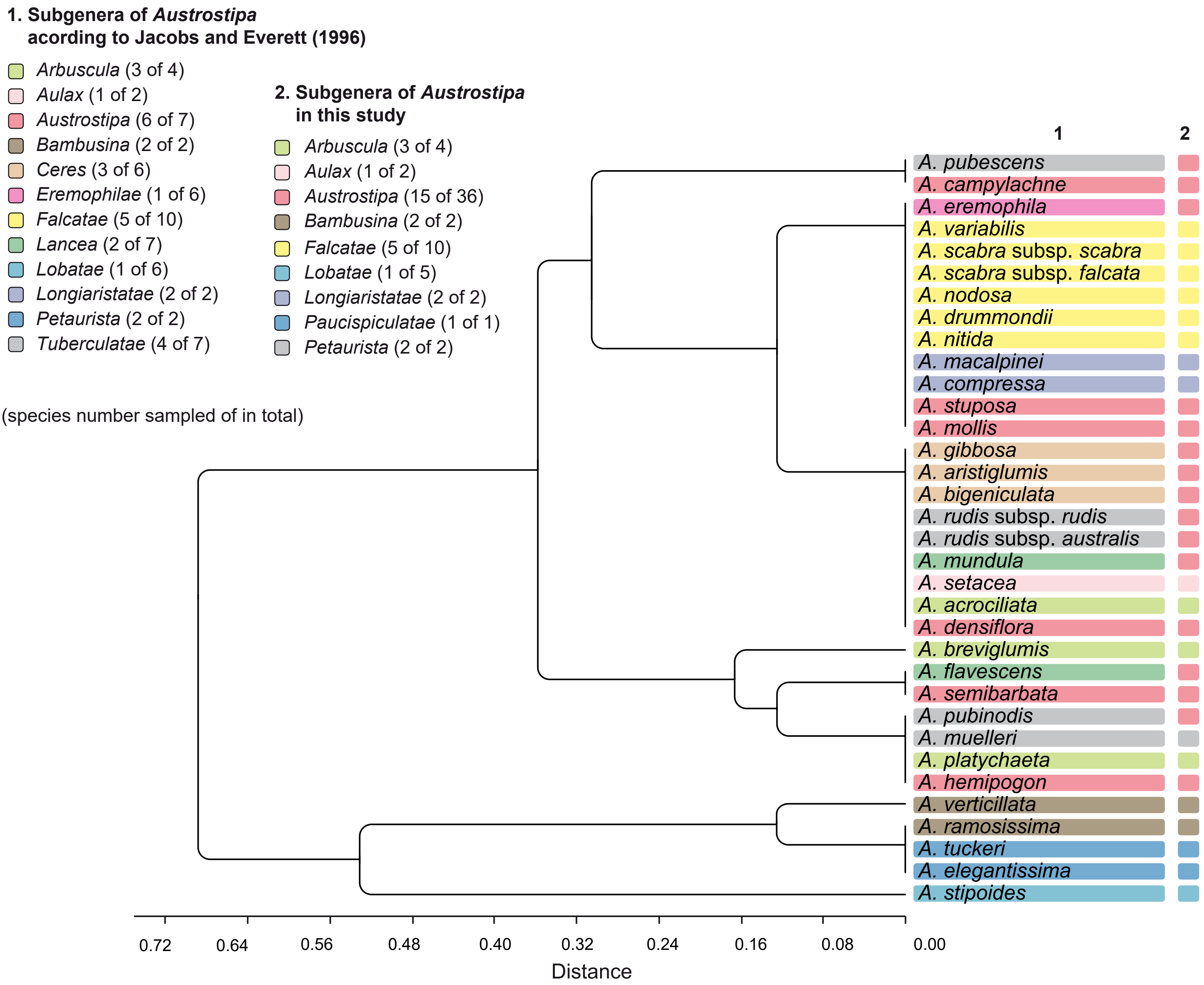

Supplement: Supplementary Figure 3 — Cluster analysis (UPGMA) performed on four micromorphological characters of 34 Austrostipa taxa. See Supplementary Table 2 for the data matrix evaluated. The taxonomic groupings of the Austrostipa species according to Jacobs and Everett (1996) and this study are marked by different colors in columns 1 and 2. A., Austrostipa. [file Image_3.pdf]
