# Supplementary material for: Molecular Phylogenetics and Micromorphology of Australasian Stipeae (Poaceae, Subfamily Pooideae), and the Interrelation of Whole-Genome Duplication and Evolutionary Radiations in This Grass Tribe
Source: Front Plant Sci. 2021 Jan 22;11:630788. doi: 10.3389/fpls.2020.630788 (PMC7862344; doi:10.3389/fpls.2020.630788)
Supplement: Supplementary Table 2 — Data matrix used for micromorphological and combined macro- and micromorphological analyses. See Table 3 for measurements and character coding. A., Austrostipa. [file Table_2.pdf]

Tkach, N., Nobis, M., Schneider, J., Becher, H., Winterfeld, G., Jacobs, S.W.L., Röser, M. 2021. Molecular phylogenetics and micromorphology of Australasian Stipeae (Poaceae, subfamily Pooideae), and the interrelation of whole-genome duplication and evolutionary radiations in this grass tribe. *Front. Plant Sci.* 11:630788. doi: 10.3389/fpls.2020.630788

Supplementary Table 2. Data matrix used for micromorphological and combined macro- and micromorphological analyses. See **Table 3** for measurements and character coding. *A.*, *Austrostipa*.

Taxon	Ligules [mm]	Glumes [mm]	Callus [mm]	Lemma [mm]	Lemma lobes [mm]	Awn [mm]	Length ratio lemma: palea	Inflores-cence branches	Funda-mental cells	Hooks	Silica cells	Cork cells
A. acrociliata	4.5	8	1.25	5.5	0.35	60	1	0	2	2	2	2
A. aristiglumis	1.1	11.5	0.7	5.75	0	32	1	0	2	2	2	2
A. bigeniculata	0.7	16	2	7.5	0	45	1	0	2	2	2	2
A. breviglumis	5	6	0.5	3.8	0	28	0.55	0	3	2	1	2
A. campylachne	0.45	18.5	2.2	8.5	0	60	1	0	1	1	2	2
A. compressa	7.5	17	3	7.5	0.2	110	1	0	1	2	2	2
A. densiflora	2	15	1.4	6.25	0	40	1	0	2	2	2	2
A. drummondii	1.6	9.5	2	5.5	0.15	75	0.9	0	1	2	2	2
A. elegantissima	2.5	10	0.65	7.25	0.8	35	0.5	1	3	1	2	1
A. eremophila	1	20	3	7.75	0.45	80	1	0	1	2	2	2
A. flavescens	0.45	13	2.3	7.25	0.25	55	0.9	0	1	2	1	2
A. gibbosa	0.4	14	1.25	5.25	0	37	1	0	2	2	2	2
A. hemipogon	2.5	17	2.3	6.4	0	45	1	0	2	2	1	2
A. macalpinei	8	17	2.5	7.5	0.25	130	1	0	1	2	2	2
A. mollis	2	17.5	2.2	8.3	0	80	1	0	1	2	2	2
A. muelleri	0.1	24	2.5	16.5	3	75	0.75	0	2	2	1	2
A. mundula	2	15	2.6	7.25	0.25	65	0.9	0	2	2	2	2
A. nitida	1	11	1.7	5	0.15	57	0.9	0	1	2	2	2
A. nodosa	1.3	12.5	1.9	5.5	0.2	72	1	0	1	2	2	2
A. platychaeta	4	12	0.75	5.25	0.15	75	0.7	0	2	2	1	2
A. pubescens	1	19.5	3.8	12.7	0	80	0.6	0	1	1	2	2
A. pubinodis	1.2	24	3.2	11.5	0	75	1	0	2	2	1	2
A. ramosissima	0.4	4	0.4	2.2	0	23	0.3	0	3	1	2	1
A. rudis subsp. *australis*	1.2	8.5	6.4	10	0	75	0.75	0	2	2	2	2
A. rudis subsp. *rudis*	1.2	13	2.8	8.5	0	75	0.75	0	2	2	2	2
A. scabra subsp. *falcata*	0.45	13	1.8	5.25	0.5	60	0.8	0	1	2	2	2
A. scabra subsp. *scabra*	1	13	1.5	5.25	0.5	50	0.8	0	1	2	2	2
A. semibarbata	1.25	23	3.2	10.2	0	90	1	0	1	2	1	2
A. setacea	7	13.5	2.15	6.25	0	32	1	0	2	2	2	2
A. stipoides	7.5	16	1.8	10.5	1.8	30	1	0	3	2	1	1
A. stuposa	0.75	19	2	9.5	0.15	55	1	0	1	2	2	2
A. tuckeri	4.5	7	0.25	4.5	0	32	0.3	1	3	1	2	1
A. variabilis	0.75	11.5	1.9	5.75	0.2	60	0.9	0	1	2	2	2
A. verticillata	5.5	3.5	0.4	3.25	0	47	0.45	0	2	1	2	1
