# Supplementary material for: Molecular Phylogenetics and Micromorphology of Australasian Stipeae (Poaceae, Subfamily Pooideae), and the Interrelation of Whole-Genome Duplication and Evolutionary Radiations in This Grass Tribe
Source: Front Plant Sci. 2021 Jan 22;11:630788. doi: 10.3389/fpls.2020.630788 (PMC7862344; doi:10.3389/fpls.2020.630788)
Supplement: Supplementary Appendix 1 — Taxa studied for DNA sequences, morphology and lemma micromorphology. Taxon, geographical origin, voucher information with collectors and herbarium code and ENA/GenBank accession numbers for plastid matK gene–3′trnK exon region; nuclear ribosomal ITS1–5.8S gene–ITS2 and nuclear single-copy gene Acc1. Sequences LR989057–LR989267 were newly generated for this study. A single asterisk (∗) indicates sequences previously generated in our lab, two asterisks (∗∗) indicate sequences taken from ENA/GenBank, a dash (–) missing data. The uppercase letters (A), (B) and (C) denote different Acc1 sequence copies. BG, Botanical Garden; LEP, lemma epidermal pattern studied; LEP ill., LEP illustrated in Figures 4, 5; morph, morphological data for Supplementary Table 2 taken from Everett et al. (2009); MSB, Millennium Seed Bank, Kew, Wakehurst Place; NSW, New South Wales; NT, Northern Territory; SA, South Australia; TAS, Tasmania; VIC, Victoria; WA, Western Australia. [file Data_Sheet_1.pdf]

Tkach, N., Nobis, M., Schneider, J., Becher, H., Winterfeld, G., Jacobs, S.W.L., Röser, M. 2021. Molecular phylogenetics and micromorphology of Australasian Stipeae (Poaceae, subfamily Pooideae), and the interrelation of whole-genome duplication and evolutionary radiations in this grass tribe. *Front. Plant Sci.* 11:630788. doi: 10.3389/fpls.2020.630788

Supplementary Appendix 1. Taxa studied for DNA sequences, morphology and lemma micromorphology. Taxon, geographical origin, voucher information with collectors and herbarium code and ENA/GenBank accession numbers for plastid *matK* gene–3′*trnK* exon region; nuclear ribosomal ITS1–5.8S gene–ITS2 and nuclear single-copy gene *Acc1*. Sequences LR989057–LR989267 were newly generated for this study. A single asterisk (*) indicates sequences previously generated in our lab, two asterisks (**) indicate sequences taken from ENA/GenBank, a dash (–) missing data. The uppercase letters (A), (B) and (C) denote different *Acc1* sequence copies. BG, Botanical Garden; LEP, lemma epidermal pattern studied; LEP ill., LEP illustrated in **Figures 4, 5**; morph, morphological data for **Supplementary Table 2** taken from Everett et al. (2009); MSB, Millennium Seed Bank, Kew, Wakehurst Place; NSW, New South Wales; NT, Northern Territory; SA, South Australia; TAS, Tasmania; VIC, Victoria; WA, Western Australia.

Achnatherum bromoides (L.) P.Beauv.: **(1)** FN434250*; FN434517*; –; **(2)** Iran, *T. Alexeenko* 316 (LE); LEP ill. ***A. calamagrostis*** (L.) P.Beauv.: **(1)** FN434255*; FN434520*; –; **(2)** Hungary, *H. Petry s.n.* (KRA); LEP ill. ***A. paradoxum*** (L.) Banfi, Galasso & Bartolucci: **(1)** grown in BG Halle, *M. Röser 11092*, 30 May 2012 (HAL); LR989197; LR989193; LR989116; **(2)** France, between Garabella and Montpellier, *J. Kornaś s.n.* (KRA); LEP ill. ***A. sibiricum*** (L.) Keng ex Tzvelev: Mongolia, Töv ajmag SE Ulaanbaatar; *H. Heklau s.n.*, 2006. Grown from seed in BG Halle no. R503 (no voucher); FN434339*; FN434595*; LR989117. ***Anemanthele lessoniana*** (Steud.) Veldkamp: Grown from seed obtained in 2007 from Royal Horticultural Society Garden, Wisley, U.K. in BG Halle no. R610 (no voucher); LN832437*, LR989198; LN832437*, LR989194; (A) LR989118, (B) LR989119. ***Anisopogon avenaceus*** R.Br.: AM234575*; FM179386*; –. ***Austrostipa acrociliata*** (Reader) S.W.L.Jacobs & J.Everett: **(1)** Australia, SA, 33°51'50.19"S, 136°8'55.88"E, *T.S. Te & P.J. Lang s.n.*, 26 Nov. 2007, collected seed at MSB no. 426442. Plants grown from seed at BG Halle by G. Winterfeld GW92 (HAL0137631); LN832440*; LN832412*; –; LEP; **(2)** Australia, VIC, 34°50'26"S, 142°17'53"E, *D. Roberts, N.G. Walsh & M.J. Hirst s.n.*, 23 Oct. 2007, collected seed at MSB no. 460352 (K). Plants grown from seed at BG Halle by G. Winterfeld GW97 (HAL0141317); LN832441*; LN832413*; (A) LR989057, (B) LR989058; LEP; **(3)** Australia, SA, Eyre Peninsula, c. 54 km SE of Streaky Bay, Port Kenny road, 33°7'11"S, 134°37'9"E, *S.W.L. Jacobs 9760 & M.E. Barkworth*, 06 Dec. 2007 (AD229036, NSW832532); LR989199; LR989126; –; **(4)** Australia, WA, Eyre, Hopetoun, 33°56'44"S, 120°07'34"E, *S.W.L. Jacobs 9746 & M.E. Barkworth*, 02 Dec. 2007 (HAL0110716, duplicates: NSW832487, PERTH8048193, UTC00252175); LR989200; LR989127; –; LEP. ***A. aphylla*** (Rodway) S.W.L.Jacobs & J.Everett: morph. ***A. aquarii*** (Vickery, S.W.L.Jacobs & J.Everett) S.W.L.Jacobs & J.Everett: morph. ***A. aristiglumis*** (F.Muell.) S.W.L.Jacobs & J.Everett **(1)** Australia, VIC, Murray Mallee, c. 11 km S of Hopetoun, Warracknabeal road, 35°48'45"S, 142°25'22"E, *S.W.L. Jacobs 9777 & M.E. Barkworth*, 09 Dec. 2007 (HAL0110797, duplicates: B, BA, K, MEL2330408A, NSW594293, TCD, US, UTC); LR989202; LR989128; –; LEP ill.; **(2)** Australia, NSW, South Western Plains, c. 1 km S of Darlington Point, 34°35'04"S, 145°59'40"E, *S.W.L. Jacobs 9795 & M.E. Barkworth*, 12 Dec. 2007 (HAL0107516, duplicates: BA, NSW594352, UTC); LR989203; LR989129; –; LEP. ***A. bigeniculata*** (Hughes) S.W.L.Jacobs & J.Everett: Australia, NSW, Southern Tablelands, c. 17 km E of Bungonia, Goulburn road, 34°48'45"S, 149°47'03"E, *S.W.L. Jacobs 9728 & M.E. Barkworth*, 23 Nov. 2007 (HAL0110805, duplicates: B, BA, K, NSW832478, TCD, UTC00252175); LR989204; LR989130; –; LEP ill. ***A. blackii*** (C.E.Hubb.) S.W.L.Jacobs & J.Everett: Australia, SA, *D.J. & M.K. Jones s.n.*, 22 Nov. 2005, collected seed at

MSB no. 297934 (K). Plants grown from seed at BG Halle by G. Winterfeld GW53 (HAL0140625, HAL0137622); LN832453*; LN832421*; (A) LR989059, (B) LR989060. ***A. breviglumis*** (J.M.Black) S.W.L.Jacobs & J.Everett: **(1)** Australia, SA, 34°52'30.4"S, 138°42'49.46"E, 20 Dec. 2005, collected seed at MSB no. 302966 (K). Plants grown from seed at BG Halle by G. Winterfeld GW56 (HAL0141315, HAL0137637); LN832442*; LN832414*; LR989061; **(2)** Australia, SA, Eyre Peninsula, c. 135 km SE of Elliston, Port Lincoln rd, 34°35'04"S, 135°35'06"E, *S.W.L. Jacobs 9766 & M.E. Barkworth*, 06 Dec. 2007 (HAL0110713, duplicates: AD228918, UTC00252101, NSW832490); LR989205; LR989131; –; LEP ill.; **(3)** same collection as (2); LR989206; LR989132; -. ***A. campylachne*** (Nees) S.W.L.Jacobs & J.Everett**: (1)** Australia, WA, 12.5 km E of Catchment Road on Qualen Road, 32°5'16.7"S, 116°40'14.2"E, *A. Crawford ADC 987*, 26 Nov. 2005, collected seed at MSB no. 376226 (K, PERTH7346883). Plants grown from seed at BG Halle by G. Winterfeld GW89 (HAL0137630); LN832445*; –; (A) LR989062, (B) LR989063; **(2)** –; GU254627; –; **(3)** Australia, WA, Smith's Mill, *A. Morrison s.n.*, 13 Nov. 1897 (E690354); LEP ill. ***A. centralis*** (Vickery, S.W.L.Jacobs & J.Everett) S.W.L.Jacobs & J.Everett: morph. ***A. compressa*** (R.Br.) S.W.L.Jacobs & J.Everett: **(1)** –; EU489100**; –; **(2)** Australia, WA, Cottesloe, *A. Morrison s.n.*, 21.10.1899 (E690358); LEP ill. ***A. crinita*** (Gaudich.) S.W.L.Jacobs & J.Everett: morph. ***A. curticoma*** (Vickery) S.W.L.Jacobs & J.Everett: LN832454*; LN832422*; –. ***A. densiflora*** (Hughes) S.W.L.Jacobs & J.Everett: Australia, NSW, Southern Tablelands, Bungonia turnoff, S of Marulan, Hume Highway, 34°43'59"S, 149°58'47"E, *S.W.L. Jacobs 9725*, 23 Nov. 2007 (HAL0110782, duplicates: NSW832480, UTC00252106); LR989207; LR989133; –; LEP ill. ***A. dongicola*** (Vickery, S.W.L.Jacobs & J.Everett) S.W.L.Jacobs & J.Everett: morph. ***A. drummondii*** (Steud.) S.W.L.Jacobs & J.Everett: **(1)** LN832455*; LN832423*; –; **(2)** Australia, WA, Dundas (S) Eucla, base of Eucla Pass, 31°40'52"S, 128°52'30"E., *S.W.L. Jacobs 9752 & M.E. Barkworth*, 04 Dec. 2007 (NSW832530, PERTH8500231); LR989208; LR989134; –; **(3)** Australia, SA, South-eastern, c. 28 km E of Woods Well, Tintinara road, 35°58'25"S, 139°50'25"E, *S.W.L. Jacobs 9776 & M.E. Barkworth*, 09 Dec. 2007 (HAL0110798, duplicates: B, BA, MEL2330245A, NSW594288, TCD, UTC); LR989209; LR989135; –; LEP ill.; **(4)** Australia, SA, Nullarbor, c. 76 km E of Border Village, Nullarbor road, 31°36'04"S, 129°46'30"E, *S.W.L. Jacobs 9757 & M.E. Barkworth*, 05 Dec. 2007 (HAL064847, duplicates: AD229038, BA, NSW832524, TCD, UTC); LR989210; LR989136; –; LEP; **(5)** Australia, WA, Eucla, base of Eucla Pass, 31°40'52"S, 128°52'30"E, *S.W.L. Jacobs 9754 & M.E. Barkworth*, 04 Dec. 2007 (HAL0110717, duplicates: NSW594291, PERTH8003912, UTC00252318); LR989211; LR989137; –; LEP. ***A. echinata*** (Vickery, S.W.L.Jacobs & J.Everett) S.W.L.Jacobs & J.Everett: morph. ***A. elegantissima*** (Labill.) S.W.L.Jacobs & Everett: **(1)** Australia, SA, 34°52'26.15"S, 138°42'44.73"E, 20 Dec. 2005, collected seed at MSB no. 302977 (K). Plants grown from seed at BG Halle by G. Winterfeld GW57 (HAL0137633); LN832467*; LR989138; (A) LR989064, (B) LR989065, (C) LR989066; **(2)** Australia, WA, Roe, c. 68 km S of Norseman, Salmon Gums road, 32°44'31"S, 121°32'08", *S.W.L. Jacobs 9736 & M.E. Barkworth*, 01 Dec. 2007 (HAL0111057, duplicates: NSW832555, PERTH8256861, UTC); LR989212; LR989139; –; LEP; **(3)** Australia, WA, Eyre, c. 50 km E of Ravensthorpe, Esperance Road, 33°40'17"S, 120°39'34"E, *S.W.L. Jacobs 9749 & M.E. Barkworth*, 03 Dec. 2007 (HAL0110796, duplicates: NSW594284, PERTH8003912, TCD, UTC00252324); LR989213; LR989140; –; LEP ill.; **(4)** Australia, SA, Murray Mallee, between Kingston and Waikerie, *D.J.E. Whibley 2651*, 28 Sep. 1968 (E690373); LEP. ***A. eremophila*** (Reader) S.W.L.Jacobs & J.Everett: **(1)** Australia, WA, Eyre, c. 4 km N of Esperance, Ravensthorpe road, 33°50'8"S, 121°51'40"E, *S.W.L. Jacobs 9739 & M.E. Barkworth*, 01 Dec. 2007 (NSW832514, PERTH8003882, UTC00252213); LR989214; LR989141; –; **(2)** Australia, SA, Redcliff Survey Area, 33°43'S, 137°51'E, *R.J. Chinnock 1594* (E690379); LEP ill. ***A. exilis*** (Vickery) S.W.L.Jacobs & J.Everett: Australia, WA, Esperance (S), Mondrain Island, off Esperance, NE slope of N part of Island, 34°7'18"S, 122°14'38"E, *J.A. Cochrane 4286*, 20 Nov. 2002, collected seed at MSB no. 359476 (K,

PERTH6476562). Plants grown from seed at BG Halle by G. Winterfeld GW84 (HAL0140634, HAL0137636); LN832462*; LN832430*; (A) LR989067, (B) LR989068, (C) LR989069. ***A. feresetacea*** (Vickery, S.W.L.Jacobs & J.Everett) S.W.L.Jacobs & J.Everett: –; JF769061**; –. ***A. flavescens*** (Labill.) S.W.L.Jacobs & J.Everett: **(1)** Australia, TAS, 41°24'29.8"S, 148°16'54.5"E, 13 Dec. 2007, collected seed at MSB no. 442387 (K). Plants grown from seed at BG Halle by G. Winterfeld GW98 (no voucher); LN832463*; LN832431*; (A) LR989070, (B) LR989071; **(2)** Australia, SA, South-eastern, c. 18 km S of Rode, Beachport Road, 37°14'38"S, 139°54'22"E, *S.W.L. Jacobs 9684*, 24 Oct. 2007 (HAL0110799, duplicates: AD228901, NSW831786, UTC00252189); LR989215; LR989142; –; LEP ill. ***A.* aff. *flavescens*** (Labill.) S.W.L.Jacobs & J.Everett: Australia, SA, c. 7 km NW Port Lincoln, Elliston Road, along powerline track near road, 34°44'1"S, 135°8'49"E, *S.W.L. Jacobs 9767, 06 Dec. 2007* (NSW860187); LR989201; LR989143; –. ***A. geoffreyi*** S.W.L.Jacobs & J.Everett: **(1)** Australia, WA, Lake Grace (S), 33°5'23"S, 119°33'45"E, *J.A. Cochrane 4243*, 06 Nov. 2002, collected seed at MSB no. 213064 (K, PERTH6476740). Plants grown from seed at BG Halle by G. Winterfeld GW44 (no voucher); LN832465*; LN832433*; (A) LR989072, (B) LR989073; **(2)** Australia, WA, Roe, Lake King, c. 11.5 km W of Lake King township, 33°05'23"S, 119°33'20"E, *S.W.L. Jacobs 9743 & M.E. Barkworth*, 02 Dec. 2007 (HAL0110783, UTC00252284, NSW832518); LR989216; LR989144; –. ***A. gibbosa*** (Vickery) S.W.L.Jacobs & J.Everett: Australia, SA, Northern Lofty, c. 19 km SE of Crystal Brook, Clare road, 33°26'16"S, 138°22'11"E, *S.W.L. Jacobs 9773 & M.E. Barkworth*, 08 Dec. 2007 (HAL0110795, duplicates: AD228915, BA, NSW594286, TCD, UTC00252323); LR989217; LR989145; –; LEP ill. ***A. hemipogon*** (Benth.) S.W.L.Jacobs & J.Everett: **(1)** LN832446*; LN832417*; –; **(2)** Australia, WA, Albany (C), Barrs Road Reserve, 34°18'24"S, 118°26'49"E, *J.A. Cochrane 5022*, 01 Nov. 2004, collected seed at MSB no. 359502 (K). Plants grown from seed at BG Halle by G. Winterfeld GW85 (HAL0140623, HAL0137629); LN832447*; LN832418*; (A) LR989074, (B) LR989075, (C) LR989076; **(3)** Australia, WA, Roe, Lake King, island c. 8 km W of Lake King township, 33°05'26"S, 119°35'32"E, *S.W.L. Jacobs 9744 & M.E. Barkworth*, 02 Dec. 2007 (HAL0110789, duplicates: NSW832519, PERTH8003904, UTC00252265); LR989218; LR989146; –; LEP ill.; **(4)** Australia, WA, Roe, c. 13 km S of Grass patch, Esperance Road, 33°20'48"S, 121°42'48"E, *S.W.L. Jacobs 9737a & M.E. Barkworth*, 01 Dec. 2007 (HAL0110788, duplicates: NSW832517, PERTH5038308, UTC00252282, UTC); LEP. ***A. juncifolia*** (Hughes) S.W.L.Jacobs & J.Everett: **(1)** Australia, SA, Eyre, 58 km S of Grass Patch, Esperance road, 33°42'31"S, 121°50'21"E, *S.W.L. Jacobs 9738 & M.E. Barkworth*, 01 Dec. 2007 (HAL0111022, duplicates: NSW832548, PERTH5038308, UTC00259369); LR989219; LR989147; –; **(2)** Australia, WA, Roe, Lake King, Western shore. W of Lake King township, 33°05'24"S, 119°32'42"E, *S.W.L. Jacobs 9742 & M.E. Barkworth*, 02 Dec. 2007 (HAL0107543, duplicates: NSW832527, UTC); LR989220; LR989148; –. ***A. lanata*** (Vickery, S.W.L.Jacobs & J.Everett) S.W.L.Jacobs & J.Everett: morph. ***A. macalpinei*** (Reader) S.W.L.Jacobs & Everett: **(1)** Australia, WA, 31°2'22"S, 116°2'37"E, *L. Lumbus s.n.*, 12 Nov. 2003, collected seed at MSB no. 230847 (K). Plants grown from seed at BG Halle by G. Winterfeld GW47 (no voucher); LR989221; LR989149; (A) LR989077, (B) LR989078; **(2)** Australia, WA, Eyre, c. 70 km E of Ravensthorpe, Esperance Road, 33°40'47"S, 120°45'38"E, *S.W.L. Jacobs 9740 & M.E. Barkworth*, 01 Dec. 2007 (HAL0110785, duplicates: NSW832512, PERTH8003955, UTC00252316, UTC); LR989222; –; –; LEP ill.; **(3)** Australia, WA, Eyre, c. 40 km E of Ravensthorpe, Esperance road, 33°38'53"S, 120°26'58"E, *S.W.L. Jacobs 9748 & M.E. Barkworth*, 03 Dec. 2007 (NSW832528, PERTH8500223); LR989223; LR989150; –; **(4)** same collection as (2); LR989224; LR989151; –; LEP. ***A. metatoris*** (J.Everett & S.W.L.Jacobs) S.W.L.Jacobs & J.Everett: morph. ***A. mollis*** (R.Br.) S.W.L.Jacobs & J.Everett: **(1)** Australia, SA, 35°10'0.09"S, 138°50'44.27"E, *D.J. Duval s.n.*, 26 Nov. 2006, collected seed at MSB no. 359904 (K). Plants grown from seed at BG Halle by G. Winterfeld GW79 (HAL0141318, HAL0137620); LN832448*; LN832419*; (A) LR989079, (B) LR989080, (C)

LR989081; **(2)** Australia, VIC, Grampians, Rose Creek road, c. 55 km SE of Horsham. Grampians National Park, 37°07'33"S, 142°24'46"E, *S.W.L. Jacobs 9787 & M.E. Barkworth*, 11 Dec. 2007 (HAL0107541, duplicates: MEL2340051A, NSW594349, UTC); LR989225; LR989152; –; LEP ill.; **(3)** Australia, SA, South-eastern, c. 18 km S of Pinaroo, Bordertown Road, 35°23'44"S, 140°49'14"E, *S.W.L. Jacobs 9701*, 29 Oct. 2007 (HAL0110800, duplicates: AD228900, BA, NSW831784, UTC00252178); LR989226; LR989153; –; LEP. ***A. muelleri*** (Tate) S.W.L.Jacobs & Everett: **(1)** Australia, VIC, Grampians, Harrops Track, c. 1 km N of Grahams Creek (c. 9 km N Glenelg River Road.), 37°18'14"S, 142°15'04"E, *S.W.L. Jacobs 9790 & M.E. Barkworth*, 11 Dec. 2007 (HAL0110712, duplicates: BA, MEL2330272A, NSW832495, UTC00252111); LR989227; LR989154; –; LEP ill.; **(2)** Australia, VIC, Grampians, Grampians National Park, junction of Glenelg River Road and Harrop Track, 37°22'7"S, 142°11'56"E, *S.W.L. Jacobs 9791 & M.E. Barkworth*, 11 Dec. 2007 (MEL2340048A, NSW594311); LR989228; LR989155; –. ***A. multispiculis*** (J.M.Black) S.W.L.Jacobs & J.Everett: morph. ***A. mundula*** (J.M.Black) S.W.L.Jacobs & J.Everett: Australia, SA, Eyre Peninsula, Tooligie, c. 45 km N of Cummins, 33°51'42"S, 135°42'20"E, *S.W.L. Jacobs 9768 & M.E. Barkworth*, 06 Dec. 2007 (HAL0110714, duplicates: AD228922, NSW832492, UTC00252183); LR989229; LR989156; –; LEP. ***A. nitida*** (Summerh. & C.E.Hubb.) S.W.L.Jacobs & J.Everett: **(1)** Australia, NSW, South Western Plains, c. 40 km N of Goolgowi, Hillston road, 33°39'43"S, 145°32'33"E, *S.W.L. Jacobs 9803 & M.E. Barkworth*, 12 Dec. 2007 (HAL0107550, duplicates: B, BA, G-DC, NSW594317, TCD, US, UTC); LR989230; LR989157; –; LEP; **(2)** Australia, SA, Eyre Peninsula, c. 40 km E of Kimba, Port Augusta road, 32°55'53"S, 136°55'34"E, *S.W.L. Jacobs 9769 & M.E. Barkworth*, 07 Dec. 2007 (HAL0111023, duplicates: AD238077, B, G, K, NSW832547, TCD, UTC); LR989231; LR989158; –; LEP ill.; **(3)** Australia, WA, Roe, c. 40 km S of Norseman, Salmon Gums road, 32°19'09"S, 121°45'33"E, *S.W.L. Jacobs 9731 & M.E. Barkworth*, 01 Dec. 2007 (HAL0111024, duplicates: NSW832550, PERTH8256837, UTC); LR989232; LR989159; –; LEP. ***A. nivicola*** (J.H.Willis) S.W.L.Jacobs & J.Everett: morph. ***A. nodosa*** (S.T.Blake) S.W.L.Jacobs & J.Everett: **(1)** Australia, VIC, Wimmera, c. 23 km SW of Warracknabeal, Dimboola Road, 36°22'05"S, 142°10'55"E, *S.W.L. Jacobs 9780 & M.E. Barkworth*, 10 Dec. 2007 (HAL0110711, duplicates: B, BA, G, K, MEL2331037A, NSW832494, UTC00252262); LR989233; LR989160; –; LEP ill.; **(2)** Australia, SA, Eyre Peninsula, c. 3 km SE of Elliston, Port Lincoln road, 33°39'37"S, 134°54'53"E, *S.W.L. Jacobs 9762 & M.E. Barkworth*, 06 Dec. 2007 (HAL0110784, duplicates: AD228913, BA, NSW832520, UTC00252263); LR989234; LR989161; –; LEP; **(3)** LN832456*; LN832424*; –. ***A. nullanulla*** (J.Everett & S.W.L.Jacobs) S.W.L.Jacobs & J.Everett: Australia, SA, 31°39'47.25"S, 135°15'22.34"E, *D.J. Duval, B. Haas & P. Swanson s.n.*, 09 Nov. 2005, collected seed at MSB no. 291460 (K). Plants grown from seed at BG Halle by G. Winterfeld GW66 (no voucher); LN832464*; LN832432*; (A) LR989082, (B) LR989083. ***A. nullarborensis*** (Vickery, S.W.L.Jacobs & J.Everett) S.W.L.Jacobs & J.Everett: morph. ***A. oligostachya*** (Hughes) S.W.L.Jacobs & J.Everett: Australia, SA, 35°35'24.29"S, 138°33'29.35"E, *T.S. Te, D.J. Duval & M.J. Thorpe s.n.*, 07 Nov. 2006, collected seed at MSB no. 359889 (K). Plants grown from seed at BG Halle by G. Winterfeld GW78 (HAL0141319, HAL0141389, HAL0137624); LN832468*; LN832436*; (A) LR989084, (B) LR989085, (C) LR989086. ***A. petraea*** (Vickery) S.W.L.Jacobs & J.Everett: Australia, SA, Flinders Ranges National Park, Bunyeroo Gorge, South Flinders Ranges, 31°25'9"S, 138°33'48"E., *S.W.L. Jacobs 9698*, 28 Oct. 2007 (NSW831779); LR989235; LR989162; (A) LR989087, (B) LR989088. ***A. pilata*** (S.W.L.Jacobs & J.Everett) S.W.L.Jacobs & J.Everett: LN832461*; LN832429*; –. ***A. platychaeta*** (Hughes) S.W.L.Jacobs & J.Everett: **(1)** Australia, SA, Murray, c. 3 km N of Cambrai, Sedan Road, 34°37'39"S, 139°16'55"E, *S.W.L. Jacobs 9700*, 29 Oct. 2007 (HAL0110801, duplicates: AD229030, NSW831785, UTC00252179); LR989236; LR989163; –; LEP; **(2)** Australia, WA, Coolgardie, c. 47 km S of Norseman, Salmon Gums road, 32°34'2"S, 121°35'27"E, *S.W.L. Jacobs 9735 & M.E. Barkworth*, 01 Dec. 2007 (NSW832549, PERTH8256845);

LR989237; LR989164; –; **(3)** Australia, VIC, Wimmera, c. 21 km SW of Warracknabeal, Dimboola road, 36°21’23”S, 142°11’56”E, *S.W.L. Jacobs 9778 & M.E. Barkworth*, 10 Dec. 2007 (HAL0110787, duplicates: B, MEL2330210A, NSW832516, TCD, UTC00252268); LR989238; LR989165; –; LEP; **(4)** Australia, SA, Nullarbor, c. 10 km N of Nullarbor Roadhouse, Murrawinje Cave Road, 31°21’60”S, 130°52’17”E, *S.W.L. Jacobs 9759 & M.E. Barkworth*, 05 Dec. 2007 (NSW832531); LR989239; LR989166; –; **(5)** Australia, WA, Eucla, c. 2 km E of Eucla, Border Village, 31°40’15”S, 128°54’24”E, *S.W.L. Jacobs 9755 & M.E. Barkworth*, 04 Dec. 2007 (HAL0110718, duplicates: BA, NSW594292, PERTH8003920, UTC00252319); LR989240; LR989167; –; LEP ill.; **(6)** Australia, WA, Roe, c. 4 km S of Norseman, Salmon Gums road, 32°19’09”S, 121°45’33”E, *S.W.L. Jacobs 9730 & M.E. Barkworth*, 01 Dec. 2007 (HAL0110801, duplicates: AD, NSW832551, PERTH8256918, UTC); LR989241; LR989168; –; LEP. ***A. plumigera*** (Hughes) S.W.L.Jacobs & J.Everett; morph. ***A. puberula*** (Steud.) S.W.L.Jacobs & J.Everett: **(1)** Australia, WA, Coolgardie, c. 47 km S of Norseman, Salmon Gums rd, 32°34’02”S, 121°35’28”E, *S.W.L. Jacobs 9734 & M.E. Barkworth*, 01 Dec. 2007 (HAL0110709, duplicates: BA, NSW832491, PERTH8048177, UTC00252184); LR989242; LR989169; –; **(2)** Australia, VIC, Wimmera, c. 21 km SW of Warracknabeal, Dimboola road, 36°21’23”S, 142°11’56”E, *S.W.L. Jacobs 9779*, 10 Dec. 2007 (HAL0110786, duplicates: BA, MEL2330238A, NSW832515, TCD, UTC00252266); LR989243; LR989170; –; **(3)** Australia, SA, Nullarbor, c. 42 km E of Border Village, Nullarbor road, 31°37’57”S, 129°25’47”E, *S.W.L. Jacobs 9756 & M.E. Barkworth*, 05 Dec. 2007 (HAL0107544, duplicates: AD229037, BA, NSW832525, UTC); LR989244; LR989171; (A) LR989089, (B) LR989090; **(4)** Australia, SA, South-eastern, c. 6 km E of Woods Well, Tintinara Road, 35°59’11”S, 139°36’22”E, *S.W.L. Jacobs 9775 & M.E. Barkworth*, 09 Dec. 2007 (HAL0110710, duplicates: AD228898, B, BA, CAN, K, NSW832493, PE, TCD, US, UTC00252185); LR989245; LR989172; –; **(5)** Australia, SA, Eyre Peninsula, c. 15 km SE of Port Kenny, Elliston road, 33°16’19”S, 134°47’15”E, *S.W.L. Jacobs 9761 & M.E. Barkworth*, 06 Dec. 2007 (HAL0111026, duplicates: AD238076, B, BA, G, GRA, K, NSW832554, TCD, UTC); LR989246; LR989173; –. ***A. pubescens*** (R.Br.) S.W.L.Jacobs & J.Everett: Australia, NSW, Central Coast, Kentlyn, 34°03’21”S, 150°52’32”E, S.*W.L. Jacobs 9724 & M.E. Barkworth*, 20 Nov. 2007 (HAL0110778, duplicates: NSW832477, UTC00252176); LR989247; LR989174; –; LEP ill. ***A. pubinodis*** (Trin & Rupr.) S.W.L.Jacobs & J.Everett: Australia, NSW, Grampians, Rose Creek road, c. 55 km SE of Horsham, Grampians National Park, 37°07’33”S, 142°24’46”E, *S.W.L. Jacobs 9788 & M.E. Barkworth*, 11 Dec. 2007 (HAL0107511, duplicates: MEL2340073A, NSW594312, UTC); LR989248; LR989175; –; LEP ill. ***A. pycnostachya*** (Benth.) S.W.L.Jacobs & J.Everett; –; EU489101**; –. ***A. ramosissima*** (Trin.) S.W.L.Jacobs & J.Everett: **(1)** Australia, QLD, 27°28’25.8”S, 149°16’52.4”E, *T. Tyson-Doneley s.n.*, 10 May 2005, collected seed at MSB no. 273060 (K). Plants grown from seed at BG Halle by G. Winterfeld GW65 (HAL0137634); LN832451*; –; (A) LR989091, (B) LR989092; **(2)** Australia, NSW, Central Coast, Mount Annan BG, 34°04’17”S, 150°46’03”E, *S.W.L. Jacobs 9831 & M.E. Barkworth*, 08 Jan. 2008 (HAL0107512, duplicates: BA, NSW594356, UTC); LR989249; LR989176; –; LEP ill. ***A. rudis*** (Spreng.) S.W.L.Jacobs & Everett subsp. ***rudis***: Australia, VIC, Gippsland Plain, c. 3 km N of Sale, Bairnsdale Road, 38°04’45”S, 147°04’18”E, *S.W.L. Jacobs 9710*, 31 Oct. 2007 (HAL0110802, duplicates: MEL2330239A, NSW831783, UTC00252181); LR989250; LR989178; –; LEP. ***A. rudis*** subsp. ***australis*** (J.Everett & S.W.L.Jacobs) S.W.L.Jacobs & J.Everett: Australia, VIC, Ringwood, *A. Morrison s.n.*, 21.10.1891 (E692076); LEP ill. ***A. rudis*** subsp. ***nervosa*** (Vickery) S.W.L.Jacobs & J.Everett: Australia, NSW,Central Coast, Georges River, Appin, 34°12’35”S, 150°47’46”E, *S.W.L. Jacobs 9722 & M.E. Barkworth*, 20 Nov. 2007 (HAL0110779, duplicates: BA, NSW832476, UTC00252104); LR989251; LR989177; –. ***A. scabra*** (Lindl.) S.W.L.Jacobs & J.Everett: Australia, SA, 35°10’0.09”S, 138°50’44.27”E, *D.J. Duval s.n.*, 01 Dec. 2006, collected seed at MSB no. 359926 (K). Plants grown from seed at BG Halle by G. Winterfeld GW80 (no voucher); LN832458*; LN832426*; (A)

LR989093, (B) LR989094, (C) LR989095. ***A. scabra*** subsp. ***scabra*: (1)** Australia, TAS, 42°41’33.7”S, 147°16’0”E, *J.A. Wood s.n.*, 03 Dec. 2007, collected seed at MSB no. 463744 (K). Plants grown from seed at BG Halle by G. Winterfeld GW112 (HAL0140650, HAL0137627); LN832459*; LN832427*; (A) LR989096, (B) LR989097; **(2)** Australia, NSW, South Western Plains, c. 82 km N of Hillston, Mount Hope road, 32°55’52”S, 145°52’38”E, *S.W.L. Jacobs 9804 & M.E. Barkworth*, 13 Dec. 2007 (HAL0110793, duplicates: BA, NSW594289, UTC00252321); LR989252; LR989179; –; LEP ill. ***A. scabra*** subsp. ***falcata*** (Hughes) S.W.L.Jacobs & J.Everett: **(1)** Australia, QLD, 26°29’1.68”S, 151’20’2.4”E, *D. Bowen & P. Boyle s.n.*, 25 Nov. 2005, collected seed at MSB no. 366454 (K). Plants grown from seed at BG Halle by G. Winterfeld GW63 (HAL0137626); LN832457*; LR989180; (A) LR989098, (B) LR989099, (C) LR989100; **(2)** Australia, NSW, Southern Tablelands, Bungonia turnoff, S of Marulan, Hume Highway, 34°43’59”S, 149°58’47”E, *S.W.L. Jacobs 9726 & M.E. Barkworth*, 23 Nov. 2007 (HAL0110781, duplicates: BA, NSW832479, UTC00252107, UTC); LR989253; LR989181; (A) LR989101, (B) LR989102, (C) LR989103; LEP ill. ***A. semibarbata*** (R.Br.) S.W.L.Jacobs & J.Everett: **(1)** Australia, SA, 34°57’51.26”S, 138°42’39.79”E, *R.J. Bates s.n.*, 15 Nov. 2006, collected seed at MSB no. 368768 (K). Plants grown from seed at BG Halle by G. Winterfeld GW64 (HAL0137628); LR989254; –; (A) LR989104, (B) LR989105, (C) LR989106; **(2)** Australia, VIC, Gippsland Plain, 33 km E of Longford, Loch Sport Road, 38°08’26”S, 147°24’23”E, *S.W.L. Jacobs 9709*, 31 Oct. 2007 (HAL0110777, duplicates: MEL2330348A, NSW831776, UTC00252208); LR989255; LR989182; –; LEP ill. ***A. setacea*** (R.Br.) S.W.L.Jacobs & J.Everett: **(1)** Australia, NSW, 34°4’4”S, 150°45’57”E, 18 Nov. 2003, collected seed at MSB no. 225014 (K). Plants grown from seed at BG Halle by G. Winterfeld GW55 (no voucher); LN832444*; LN832416*; (A) LR989107, (B) LR989108; **(2)** Australia, VIC, Grampians, c. 11 km W of Natimuk, Mount Arapiles-Tooan State Park, 36°45’39”S, 141°50’13”E, *S.W.L. Jacobs 9781 & M.E. Barkworth*, 10 Dec. 2007 (HAL0110806, duplicates: MEL2330274A, NSW832509, UTC); LR989256; LR989183; –; LEP ill.; **(3)** Australia, VIC, Grampians, c. 11 km W of Natimuk; Mount Arapiles-Tooan State Park, 36°45’39”S, 141°50’13”E, *S.W.L. Jacobs 9785 & M.E. Barkworth*, 10 Dec. 2007 (HAL0110715, duplicates: MEL2330242A, NSW832488, UTC); LEP. ***A. stipoides*** (Hook.f.) S.W.L. Jacobs & Everett: **(1)** Australia, NSW, South Coast, Bermagui, 36°25’39”S, 150°4’29”E, *S.W.L. Jacobs 9715 & M.E. Barkworth*, 01 Nov. 2007 (NSW832485, UTC00252109); LR989257; LR989184; –; **(2)** Australia, SA, South-eastern, c. 6 km S of Meningie, Coorong road, 35°44’38”S, 139°20’03”E, *S.W.L. Jacobs 9774 & M.E. Barkworth*, 09 Dec. 2007 (HAL0110794, duplicates: AD228914, BA, NSW594287, UTC00252322); LR989258; LR989184; –; LEP ill.; **(3)** Australia, SA, South-eastern, c. 40 km N of Kingston SE, Salt Creek Road, 36°29’33”S, 139°49’29”E, *S.W.L. Jacobs 9689*, 25 Oct. 2007 (HAL0110804, duplicates: AD229032, B, NSW831774, TCD, UTC00252157); LR989259; LR989186; –; LEP. ***A. stuposa*** (Hughes) S.W.L.Jacobs & J.Everett: **(1)** Australia, VIC, East Gippsland, Swan Reach, Mettung Road, 37°49’29”S, 147°51’48”E, *S.W.L. Jacobs 9711*, 31 Oct. 2007 (HAL0110803, duplicates: MEL2330236A, NSW831782, UTC00252156); LR989260; LR989187; (A) LR989109, (B) LR989110, (C) LR989111; LEP ill.; **(2)** LN832450*; –; –. ***A. tenuifolia*** (Steud.) S.W.L.Jacobs & J.Everett: –; EU489104**; –. ***A. trichophylla*** (Benth.) S.W.L.Jacobs & J.Everett: Australia, WA, 31°37’59”S, 117°42’23”E, 01 Oct. 2004, collected seed at MSB no. 363604 (K). Plants grown from seed at BG Halle by G. Winterfeld GW81 (HAL0137635); LN832460*; LN832428*; (A) LR989112, (B) LR989113. ***A. tuckeri*** (F.Muell) S.W.L.Jacobs & Everett: **(1)** Australia, NSW, North Western Plains, Hermidale, to SE of village, 31°32’46”S, 146°43’43”E, *S.W.L. Jacobs 9810 & M.E. Barkworth*, 13 Dec. 2007 (HAL0107514, duplicates: NSW594354, UTC); LR989261; LR989188; –; LEP ill.; **(2)** Australia, NSW, South Western Plains, c. 3 km NE of Gilgunnia, Nymagee road, 32°23’51”S, 146°2’48”E, *S.W.L. Jacobs 9807 & M.E. Barkworth*, 13 Dec. 2007 (NSW594348); LR989262; LR989189; –; LEP. ***A. variabilis*** (Hughes) S.W.L.Jacobs & J.Everett: Australia, WA, Eyre, c. 40 km S of Ravensthorpe, Hopetoun road,

33°52’26”S, 120°09’47”E, *S.W.L. Jacobs 9745 & M.E. Barkworth*, 02 Dec. 2007 (HAL0107542, duplicates: B, NSW832526, PERTH8500258, TCD, UTC); LR989263; LR989190; –; LEP ill. ***A. velutina*** (Vickery, S.W.L.Jacobs & J.Everett) S.W.L.Jacobs & J.Everett: Australia, SA, Nullarbor, c. 135 km E of Border Village, Nullarbor road, 31°35’2”S, 130°22’21”E, *S.W.L. Jacobs 9758 & M.E. Barkworth*, 05 Dec. 2007 (AD229035, NSW832523); LR989264; LR989191; –. ***A. verticillata*** (Nees ex Spreng.) S.W.L.Jacobs & J.Everett: **(1)** Australia, QLD, 28°50’7”S, 151°38’57”E, *T. Tyson-Doneley s.n.*, 23 Mar. 2006, collected seed at MSB no. 332235 (K). Plants grown from seed at BG Halle by G. Winterfeld GW71 (HAL0137625); LN832452*; LN832420*;(A) LR989114, (B) LR989115; **(2)** Australia, NSW, Central Western Slopes, c, 36 km of Wellington, Molong road, 32°50’57”S, 148°54’53”E, *S.W.L. Jacobs 9811 & M.E. Barkworth*, 13 Dec. 2007 (HAL0107513, duplicates: BA, NSW594355, UTC); LR989265; LR989192; –; LEP ill. ***A. vickeryana*** (J.Everett & S.W.L.Jacobs) S.W.L.Jacobs & J.Everett; morph. ***A. wakoolica*** (Vickery, S.W.L.Jacobs & J.Everett) S.W.L.Jacobs & J.Everett: –; JF769087**; –. ***Bromus erectus*** Huds.: AM234570*; FM179394*; –. ***B. inermis*** Leyss.: –; –; EU366392**. ***Celtica gigantea*** (Link) F.M.Vázquez & Barkworth: FN434281*; FN434544*; –. ***Henrardia persica*** C.E.Hubb.: –; –; GQ228396**. ***Hordeum chilense*** Roem. & Schult.: –; –; DQ497805**. ***H. vulgare*** L.: KC912687**; FN556796**; AF343509**. ***Macrochloa tenacissima*** (L.) Kunth: Spain, *R. Piwowarczyk s.n.* (KRA); LEP ill. ***Nassella neesiana*** (Trin. & Rupr.) Barkworth: **(1)** Australia, NSW, Southern Tablelands, c. 18 km E of Bungonia, Goulburn Road, 34°48’28”S, 149°46’41”E, *S.W.L. Jacobs 9729 & M.E. Barkworth*, 23 Nov. 2007 (HAL0110790, duplicates: NSW832484, TCD, UTC); LR989266; LR989195; –; **(2)** Italy, Roma, *Moraldo s.n.* (KRA); LEP ill. ***N. trichotoma*** (Nees) Hack. & Arechav.: Australia, NSW, Southern Tablelands, c. 5 km S of Marulan, Prarie Oak Road on Bungonia-Marulan Road, 34°45’17”S, 149°58’17”E, *S.W.L. Jacobs 9727 & M.E. Barkworth*, 23 Nov. 2007 (HAL0110791, duplicates: B, BA, K, NSW832483, TCD, UTC); LR989267; LR989196; LR989120. ***Neotrinia splendens*** (Trin.) M.Nobis, P.D.Gudkova & A.Nowak: **(1)** FN434208*; FN434477*; –; **(2)** Tajikistan, *Yu. Gusev s.n.*, s.d. (LE); LEP ill. ***Oloptum miliaceum*** (L.) Röser & Hamasha: LN832471*, AM234597*; LN832439*, FM179427*; –. ***Orthoraphium roylei*** Nees: *J.F. Duthie 3558* (LE); LEP ill. ***Ptilagrostis concinna*** (Hook.f.) Roshev.: India, Ladakh, *L. Klimes s.n.* (KRA); LEP ill. ***P. mongholica*** (Turcz. ex Trin) Griseb.: Mongolia, *A. Pacyna s.n.* (KRA); LEP ill. ***Secale sylvestre*** Host: AM234581*; FM179434*; –. ***Stipa capillata*** L.: Germany, -Saxony-Anhalt, plants grown in BG Halle, *M. Röser 11083*, 16 June 2011 (HAL); –; –; (A) LR989121, (B) LR989122. ***S. drobovii*** (Tzvel.) Czerep.: Tajikistan, *M. Nobis s.n.* (KRA); LEP ill. ***S. kirghisorum*** P.A.Smirn.: Tajikistan, *M. Nobis s.n.* (KRA); LEP ill. ***S. pennata*** subsp. ***eriocaulis*** (Borbás) Martinovský & Skalický: –; FM179438*; –. ***S. pennata*** L. subsp. ***zalesskii*** (Wilensky) Freitag: FN434317*; –; –. ***S. tirsa*** Steven: **(1)** Germany, Saxony-Anhalt, plants grown in BG Halle, *M. Röser 11084*, 16 June 2011 (HAL): FN434350*; FN434605*; (A) LR989123, (B) LR989124, (C) LR989125; **(2)** Georgia, *M. Nobis s.n.* (KRA); LEP ill. ***Stipellula capensis*** (Thunb.) Röser & Hamasha: **(1)** FN434257*; FN434522*; –; **(2)** Spain, *R. Piwowarczyk s.n.* (KRA); LEP ill.
