# Supplementary material for: Molecular Phylogenetics and Micromorphology of Australasian Stipeae (Poaceae, Subfamily Pooideae), and the Interrelation of Whole-Genome Duplication and Evolutionary Radiations in This Grass Tribe
Source: Front Plant Sci. 2021 Jan 22;11:630788. doi: 10.3389/fpls.2020.630788 (PMC7862344; doi:10.3389/fpls.2020.630788)
Supplement: Supplementary Appendix 2 — Annotated list of chromosome number reports for taxa of Stipeae. Underlined are the most frequent chromosome numbers in case of different counting, if this was stated in the original publications or if concluded from this survey (mostly for the genera). Original publications we did not examine are identified as such in the list of references below. [file Data_Sheet_2.pdf]

Tkach, N., Nobis, M., Schneider, J., Becher, H., Winterfeld, G., Jacobs, S.W.L., Röser, M. 2021. Molecular phylogenetics and micromorphology of Australasian Stipeae (Poaceae, subfamily Pooideae), and the interrelation of whole-genome duplication and evolutionary radiations in this grass tribe. *Front. Plant Sci.* 11:630788. doi: 10.3389/fpls.2020.630788

Supplementary Appendix 2. Annotated list of chromosome number reports for taxa of Stipeae. Underlined are the most frequent chromosome numbers in case of different countings, if this was stated in the original publications or if concluded from this survey (mostly for the genera). Original publications we did not examine are identified as such in the list of references below.

Achnatherum P.Beauv.: **2*n* = 18**, **20**, **22**, **24**, **28**, **42**, **44**, **46**, **48**. — ***A*. *brandisii*** (Mez) Z.L.Wu: ***n* = 12**: Kumari & al. (2019) as *Stipa brandisii* Mez. ***A*. *bromoides*** (L.) P.Beauv.: **2*n* = 18**: Petrova (1968); **2*n* = 24**: Strid & Andersson (1985) as *Stipa bromoides* (L.) Dörfl.; Ghukasyan (2004); **2*n* = 28**: Löve & Löve (1961) citing an unpubl. count of Uihelyi; Vázquez & Devesa (1996) as *S. bromoides* (L.) Dörfl. ***A*. *calamagrostis*** (L.) P.Beauv.: **2*n* = 22 + 0–2B**: Strid (1983); **2*n* = 24**: Gervais (1966); Löve & Löve (1974); Strobl & Wittmann (1985); Kožuharov & Petrova (1991). ***A*. *caragana*** (Trin.) Nevski: **2*n* = 24**: Sokolovskaya & Probatova (1978). ***A*. *confusum*** (Litv.) Tzvelev: **2*n* = 24**: Sokolovskaya & Probatova (1977); Probatova & Seledets (2008); Probatova & al. (2008, 2015). ***A*. *dregeanum*** (Steud.) Röser, Tkach & M.Nobis: **2*n* = 48**: de Winter (1965) as *Stipa dregeana* Steud. ***A*. *jacquemontii*** (Jaub. & Spach) P.C.Kuo & S.L.Lu: ***n* = 10**: Kumar & al. (2018) as *Stipa jacquemontii* Jaub. & Spach; ***n* = 12**: Mehra & Sharma (1975a) as *S. jacquemontii*; ***n* = 21**, **22**: Gupta & al. (2014) as *S. jacquemontii*. ***A*. *paradoxum*** (L.) Banfi, Galasso & Bartolucci: **2*n* = 24**: Litardière (1950) as *Oryzopsis paradoxa* (L.) Nutt.; Löve & Kjellqvist (1973) as *O. paradoxa*. ***A*. *pekinense*** (Hance) Ohwi: **2*n* = 24**: Ono & Tateoka (1953) as *Stipa extremiorientalis* Hara; Tateoka (1954, 1967, 1986); Sokolovskaya & Probatova (1977) as *Achnatherum extremiorientale* (Hara) Keng; Korobkov & al. (2013) as *A. extremiorientale*. ***A*. *petriei*** (Buchanan) S.W.L.Jacobs & J.Everett: **2*n* = 42**: Murray & al. (2005). ***A*. *sibiricum*** (L.) Keng ex Tzvelev: ***n* = 12**, **2*n* = 24:** Johnson (1945) as *Stipa sibirica* (L.) Lam.; **2*n* = 22**, **23**: Měsíček & Soják (1972) as *S. sibirica*; **2*n* = 22**: Stepanov (1994) as *S. sibirica*; **2*n* = 24**: Avdulov (1928, 1931: 129) as *S. sibirica*; Myers (1947) citing an unpubl. count of R.M. Love as *S. sibirica*; Belyaeva & Siplivinsky (1977) as *S. sibirica*; Sokolovskaya & Probatova (1978); Gusik & Levkovskiy (1979) as *S. sibirica*; Probatova & Sokolovskaya (1980); Rudyka (1990); Probatova & al. (2009, 2012, 2015, 2016a); Chepinoga & al. (2012); Winterfeld & al. (2015). ***A*. *turcomanicum*** (Roshev.) Tzvelev: **2*n* = 24**: Chopanov & Yurtzev (1976) as *Stipa litwinowiana* P.A.Smirn. ex Pavlov & Lipsch. ***A*. *virescens*** (Trin.) Banfi, Galasso & Bartolucci: **2*n* = 24**: Avdulov (1931: 131) as *Oryzopsis virescens* (Trin.) Beck; Májovský 1974 as *O. virescens*.

Aciachne Benth.: **2*n* = 22**. — ***A*. *pulvinata*** Benth.: **2*n* = 22**: Reeder & Reeder (1968).

Amelichloa Arriaga & Barkworth: **2*n* = 40**, **44**, **44–46**, **46**. — ***A*. *brachychaeta*** (Godr.) Arriaga & Barkworth: **2*n* = 40**: Saura (1943) as *Stipa brachychaeta* Godr.; **2*n* = 40**, **44**: Saura (1948) as *S. brachychaeta*; **2*n* = 44**: Parodi (1946) citing an unpubl. count of O. Nuñez as *S. brachychaeta*; Bowden & Senn (1962) as *S. brachychaeta*; **2*n* = 44–46**: Myers (1947) citing an unpubl. count of G.L. Stebbins as *S. brachychaeta*; **2*n* = 46**: González & al. (2017).

Anatherostipa (Hack. ex Kuntze) Peñail.: **2*n* = 22**. — ***A*. *hans-meyeri*** (Pilg.) Peñail.: ***n* = 11** Davidse & Pohl (1978) as *Stipa hans-meyeri* Pilg.

Anemanthele Veldkamp: **2*n* = 40**, **42**, **44**, **46**. — ***A*. *lessoniana*** (Steud.) Veldkamp: **2*n* = 40–44**: Dawson & Beuzenberg (2000); Edgar & Connor (2000) citing an unpubl. count of M.I. Dawson; **2*n* = 42**, **44**, **46**: Winterfeld & al. (2015).

Austrostipa **S.W.L.Jacobs & J.Everett**: **2*n* = 42**, **44**, **64**, **66**, **68**, **70**. — ***A*. *acrociliata*** (Reader) S.W.L.Jacobs & J.Everett: **2*n* = 44**: Winterfeld & al. (2015). ***A*. *blackii*** (C.E.Hubb.)

S.W.L.Jacobs & J.Everett: **2*n* = 44**: Winterfeld & al. (2015). ***A*. *breviglumis*** (J.M.Black) S.W.L.Jacobs & J.Everett: **2*n* = 44**: Winterfeld & al. (2015). ***A*. *campylachne*** (Nees) S.W.L.Jacobs & J.Everett: **2*n* = 66**: Winterfeld & al. (2015). ***A*. *curticoma*** (Vickery) S.W.L.Jacobs & J.Everett: **2*n* = 66**: Winterfeld & al. (2015). ***A*. *drummondii*** (Steud.) S.W.L.Jacobs & J.Everett: **2*n* = 44**: Winterfeld & al. (2015). ***A*. *elegantissima*** (Labill.) S.W.L.Jacobs & J.Everett: **2*n* = 66**: Winterfeld & al. (2015). ***A*. *exilis*** (Vickery) S.W.L.Jacobs & J.Everett: **2*n* = 44**: Winterfeld & al. (2015). ***A*. *flavescens*** (Labill.) S.W.L.Jacobs & J.Everett: **2*n* = 44**: Winterfeld & al. (2015). ***A*. *geoffreyi*** S.W.L.Jacobs & J.Everett: **2*n* = 44**: Winterfeld & al. (2015). ***A*. *hemipogon*** (Benth.) S.W.L.Jacobs & J.Everett. **2*n* = 66**, **70**: Winterfeld & al. (2015). ***A*. *mollis*** (R.Br.) S.W.L.Jacobs & J.Everett: **2*n* = 64**, **66**: Winterfeld & al. (2015). ***A*. *nodosa*** (S.T.Blake) S.W.L.Jacobs & J.Everett: **2*n* = 44**: Winterfeld & al. (2015). ***A*. *nullanulla*** (S.W.L.Jacobs & J.Everett) S.W.L.Jacobs & J.Everett: **2*n* = 44**: Winterfeld & al. (2015). ***A*. *oligostachya*** (Hughes) S.W.L.Jacobs & J.Everett: **2*n* = 44**: Winterfeld & al. (2015). ***A*. *pilata*** (S.W.L.Jacobs & J.Everett) S.W.L.Jacobs & J.Everett: **2*n* = 44**: Winterfeld & al. (2015). ***A*. *platychaeta*** (Hughes) S.W.L.Jacobs & J.Everett: **2*n* = 44**: Winterfeld & al. (2015). ***A*. *ramosissima*** (Trin.) S.W.L.Jacobs & J.Everett: **2*n* = 44**: Winterfeld & al. (2015). ***A*. *scabra*** (Lindl.) S.W.L.Jacobs & J.Everett: **2*n* = 42**, **44**: Winterfeld & al. (2015). ***A*. *scabra*** (Lindl.) S.W.L.Jacobs & J.Everett subsp. ***falcata*** (Hughes) S.W.L.Jacobs & J.Everett: **2*n* = 44**: Winterfeld & al. (2015). ***A*. *scabra*** (Lindl.) S.W.L.Jacobs & J.Everett subsp. ***scabra***: **2*n* = 60**, **62**: Winterfeld & al. (2015). ***A*. *semibarbata*** (R.Br.) S.W.L.Jacobs & J.Everett: **2*n* = 65**, **66**, **68**, **70**: Winterfeld & al. (2015). ***A*. *stipoides*** (Hook.f.) S.W.L.Jacobs & J.Everett: **2*n* = 44**: Murray & al. (2005); Winterfeld & al. (2015). ***A*. *stuposa*** (Hughes) S.W.L.Jacobs & J.Everett: **2*n* = 66**: Winterfeld & al. (2015). ***A*. *trichophylla*** (Benth.) S.W.L.Jacobs & J.Everett: **2*n* = 44**: Winterfeld & al. (2015). ***A*. *verticillata*** (Nees ex Spreng.) S.W.L.Jacobs & J.Everett: **2*n* = 44**: Winterfeld & al. (2015).

Barkworthia Romasch., P.M.Peterson & Soreng: **2*n* = 40**. — ***B*. *stillmanii*** (Bol.) Romasch., P.M.Peterson & Soreng: **2*n* = 40**: Myers (1947) citing an unpubl. count of G.L. Stebbins as *Stipa stillmanii* Bol.

Celtica F.M.Vázquez & Barkworth: **2*n* = 84**, **96**. — ***C*. *gigantea*** (Link) F.M.Vázquez & Barkworth: ***n* = 48**: Devesa & al. (1991) as *Stipa gigantea* Link; Gallego & Talavera (1994) as *S. gigantea*; **2*n* = 84**: Winterfeld (2006) as *S. gigantea*; **2*n* = 96**: Fernandes & Queirós (1969) as *S. gigantea*; Queirós (1973) as *S. gigantea*; Queirós (1974) as *S. gigantea*. ***C*. *gigantea*** subsp. ***gigantea***: **2*n* = 96**: Vázquez & Devesa (1996) as *S. gigantea* subsp. *gigantea*. ***C*. *gigantea*** subsp. ***donyanae*** (F.M.Vázquez & Devesa) F.M.Vázquez & Barkworth: **2*n* = 96**: Vázquez & Devesa (1996) as *S. gigantea* subsp. *donyanae* F.M.Vázquez & Devesa.

Eriocoma Nutt.: **2*n* = 32**, **34**, **36**, **40**, **44**, **48**, **64**, **66**, **68**, **70**. — ***E*. *contracta*** (B.L.Johnson) Romasch.: ***n* = 24**: Reeder (1977) as *Oryzopsis contracta* (B.L.Johnson) Schechter; **2*n* = 48**: Shechter & Johnson (1966) as *O. contracta*. ***E*. *coronata*** (Thurb.) Romasch.: **2*n* = 40**: Stebbins & Love (1941) as *Stipa coronata* Thurb.; Myers (1947) citing an unpubl. count of R.M. Love as *S. coronata*; Reeder (1984) as *S. coronata*. ***E*. *curvifolia*** (Swallen) Romasch.: ***n* = 22**: Hatch & Bearden (1983) as *Stipa curvifolia* Swallen. ***E*. *hendersonii*** (Vasey) Romasch.: ***n* = 17**: Hitchcock & Spellenberg (1968) as *Oryzopsis hendersonii* Vasey; ***n* = 17**, **2*n* = 34**: Spellenberg (1968) as *O. hendersonii*. ***E*. *hymenoides*** (Roem. & Schult.) Rydb.: ***n* = 24**: Reeder (1977) as *Oryzopsis hymenoides* (Roem. & Schult.) Ricker ex Piper; ***n* = 24**, **2*n* = 48**: Johnson (1945, 1960, 1962a) as *O. hymenoides*; **2*n* = 48**: Stebbins & Love (1941) as *O. hymenoides*; Johnson & Rogler (1943) as *O. hymenoides*; Johnson (1960) as *O. hymenoides*; Löve & Löve (1981) as *O. hymenoides*; **2*n* = 48 + 0–5B**: Johnson (1963) as *O. hymenoides*. ***E*. *latiglumis*** (Swallen) Romasch.: ***n* = 35**: Pohl (1954) as *Stipa latiglumis* Swallen representing an allopolyploid hybrid between *S. lettermanii* and *S. elmeri*. ***E*. *lemmonii*** (Vasey) Romasch.: ***n* = 17**: Pohl (1954) as *Stipa lemmonii* (Vasey) Scribn.; Reeder (1977) as *S. lemmonii*; **2*n* = 34**: Myers (1947) citing an unpubl. count of R.M. Love as *S. lemmonii*; ***n* = 18**, **2*n* = 36**: Stebbins &

Love (1941) as *S. lemmonii*; Johnson (1962a) as *S. columbiana* Macoun; **2*n* = 44**: Nielsen (1939) as *S. columbiana*. ***E. lemmonii*** subsp. ***pubescens*** (Crampton) Romasch.: **2*n* = 34**: Stebbins & Love (1941) as *Stipa lemmonii* var. *jonesii* Scribn. ***E. lettermanii*** (Vasey) Romasch.: ***n* = 16**, **2*n* = 32**: Johnson (1962a) as *S. lettermanii*; **2*n* = 32**: Dedecca (1954) as *S. lettermanii* and commenting that this plant with 2*n* = 32 was true *S. lettermanii*, whereas the plant of Stebbins & Love (1941) with 2*n* = 66 belonged to an undescribed polyploid; **2*n* = 66**: Stebbins & Love (1941) as *Stipa lettermanii* Vasey var.; **2*n* = 68**: Myers (1947) citing an unpubl. count of R.M. Love as *S. lettermanii*. ***E. nevadensis*** (B.L.Johnson) Romasch.: ***n* = 34**, **2*n* = 68**: Johnson (1962a) as *Stipa nevadensis* B.L.Johnson and representing an amphidiploid hybrid between *S. lettermanii* and *S. occidentalis* or *S. elmeri*; **2*n* = 68**: Johnson (1962b) as *S. nevadensis*. ***E. occidentalis*** (Thurb. ex S.Watson) Romasch.: **2*n* = 36**: Stebbins & Love (1941) as *Stipa occidentalis* Thurb. ***E. occidentalis*** subsp. ***californica*** (Merr. & Burtt Davy) Romasch.: ***n* = 18**: Reeder (1977) as *Stipa californica* Merr. & Burtt Davy; ***n* = 18**, **2*n* = 36**: Stebbins & Love (1941) as *S. californica*; Johnson (1962a) as *S. occidentalis* Thurb. ***E. occidentalis*** subsp. ***pubescens*** (Vasey) Romasch.: ***n* = 18**: Pohl (1954) as *Stipa elmeri* Piper & Brodie ex Scribn.; ***n* = 18**, **2*n* = 36**: Stebbins & Love (1941) as *S. elmeri*; Johnson (1945, 1962a) as *S. elmeri*. ***E. pinetorum*** (M.E.Jones) Romasch.: ***n* = 16**, **2*n* = 32**: Johnson (1963) as *Stipa pinetorum* M.E.Jones; **2*n* = 32**: both Johnson (1945) and Myers (1947) and citing an unpubl. count of G.L. Stebbins as *S. pinetorum*. ***E. richardsonii*** (Link) Romasch.: **2*n* = 44**: Löve & Löve (1981) as *Stipa richardsonii* Link. ***E. robusta*** (Vasey) Romasch.: **2*n* = 64**: Myers (1947) citing an unpubl. count of R.M. Love as *Stipa robusta* (Vasey) Scribn. ***E. scribneri*** (Vasey) Romasch.: **2*n* = 40**: Tateoka (1955) as *Stipa scribneri* Vasey. ***E. swallenii*** (C.L.Hitchc. & Spellenb.) Romasch.: ***n* = 17**, **2*n* = 34**: Hitchcock & Spellenberg (1968) as *Oryzopsis swallenii* C.L.Hitchc. & Spellenb. ***E. thurberiana*** (Piper) Romasch.: ***n* = 17**, **2*n* = 34**: Stebbins & Love (1941) as *Stipa thurberiana* Piper. ***E. webberi*** Thurb.: ***n* = 16**, **2*n* = 32**: Johnson (1945) as *Stipa webberi* (Thurb.) B.L.Johnson.

Hesperostipa (M.K.Elias) Barkworth: **2*n* = 38, 44, 46**. — ***H. comata*** (Trin. & Rupr.) Barkworth: ***n* = 19**, **22**: Reeder (1977) as *Stipa comata* Trin. & Rupr.; **2*n* = 46**: Myers (1947) citing an unpubl. count of R.M. Love as *S. comata*. ***H. comata*** subsp. ***comata***: **2*n* = 44**: Bowden (1960) as *S. comata*; Löve & Löve (1981) as *S. comata*. ***H. comata*** subsp. ***intermedia*** (Scribn. & Tweedy) Barkworth: **2*n* = 44–46**: Stebbins & Love (1941) as *Stipa comata* var. *intermedia* Scribn. & Tweedy. ***H. curtiseta*** (Hitchc.) Barkworth: **2*n* = 44**: Löve & Löve (1981) as *Stipa curtiseta* (Hitchc.) Barkworth. ***H. neomexicana*** (Thurb.) Barkworth: ***n* = 22**: Gould (1968) as *Stipa neomexicana* (Thurb.) Scribn.; **2*n* = 44**: Myers (1947) citing an unpubl. count of R.M. Love as *S. neomexicana*. ***H. spartea*** (Trin.) Barkworth: **2*n* = 44**: Tateoka (1955) as *Stipa spartea* Trin.; Löve & Löve (1981) as *S. spartea*; **2*n* = 46**: Myers (1947) citing an unpubl. count of R.M. Love as *S. spartea*.

Jarava Ruiz & Pav.: **2*n* = 36, 40, 42, 44, 66**. — ***J. filifolia*** (Nees) Ciald.: **2*n* = 36**: González & al. (2017). ***J. ichu*** Ruiz & Pav.: ***n* = 20**: Pohl & Davidse (1971) as *Stipa ichu* (Ruiz & Pav.) Kunth; Davidse & Pohl (1974) as *S. ichu*; ***n* = 20**, **ca. 20**: Gould (1966) as *S. ichu*; **2*n* = 42**: Bowden & Senn (1962) as *S. ichu*; **2*n* = 44**: Covas (1945) as *Stipa gynerioides* Phil.; Parodi (1946) citing a pers. commun. of F. Saura as *S. ichu*; Saura (1948) as *S. ichu*; Tateoka (1962) as *S. ichu*. ***J. neaei*** (Nees ex Steud.) Peñail.: **2*n* = 66**: Covas & Bocklet (1945) as *Stipa neaei* Nees ex Steud. ***J. plumosa*** (Spreng.) S.W.L.Jacobs & J.Everett: **2*n* = 40**: Bowden & Senn (1962) as *Stipa papposa* Nees; González & al. (2017); **2*n* = 44**: Avdulov (1928, 1931: 129) as *S. papposa* Nees; Lorenzo-Andreu (1953) as *S. papposa* Nees; Parodi (1946) as *S. papposa*. ***J. plumosula*** (Nees ex Steud.) F.Rojas: **2*n* = 44**: Covas & Bocklet (1945) as *Stipa plumosa* Trin. & Rupr.

Lorenzochloa Reeder & C.Reeder: **2*n* = 22**. — ***L. erectifolia*** (Swallen) Reeder & C.Reeder: **2*n* = 22**: Reeder & Reeder (1968) as *Parodiella erectifolia* (Swallen) Reeder & C.Reeder.

Macrochloa Kunth; **2*n* = 24, 40, 66, 72**. — ***M. tenacissima*** (L.) Kunth: ***n* = 33**: Ferchichi & al. (1994) as *Stipa tenacissima* L.; **2*n* = 24**: Labadie (1979a,b) as *S. tenacissima*; **2*n* = 40**: Fernandes

& Queirós (1969) as *S. tenacissima*; **2*n* = 66**: Lungeanu (1980) as *S. tenacissima*; ***n* = 36**: Faruqi & al. (1987) as *S. tenacissima*.

Nassella (Trin.) É.Desv.: **2*n* = 26**, **28**, **30**, **32**, **34**, **36**, **38**, **40, 42**, **56**, **58**, **60**, **64**, **66**, **70**, **82**, **88**. — ***N. cernua*** (Stebbins & Love) Barkworth: ***n* = 35**: Love (1954) as *Stipa cernua* Stebbins & Love; ***n* = 35**, **2*n* = 70**: Stebbins & Love (1941) as *S. cernua*; **2*n* = 70**: Love (1946) as *S. cernua*. ***N. charruana*** (Arechav.) Barkworth: **2*n* = 36**: Bowden & Senn (1962) as *Stipa charruana* Arechav; González & al. (2017). ***N. chilensis*** (Trin.) É.Desv.: **2*n* = 42**: Bowden & Senn (1962). ***N. gigantea*** (Steud.) Muñoz-Schick: **2*n* = 42**: Bowden & Senn (1962) as *N. exserta* Phil. ***N. hyalina*** (Nees) Barkworth: **2*n* = 34**: Bowden & Senn (1962) as *Stipa hyalina* Nees; González & al. (2017). ***N. lachnophylla*** (Trin.) Barkworth: **2*n* = 66**: Bowden & Senn (1962) as *Stipa laxa* É.Desv. ***N. lepida*** (Hitchc.) Barkworth: ***n* = 17**: Love (1954) as *Stipa lepida* Hitchc.; ***n* = 17**, **2*n* = 34**: Stebbins & Love (1941) as *S. lepida*; **2*n* = 34**: Love (1946) as *S. lepida*; **2*n* = 46**: Reeder (1967) as *S. lepida*. ***N. leptocoronata*** (Roseng. & B.R.Arrill.) Barkworth: **2*n* = 26**: González & al. (2017). ***N. leucotricha*** (Trin. & Rupr.) R.W.Pohl: ***n* = 13**: Brown (1949) as *Stipa leucotricha* Trin. & Rupr.; ***n* = 14**: Gould (1958) as *S. leucotricha*; Hatch (1980) as *S. leucotricha*; **2*n* = 26**: Brown (1951) as *S. leucotricha*; **2*n* = 28**: Myers (1947) citing an unpubl. count of R.M. Love as *S. leucotricha*. ***N. longiglumis*** (Phil.) Barkworth: **2*n* = 30**: González & al. (2017). ***N. megapotamia*** (Spreng. ex Trin.) Barkworth: **2*n* = 34**: Myers (1947) citing an unpubl. count of G.L. Stebbins as *Stipa megapotamica* Spreng. ex Trin. & Rupr.; González & al. (2017). ***N. melanosperma*** (J.Presl) Barkworth: **2*n* = 36**: González & al. (2017). ***N. mucronata*** (Kunth) R.W.Pohl: **2*n* = 36**: Myers (1947) citing an unpubl. count of R.M. Love as *Stipa mucronata* Kunth; **2*n* = 64**: Bowden & Senn (1962) as *S. mucronata*. ***N. neesiana*** (Trin. & Rupr.) Barkworth: **2*n* = 28**: Myers (1947) citing an unpubl. count of G.L. Stebbins as *Stipa neesiana* Trin. & Rupr.; Brown (1949) as *S. neesiana*; Bowden & Senn (1962) as *S. neesiana*; González & al. (2017). ***N. pauciciliata*** (Roseng. & Izag.) Barkworth: **2*n* = 34**: González & al. (2017). ***N. philippii*** (Steud.) Barkworth: **2*n* = 36**: Myers (1947) citing an unpubl. count of G.L. Stebbins as *Stipa philippii* Steud.; González & al. (2017). ***N. poeppigiana*** (Trin.& Rupr.) Barkworth: **2*n* = 34**: González & al. (2017). ***N. pulchra*** (Hitchc.) Barkworth: **2*n* = 55**, **56**, **58**, **60**: Winterfeld & al. (2015); ***n* = 32**: Love (1954) as *Stipa pulchra* Hitchc.; ***n* = 32**, **2*n* = 64**: Stebbins & Love (1941) as *S. pulchra*; **2*n* = 64**: Love (1946) as *S. pulchra*; **2*n* = 66**: Nielsen (1939) as *S. pulchra*. ***N. tenuissima*** (Trin.) Barkworth: **2*n* = 32**: Brown (1951) as *Stipa tenuissima* Trin.; **2*n* = 40**: Bowden & Senn (1962) as *S. tenuissima*. ***N. trichotoma*** (Nees) Hack. ex Arechav.: **2*n* = 36**: Bowden & Senn (1962); González & al. (2017); **2*n* = 38**: Avdulov (1928) as *Urachne trichotoma* (Nees) Trin. fide Fedorov (1969); **2*n* = 40**: Parodi (1946) as *Stipa trichotoma* Nees. ***N. viridula*** (Trin.) Barkworth: ***n* = 41**, **2*n* = 82**: Johnson & Rogler (1943) as *Stipa viridula* Trin.; **2*n* = 88**: Löve & Löve (1981) as *S. viridula*.

Neotrinia (Tzvelev) M.Nobis, P.D.Gudkova & A.Nowak. **2*n* = 44**, **46**, **48**. — *N. splendens* (Trin.) M.Nobis, P.D.Gudkova & A.Nowak: **2*n* = 42**: Sokolovskaya & Strelkova (1948) as *Lasiagrostis splendens* (Trin.) Kunth but regarded by Probatova & Sokolovskaya (1980) as miscount; ***n* = 22**: Gupta & al. (2014) as *Stipa splendens* Trin.; **2*n* = 44**: Winterfeld & al. (2015) as *Achnatherum splendens* (Trin.) Nevski; ***n* = 23**: Gohil & Koul (1986) as *S. splendens*; **2*n* = 48**: Myers (1947) citing an unpubl. count of R.M. Love as *S. splendens* Trin.; **2*n* = 48**: Probatova & Sokolovskaya (1980) as *A. splendens*; **2*n* = ca**. **48**: Probatova & Seledets (2008) as *A. splendens*.

Oloptum Röser & Hamasha: **2*n* = 24**, **36**. — ***O. miliaceum*** (L.) Röser & Hamasha: ***n* = 12**: Faruqi & al. (1987) as *Pipthaterum miliaceum* (L.) Cosson; Devesa & al. (1991) as *P. miliaceum*; ***n* = 12**, **2*n* = 24**: Johnson (1945) as *Oryzopsis miliacea* (L.) Benth. & Hook.f. ex Asch. & Schweinf.; **2*n* = 24**: Avdulov (1928, 1931: 131) as *O. miliacea*; Tateoka (1957) as *O. miliacea*; Fernandes & Queirós (1969) as *O. miliacea*; Luque & Diaz Lifante (1991) as *P. miliaceum*; Verlaque & al. (1997) as *P. miliaceum*; Winterfeld & al. (2015); ***n* = 18**: Ferchichi & al. (1994) as *O. miliacea* and considering it a triploid plant.

Ortachne Nees: **no chromosome counts available**.

Orthoraphium Nees: **no chromosome counts available**.

Oryzopsis Michx.: **2*n* = 46**, **48**: — ***O***. ***asperifolia*** Michx.: **2*n* = 46**: Johnson (1945); **2*n* = 48**: Bowden (1960); Löve & Löve (1981).

Pappostipa (Speg.) Romasch., P.M.Peterson & Soreng: **2*n* = 42**, **60**, **64**, **66**, **68**, **ca**. **74**. — ***P***. ***humilis*** (Cav.) Romasch.: **2*n* = 42(–44)**: Myers (1947) citing an unpubl. count of G.L. Stebbins as *Stipa humilis* Cav.; **2*n* = 66**: Covas & Bocklet (1945) as *S. humilis*.

P. ***speciosa*** (Trin. & Rupr.) Romasch: **2*n* = 60**: Delay (1950) as *Stipa speciosa* Trin. & Rupr.; Stebbins & Love (1941) as *S. speciosa*; ***n* = 32**, **2*n* = 64**: Johnson (1960, 1962a) as *S. speciosa*; **2*n* = 66**: Covas & Bocklet (1945) as *S. speciosa* var. *macrochaeta* Parodi; **2*n* = 68**: Myers (1947) citing an unpubl. count of R.M. Love as *S. speciosa*; ***n* = ca**. **37**: Gould (1968) as *S. speciosa*.

Patis Ohwi: **2*n* = 46**, **48**. — ***P***. ***coreana*** (Honda) Ohwi: **2*n* = 46**: Tateoka (1986) as *Orthoraphium coreanum* var. *kengii* (Ohwi) Ohwi. ***P***. ***racemosa*** (Sm.) Romasch., P.M.Peterson & Soreng: **2*n* = 46**: Johnson (1945) as *Oryzopsis racemosa* (Sm.) Ricker ex Hitchc.; **2*n* = 48**: Bowden (1960) as *O. racemosa*.

Piptatheropsis Romasch., P.M.Peterson & Soreng: **2*n* = 20**, **22**, **24**. — ***P***. ***canadensis*** (Poir.) Romasch., P.M.Peterson & Soreng: **2*n* = 22**: Spellenberg (1970) as *Oryzopsis canadensis* (Poir.) Torr., not Johnson (1945), erroneously cited by Curto & Henderson (1998). ***P***. ***exigua*** (Thurb.) Romasch., P.M.Peterson & Soreng: **2*n* = 22**: Hitchcock & Spellenberg (1968) as *Oryzopsis exigua* Thurb. ***P***. ***micrantha*** (Trin. & Rupr.) Romasch., P.M.Peterson & Soreng: **2*n* = 22**: Johnson (1945) as *Oryzopsis micrantha* (Trin. & Rupr.) Thurb.; **2*n* = 24**: Löve & Löve (1981) as *O. micrantha*. ***P***. ***pungens*** (Torr. ex Spreng.) Romasch., P.M.Peterson & Soreng: **2*n* = 22**: Johnson (1945) as *Oryzopsis pungens* (Torr. ex Spreng.) Hitchc.; **2*n* = 24**: Bowden (1960) as *O. pungens*; Löve & Löve (1981) as *O. pungens*. ***P***. ***shoshoneana*** (Curto & Douglass M.Hend.) Romasch., P.M.Peterson & Soreng: ***n* = 10**, **2*n* = 20**: Curto & Henderson (1998) as *Stipa shoshoneana* Curto & Douglass M.Hend.

Piptatherum P.Beauv.: **2*n* = 20**, **22**, **24**, **26**. — ***P***. ***aequiglume*** (Duthie ex Hook.f.) Roshev.: ***n* = 12**: Mehra & Sharma (1975a) as *Oryzopsis aequiglumis* Duthie ex Hook.f. ***P***. ***coerulescens*** (Desf.) P.Beauv.: **2*n* = 24**: Kerguélen (1975) as *Oryzopsis coerulescens* (Desf.) Hack. ***P***. ***holciforme*** (M.Bieb.) Roem. & Schult.: **2*n* = 24**: Johnson (1945) as *Oryzopsis holciformis* (M.Bieb.) Hack.; Petrova (1968). ***P***. ***gracile*** Mez: ***n* = 12**, **2*n* = 24**: Moinuddin & al. (1994). ***P***. ***laterale*** (Munro ex Regel) Munro ex Nevski: ***n* = 12**: Mehra & Sharma (1975b, 1977) as *Oryzopsis lateralis* (Munro ex Regel) Stapf ex Hook.f.; Gupta & al. (2014) as *O. lateralis*; Singhal & al. (2014) as *O. lateralis*. ***P***. ***microcarpum*** (Pilg.) Tzvelev: ***n* = 12**: Kaur & al. (2011a,b); **2*n* = 24**: Podlech & Dieterle (1969) as *Oryzopsis microcarpa* Pilg. ***P***. ***molinioides*** Boiss.: ***n* = 11 + 1B**: Mehra & Sunder (1969) as *Oryzopsis molinioides* (Boiss.) Hack. ex Paulsen; ***P***. ***munroi*** (Stapf) Mez: **2*n* = 22**: Mehra & Sunder (1969) as *Oryzopsis munroi* Stapf; ***n* = 12**: Sharma & Sharma (1978, 1979) as *O. munroi*; Moinuddin & al. (1994); Singhal & al. (2014) as *O. munroi*. ***P***. ***songaricum*** (Trin. & Rupr.) Roshev. ex Nikitina subsp. ***songaricum***: **2*n* = 24**: Sokolovskaya & Probatova (1978). ***P***. ***vicarium*** (Grig.) Roshev. ex Nikitina: **2*n* = 20**: Chopanov & Yurtzev (1976); **2*n* = 24**, **26**: Sokolovskaya & Probatova (1978). ***P***. ***virescens*** (Trin.) Boiss.: **2*n* = 24**: Avdulov (1928, 1931: 131) as *Oryzopsis virescens* (Trin.) Beck; Májovský (1974) as *O. virescens*; Sokolovskaya & Probatova (1978); Kožuharov & Petrova (1991); Probatova & Seledets (2008); Marhold & al. (2007) as *O. virescens*.

Piptochaetium J.Presl: **2*n* = 22**, **42**, **44**, **ca**. **60**. — ***P***. ***avenaceum*** (L.) Parodi: ***n* = 11**: Gould (1958) as *Stipa avenacea* L.; Valencia & Costas (1968). ***P***. ***bicolor*** (Vahl) É.Desv.: **2*n* = 22**: Covas & Bocklet (1945); Parodi (1946) citing an unpubl. count of J.I. Valencia as *P. bicolor* var. *minor* (Speg.) Parodi; Valencia & Costas (1968) as *P. bicolor* var. *minor*; Parodi (1946) citing an unpubl. count of J.I. Valencia as *P. bicolor* var. *typicum* (Speg.) Parodi; Valencia & Costas (1968) as *P. bicolor* var. *typicum*. ***P***. ***brevicalyx*** (E.Fourn.) Ricker: ***n* = 11**: Gould (1965). ***P. confusum*** Parodi: **2*n* = 22**:

Valencia & Costas (1968). ***P. fimbriatum*** (Kunth) Hitchc.: ***n* = 21**: Gould (1965); Reeder (1968); **2*n* = 44**: Brown (1951: Fig. 11 and Table 1), on p. 296 apparently erroneously 2*n* = 22; Valencia & Costas (1968). ***P. hackelii*** (Arechav.) Parodi: **2*n* = 22**: Valencia & Costas (1968). ***P. lasianthum*** Griseb.: **2*n* = 22**: Parodi (1946); Parodi (1946) citing an unpubl. count of J.I. Valencia; Valencia & Costas (1968). ***P. montevidense*** (Spreng.) Parodi: **2*n* = 22**: Parodi (1946) citing an unpubl. count of J.I. Valencia; Valencia & Costas (1968). ***P. napostaense*** (Speg.) Hack.: **2*n* = 22**: Covas & Bocklet (1945). ***P. pringlei*** (Beal) Parodi: ***n* = 21**: Reeder (1977) as *Stipa pringlei* Scribn.; **2*n* = 42**: Myers (1947) citing an unpubl. count of R.M. Love as *S. pringlei*. ***P. ruprechtianum*** É. Desv.: **2*n* = 22**: Valencia & Costas (1968). ***P. stipoides*** (Trin. & Rupr.) Hack. ex Arechav.: **2*n* = 22**: Parodi (1946); Valencia & Costas (1968) as *P. stipoides* var. *chaetophorum* (Griseb.) Parodi; Valencia & Costas (1968) as *P. stipoides* var. *genuinum* Parodi; Parodi (1946) citing an unpubl. count of J.I. Valencia as *P. stipoides* var. *purpurascens* (Hack.) Parodi; Valencia & Costas (1968) as *P. stipoides* var. *purpurascens*; Parodi (1946) citing an unpubl. count of J.I. Valencia as *P. stipoides* var. *verruculosum* (Mez) Parodi; Valencia & Costas (1968) as *P. stipoides* var. *verruculosum*. ***P. uruguense*** Griseb.: **2*n* = 22**: Parodi (1946) citing an unpubl. count of J.I. Valencia; Valencia & Costas (1968). ***P. virescens*** (Kunth) Parodi: ***n* = ca. 30**: Gould (1966) as *Stipa virescens* Kunth and reporting disturbance of meiosis.

Psammochloa Hitchc.: **no chromosome counts available**.

Pseudoeriocoma Romasch., P.M.Peterson & Soreng: **2*n* = 44, 46**. — ***P. eminens*** (Cav.) Romasch.: ***n* = 22**: Gould (1965) as *Stipa eminens* Cav.; **2*n* = 44**: Reeder (1984) as *S. eminens*; ***n* = 23**: Gould (1966) as *S. eminens*; **2*n* = 46**: Myers (1947) citing an unpubl. count of R.M. Love as *S. eminens*; Brown (1951) as *S. eminens* Cav.

Ptilagrostiella Romasch., P.M.Peterson & Soreng: **2*n* = 22**. — ***P. kingii*** (Bol.) Romasch.: **2*n* = 22**: Johnson (1945) as *Oryzopsis kingii* (Bol.) Beal.

Ptilagrostis Griseb.: **2*n* = 22**. — ***P. contracta*** Z.S.Zhang & W.L.Chen: **2*n* = 22**: Zhang & al. (2017). ***P. junatovii*** Grubov: **2*n* = 22**: Krogulevich (1971, 1972). ***P. mongholica*** (Turcz. ex Trin.) Griseb.: **2*n* = 22**: Sokolovskaya & Strelkova (1948); Krogulevich (1971); Murin & al. (1984) as *Achnatherum mongholicum* (Turcz. ex Trin.) Ohwi; Tateoka (1986). ***P. mongholica*** subsp. ***mongholica***: **2*n* = 22**: Sokolovskaya & Probatova (1978); Probatova & Sokolovskaya (1980).

Stipa L.: **2*n* = 20, 32, 40, <u>44</u>, 48, 66**. — ***S. aktauensis*** Roshev.: **2*n* = 44**: Sokolovskaya & Probatova (1978). ***S. apertifolia*** Martinovský: **2*n* = 44**: Moraldo (1986) as *S. dasyvaginata* subsp. *apenninicola* Martinovský & Moraldo. ***S. arabica*** Trin. & Rupr.: ***n* = 22**: Sheidai & Attaei (2005). ***S. arabica*** subsp. ***caspia*** (K.Koch) Tzvelev: **2*n* = 44**: Sokolovskaya & Probatova (1978). ***S. baicalensis*** Roshev.: **2*n* = 48**: Gusik (1973); Gusik & Levkovskiy (1979); **2*n* = 44**: Probatova & al. (2006); Korobkov & al. (2013). ***S. balcanica*** (Martinovský) Kožuharov: **2*n* = 44**: Kožuharov & Petrova (1991). ***S. barbata*** Desf.: **2*n* = 44**: Vázquez & Devesa (1996). ***S. capillacea*** Keng: **2*n* = 44**: Gupta & al. (2014: Table 1) as *S. koelzii* R.R.Stewart but *n* = 11 on p. 1245 and in Fig. 36. ***S. capillata*** L.: **2*n* = 44**: Avdulov (1928, 1931: 129); Delay (1947); Sokolovskaya & Strelkova (1948); Tarnavschi (1948); Lorenzo-Andreu (1951: 201) and erroneously as "*S. ciliata* L." on p. 202; Lorenzo-Andreu (1953); Kožuharov & Kuzmanov (1968); Petrova (1968); Skalińska & al. (1968); Májovský (1974); Chopanov & Yurtzev (1976); Sokolovskaya & Probatova (1978); Gusik & Levkovskiy (1979); Měsíček & Javůrcová-Jarolímová (1992); Vázquez & Devesa (1996); Ghukasyan (2004); Probatova & al. (2009, 2011). ***S. caucasica*** Schmalh.: ***n* = 22**: Sheidai & Attaei (2005); **2*n* = 44**: Sokolovskaya & Strelkova (1939); Probatova & Seledets (2008); Sokolovskaya & Strelkova (1948) as *S. glareosa* P.A.Smirn.; Probatova & Sokolovskaya (1980) as *S. glareosa*; Erst & al. (2019) as *S. glareosa*. ***S. caucasica*** subsp. ***desertorum*** (Roshev.) TzveIev; **2*n* = 44**: Sokolovskaya & Probatova (1978). ***S. ehrenbergiana*** Trin. & Rupr.; ***n* = 22**: Sheidai & Attaei (2005). ***S. ×gegarkunii*** P.A.Smirn.: **2*n* = 44**: Ghukasyan (2004). ***S. hohenackeriana*** Trin. & Rupr.: ***n* = 22**: Sheidai & Attaei (2005). ***S. holosericea*** Trin.: ***n* = 22**: Sheidai & Attaei (2005); **2*n* = 44**: Chopanov & Yurtzev (1976), ascribed to subsp. *transcaucasica* (Grossh.)

Tzvelev by Tzvelev (1976); Strid & Andersson (1985) as *S. fontanesii* Parl.; Strid (1987). ***S. iberica*** Martinovský: **2*n* = 44**: Vázquez & Devesa (1996); Vázquez & Devesa (1996) as *S. pauneroana* (Martinovský) F.M.Vázquez & Devesa. ***S. iranica*** Freitag: ***n* = 22**: Sheidai & Attaei (2005). ***S. juncea*** L.: **2*n* = 44**: Myers (1947) citing an unpubl. count of R.M. Love; Nilsson & Lassen (1971); Vázquez & Devesa (1996) as *S. juncea* var. *cabanasii* F.M.Vázquez & Devesa; Vázquez & Devesa (1996) as *S. juncea* L. var. *juncea*. ***S. kirghisorum*** P.A.Smirn.: **2*n* = 32**: Solntzeva (1967). ***S. krylovii*** Roshev.: **2*n* = 44**: Sokolovskaya & Probatova (1978); Chepinoga & al. (2012); Probatova & al. (2013, 2016b); Chepinoga & Gnutikov (2014); Gnutikov & al. (2017). ***S. lagascae*** Roem. & Schult.: ***n* = 22**: Ferchichi & al. (1994) as *S. lagascae* var. *pubescens* Hack.; **2*n* = 44**: Gould & Soderstrom (1970): Faruqi & al. (1987); Devesa & al. (1991); Vázquez & Devesa (1996) as *S. clausa* subsp. *cazorlensis* F.M.Vázquez & Devesa; Vázquez & Devesa (1996) as *S. clausa* Trab. subsp. *clausa* var. *clausa*; Vázquez & Devesa (1996) as *S. lagascae* var. *australis* Maire; Vázquez & Devesa (1996) as *S. lagascae* var. *lagascae*. ***S. lessinigiana*** Trin. & Rupr.: ***n* = 22**: Sheidai & Attaei (2005); **2*n* = 44**: Avdulov (1931: 130); Petrova (1968); Lungeanu (1975); Chopanov & Yurtsev (1976); Magulaev (1984); Strid & Andersson (1985) as *Stipa cyllenaea* Strid; Ghukasyan (2004). ***S. lingua*** Junge: **2*n* = 44**: Chopanov & Yurtsev (1976). ***S. offneri*** Breistr.: **2*n* = 44**: Vázquez & Devesa (1996). ***S. orientalis*** Trin. ex Ledeb.: ***n* = 10**: Singhal & al. (2014); **2*n* = 36**: Sokolovskaya & Strelkova (1939) but regarded as miscount by Probatova & Sokolovskaya (1980); **2*n* = 44**: Sokolovskaya & Probatova (1978); Agapova & al. (1993) citing an unpubl. count of O.I. Zakharyeva. ***S. pennata*** L.: ***n* = 22**: Duckert-Henriod (1991) as *S. pennata* subsp. *eriocaulis* (Borbas) Martinovský & Skalický; **2*n* = 44**: Avdulov (1931: 130) as *S. joannis* Čelak.; Tarnavschi (1948) as *S. joannis*; Petrova (1968) as *S. lithophila* P.A.Smirn.; Tarnavschi & Lungeanu (1970a,b) as *S. danubialis* Dihoru & Roman; Tarnavschi & Lungeanu (1970a,b) as *S. eriocaulis* Borbás; Prokudin & al. (1977) citing an unpubl. count of O.A. Petrova; Májovský (1978) as *S. eriocaulis* subsp. *austriaca* (Beck) Martinovský; Andreev (1982) as *S. joannis*; Krasnikov (1991); Vázquez & Devesa (1996) as *S. eriocaulis*; Winterfeld & al. (2015). ***S. pulcherrima*** K.Koch: **2*n* = 44**: Avdulov (1931: 130); Prokudin & al. (1977) citing an unpubl. count of O.A. Petrova; Baden (1983) as *S. epilosa* Martinovský; Strid & Andersson (1985) as *S. epilosa*; Moraldo (1986) as *S. crassiculmis* subsp. *picentina* Martinovský; **2*n* = 66**: Kožuharov & Petrova (1991) as *S. epilosa* Martinovský. ***S. sareptana*** A.K.Becker: **2*n* = 44**: Titova (1935). ***S. tirsa*** Steven: **2*n* = 44**: Avdulov (1931: 130) as *S. stenophylla* Czern. ex Trautv.; Prokudin & al. (1977) citing an unpubl. count of O.A. Petrova; Májovský (1978); Winterfeld & al. (2015). ***S. turkestanica*** Hack.: ***n* = 22**: Sheidai & Attaei (2005). ***S. turkestanica*** subsp. ***trichoides*** (P.A.Smirn.) Tzvelev: **2*n* = 40**: Chopanov & Yurtsev (1976) as *S. trichoides* P. A.Smirn. ***S. ucrainica*** P.A.Smirn.: **2*n* = 44**: Avdulov (1931: 130); Prokudin & al. (1977) citing an unpubl. count of O.A. Petrova. ***S. zalesskii*** Wilensky ex Grossh.: **2*n* = 44**: Prokudin & al. (1977) citing an unpubl. count of O.A. Petrova.

Stipellula Röser & Hamasha: **2*n* = 18, <u>28</u>, ca. 34, <u>36</u>**. — ***S. capensis*** (Thunb.) Röser & Hamasha: **2*n* = 18**: Borgen (1970) as *Stipa retorta* Cav.; **2*n* = ca. 34**: Reese (1957) as *S. retorta*; ***n* = 18, 2*n* = 36**: Devesa & al. (1991) as *S. capensis* Thunb.; **2*n* = 36**: Fernandes & Queirós (1969) as *S. capensis*; Dahlgren & al. (1971) as *S. capensis*; Gould & Soderstrom (1970) as *S. capensis*; Queirós (1974) as *S. capensis*; Vázquez & Devesa (1996) as *S. capensis*; Scholz & al. (1998) as *S. capensis*. ***S. parviflora*** (Desf.) Röser & Hamasha: ***n* = 14**: Ferchichi & al. (1994) as *Stipa parviflora* Desf.; **2*n* = 28**: Lorenzo-Andreu (1951, 1953) as *S. parviflora*; Vázquez & Devesa (1996) as *S. parviflora*.

Thorneochloa Romasch., P.M.Peterson & Soreng: **no chromosome counts available**.

Timouria Roshev.: **no chromosome counts available**.

Trikeraia Bor: **no chromosome counts available**.

REFERENCES

Agapova, N.D., Arkharova, K.B., Vakhtina, E.A., Zemskova, E.A., Tarvis, L.V. 1993. *Chisla khromosom tsvetkovykh rasteniy flory SSSR: Moraceae–Zygophyllaceae [Chromosome numbers in flowering plants of the flora of the USSR: Moraceae–Zygophyllaceae]*. St. Petersburg: Nauka, 430 pp.

Andreev, V.N. 1982. Pp. 575–576 in: Löve, Á. (ed.), IOPB chromosome number reports LXXVI. *Taxon* 31(3): 574–598. https://doi.org/10.1002/j.1996-8175.1982.tb03560.x

Avdulov, N.P. 1928. Systematicheskaya karyologiya semeystva Gramineae. Dnyevnik vsesoyuznogo s'ezda botanikov v Leningrade (1928 g.), vol. 1: 65–67. Not seen, cited after Avdulov (1931).

Avdulov N.P. 1931. Kario-sistematicheskoe issledovanie semeistva zlakov. [Karyosystematic study of the family of grasses.] *Trudy Prikl. Bot. Selekts., Suppl.* 44: 1–428.

Baden, C. 1983. Chromosome numbers in some Greek angiosperms. *Willdenowia* 13(2): 335–336. https://www.jstor.org/stable/3995849

Belyaeva, V.A., Siplivinsky, V.N. 1977. Khromosomnye chisla i taksonomiya nekotorykh vidov baikalskoy flory. III. [Chromosome numbers and taxonomy of some species of Baikal flora. III.]. *Bot. Zhurn. (Moscow & Leningrad)* 62(8): 1132–1142.

Borgen, L. 1970. Chromosome numbers of Macaronesian flowering plants. *Nytt Mag. Bot.* 17: 145–161.

Bowden, W.M. 1960. Chromosome numbers and taxonomic notes on northern grasses: III. Twenty-five genera. *Canad. J. Bot.* 38(4): 541–557. https://doi.org/10.1139/b60-049

Bowden, W.M., Senn, H.A. 1962. Chromosome numbers in 28 grass genera from South America. *Canad. J. Bot.* 40(8): 1115–1124. https://doi.org/10.1139/b62-102

Brown, W.V. 1949. A cytological study of cleistogamous *Stipa leucotricha*. *Madroño* 10(4): 97–107. https://www.jstor.org/stable/41422661

Brown, W.V. 1951. Chromosome numbers of some Texas grasses. *Bull. Torrey Bot. Club.* 78(4): 292–299. doi: 10.2307/2481991

CCDB (The Chromosome Counts Database). 2020. Version 1.46. http://ccdb.tau.ac.il/home/. Accessed 21 Apr 2020.

Chepinoga, V.A., Gnutikov, A.A. 2014. P. 1387 in: Marhold, K. (ed.), IAPT/IOPB chromosome data 18. *Taxon* 63(6): 1387–1393. https://doi.org/10.12705/636.37

Chepinoga, V.V., Gnutikov, A.A., Lubogoschinsky, P.I., Isaikina, M.M., Konovalov, A.S. 2012. Pp. 981–982 in: Marhold, K. (ed.), IAPT/IOPB chromosome data 13. *Taxon* 61(4): 889–902. https://doi.org/10.1002/tax.614023

Chopanov, P., Yurtsev, V.N. 1976. Khoromosomnye chisla nekotorykh zlakov Turkmenii. II. [Chromosome numbers of some grasses of Turkmenia. II.] *Bot. Zhurn. (Moscow & Leningrad)* 61(9): 1240–1244.

Cialdella, A.M., Guissani, L.M., Aagesen, L., Zuloaga, F.O., Morrone, O. 2007. A phylogeny of *Piptochaetium* (Poaceae: Pooideae: Stipeae) and related genera based on a combined analysis including *trnL–F, rpl16*, and morphology. *Syst. Bot*. 32: 545–559. https://doi.org/10.1600/036364407782250607

Covas, G. 1945. Número de cromosomas de algunas Gramíneas argentinas. *Revista Argent. Agron.* 12: 315–317.

Covas, G., Bocklet, M. 1945. Número de cromosomas de algunas Gramineae-Stipinae de la flora Argentina. *Revista Argent. Agron.* 12: 261−265.

Curto, M., Henderson, D.M. 1998. A new *Stipa* (Poaceae: Stipeae) from Idaho and Nevada. *Madroño* 45(1): 57–63. https://www.jstor.org/stable/41425241

Dahlgren, P., Karlsson, T., Lassen, P. 1971. Studies on the flora of the Balearic Islands I. Chromosome numbers in Balearic angiosperms. *Bot. Not.* 124: 249–269.

Darlington, C.D., Wylie, A.P. 1956. *Chromosome atlas of flowering plants.* New York: Macmillan.

Davidse, G., Pohl, R.W. 1974. Chromosome numbers, meiotic behavior, and notes on tropical American grasses (Gramineae). *Canad. J. Bot.* 52(2): 317–328. https://doi.org/10.1139/b74-042

Davidse, G., Pohl, R.W. 1978. Chromosome numbers of tropical American grasses (Gramineae) 5. *Ann. Missouri Bot. Gard.* 65(2): 637–649. doi: 10.2307/2398863

Dawson, M.I., Beuzenberg, E.J. 2000. Contributions to a chromosome atlas of the New Zealand flora – 36. Miscellaneous families, *New Zealand J. Bot.* 38(1): 1–23. https://doi.org/10.1080/0028825X.2000.9512671

Dedecca, D.M. 1954. Studies on the California species of *Stipa* (Gramineae). *Madroño* 12(5): 129–139. https://www.jstor.org/stable/41422801

Delay, C. 1947. Recherches sur la structure des noyaux quiescents chez les Phanérogames. *Rev. Cytol. Cytophysiol. Vég.* 9(1–4): 169–222; 10(1–4): 103–229.

Delay, C. 1950. Nombres chromosomiques chez les Phanérogames (I-re liste: 1938 à 1950). *Rev. Cytol. Cytophysiol. Vég.* 12(1–2): 1–368. Not seen, cited after Gould (1966) and Fedorov (1969).

Devesa, J.A., Ruiz, T., Viera, M.C., Tormo, R., Vázquez, F., Carrasco, J.P., Ortega, A., Pastor, J. 1991. Contribución al conocimiento cariológico de las Poaceae en Extremadura (España) III. *Bol. Soc. Brot.* Série 2, 64: 35–74.

de Winter, B. 1965. The South African Stipeae and Aristideae (Gramineae). An anatomical, cytological and taxonomic study. *Bothalia* 8(3):199–404.

Duckert-Henriod, M.M. 1991. Pp. 229–236 in: Kamari, G., Felber, F., Garbari, F. (eds.), Mediterranean chromosome number reports 1. *Fl. Medit* 1: 223–245. http://www.herbmedit.org/flora/01-223.pdf

Edgar, E., Connor, H.E. 2000. *Flora of New Zealand*, vol. 5, *Gramineae.* Lincoln, New Zealand: Manaaki Whenua Press.

Erst, A.S., Mitrenina, E.Yu., Sukhorukov, A.P., Kuzmin, I.V., Gudkova, P.D., Tashev, A.N., Xiang, K., Wang, W. 2019. P. 1126 in: Marhold, K., Kučera, J. (eds.), IAPT chromosome data 30. *Taxon* 68: 1124–1130. https://doi.org/10.1002/tax.12156

Faruqi, S.A., Quraish, H.B., Inamuddin, M. 1987. Studies in Libyan grasses. X. Chromosome number and some interesting features (1). *Ann. Bot. (Rome)* 45(2): 75–102.

Fedorov, A.A. (ed.). 1969. *Khromosomnye chisla tsvetkovykh rasteniiy. [Chromosome numbers of flowering plants]*. Leningrad: Nauka, 927 pp.

Ferchichi, A., Nabli, N.A., Delay, J. 1994. Prospection caryologique de la famille des Poaceae en Tunisie steppique. *Acta Bot. Gallica.* 141(3): 327–341. https://doi.org/10.1080/12538078.1994.1051516

Fernandes, A., Queirós, M. 1969. Contribution à la connaissance cytotaxinomique des Spermatophyta du Portugal. I. Gramineae. *Bol. Soc. Brot.* Série 2, 43: 20–144.

Freitag, H. 1985. The genus *Stipa* (Gramineae) in southwest and south Asia. *Notes Roy. Bot. Gard. Edinburgh* 42: 355–489.

Gallego, M.J., Talavera, S. 1994. Números cromosomáticos de plantas occidentales, 696–707. *Anales Jard. Bot. Madrid* 51(2): 280.

Gervais, C. 1966. Nombres chromosomiques chez quelques graminées alpines. *Bull. Soc. Neuchâtel. Sci. Nat.* 89: 87–100. http://doi.org/10.5169/seals-88960

Ghukasyan, A.G. 2004. Kariologisheskaya izuchennost zlakov (Poaceae) Armenii. [Extent of karyological study of Armenian grasses (Poaceae).] *Fl. Rastitel'nost' Rastitel'nye Resursy Armenii [Flora, vegetation and plant resources of Armenia]* 15: 74–84. http://takhtajania.asj-oa.am/id/eprint/297

Gnutikov, A.A., Myakoshina, Yu.A., Punina, E.O., Rodionov, A.V. 2017. A karyological study of grasses (Poaceae) of Altai. II. *Turczaninowia* 20(2): 16–22. https://doi.org/10.14258/turczaninowia.20.2.2

Gohil, R.N., Koul, K.K. 1986. SOCGI plant chromosome number reports IV, Gramineae (Poaceae). *J. Cytol. Genet.* 21: 155.

González, A.C., Vaio, M., Porro, V., Folle, G., Mazzella, C. 2017. Chromosome numbers, DNA content, morphological data, and nrITS sequence analyses in some species of *Nassella* (Trin.) E. Desv. and related genera (Stipeae, Poaceae). *Brazil. J. Bot.* 40: 341–352. https://doi.org/10.1007/s40415-016-0337-0

Gould, F.W. 1958. Chromosome numbers in southwestern grasses. *Amer. J. Bot.* 45(10): 757–767. https://doi.org/10.1002/j.1537-2197.1958.tb10608.x

Gould, F.W. 1965. Chromosome numbers in some Mexican grasses. *Bol. Soc. Bot. México.* 29: 49–62. https://doi.org/10.17129/botsci.1088

Gould, F. W. 1966. Chromosome numbers of some Mexican grasses. *Canad. J. Bot.* 44(12): 1683–1696. https://doi.org/10.1139/b66-181
Gould, F.W. 1968. Chromosome numbers of Texas grasses. *Canad. J. Bot.* 46(10): 1315–1325. https://doi.org/10.1139/b68-175
Gould, F.W., Soderstrom, T.R. 1970. Pp. 104–104 in: Löve, Á. (ed.), IOPB chromosome number reports. XXV. *Taxon* 19: 102–113. https://doi.org/10.1002/j.1996-8175.1970.tb02985.x
Gusik, M.B. 1973. Sutochnyi ritm i ekologiya tsveteniya i opyleniya zlakov stepei Zabaikalya i Khakassii [Daily rhythm and ecology of flowering and pollination in the grasses of the steppes of Transbaikal and Khakassia regions). Ph.D. thesis abstract, Perm. Not seen, cited after Tzvelev (1976).
Gusik, M.B., Levkovskiy, V.P. 1979. Khromosomnye chisla dikorastushchikh zlakov Zabaikalya i Khakassii. [Chromosome numbers of wild grasses of Transbaikal and Khakassia regions]. Ekologia opylenia. Perm. 4: 26–32. Not seen, cited after Agapova & al. (1993).
Gupta, R.C., Chauhan, H.S., Saggoo, M.I.S., Kaur, N. 2014. Cytomorphology of some grasses (Poaceae) from Lahaul-Spiti (Himachal Pradesh), India. *Biolife* 2(4): 1234–1247.
Hatch, S.L. 1980. Chromosome numbers of some grasses from the southwestern United States and Mexico. *S.W. Naturalist* 25(2): 278–280. doi: 10.2307/3671259
Hatch, S.L., Bearden, D.A. 1983. *Stipa curvifolia* (Poaceae) – studies on a rare taxon. *Sida* 10(2): 184–187. https://www.jstor.org/stable/23909549
Hitchcock, C.L., Spellenberg, R.W. 1968. A new *Oryzopsis* from Idaho. *Brittonia* 20: 162–165. https://doi.org/10.2307/2805618
Johnson, B.L. 1945. Cytotaxonomic studies in *Oryzopsis*. *Bot. Gaz.* 107(1): 1–32. https://www.jstor.org/stable/2472124
Johnson, B.L. 1960. Natural hybrids between *Oryzopsis* and *Stipa*. I. *Oryzopsis hymenoides* × *Stipa speciosa*. *Amer. J. Bot.* 47: 736–742. https://doi.org/10.1002/j.1537-2197.1960.tb07159.x
Johnson, B.L. 1962a. Natural hybrids between *Oryzopsis* and *Stipa*. II. *Oryzopsis hymenoides* × *Stipa nevadensis*. *Amer. J. Bot.* 49: 540–546. https://doi.org/10.1002/j.1537-2197.1962.tb14978.x
Johnson, B.L. 1962b. Amphiploidy and introgression in *Stipa*. *Amer. J. Bot.* 49: 253–262. https://doi.org/10.1002/j.1537-2197.1962.tb14935.x
Johnson, B.L. 1963. Natural hybrids between *Oryzopsis* and *Stipa*. III. *Oryzopsis hymenoides* × *Stipa pinetorum*. *Amer. J. Bot.* 50: 228–234. https://doi.org/10.1002/j.1537-2197.1963.tb12228.x
Johnson, B.L., Rogler, G.A. 1943. A cyto-taxonomic study of an intergeneric hybrid between *Oryzopsis hymenoides* and *Stipa viridula*. *Amer. J. Bot.* 30(1): 49–56. https://doi.org/10.1002/j.1537-2197.1943.tb14731.x
Kaur, H., Gupta, R.C., Kumari, S. 2011a. P. 1789 in: Marhold, K. (ed.), IAPT/IOPB chromosome data 12. *Taxon* 60(6): 1784–1796. https://doi.org/10.1002/tax.606033
Kaur, H., Kumari, S.,, Gupta, R.C. (2011b). New chromosome numbers reported in grasses from Himachal Pradesh (India). *Chromosome Bot.* 6(1): 13–16. doi:10.3199/iscb.6.13
Kerguélen, M. 1975. Les Gramineae (Poaceae) de la flore française. Essai de mise au point taxonomique et nomenclaturale. *Lejeunia, nouv. sér.* 75: 1–343.
Korobkov, A.A., Kotseruba, V.V., Probatova, N.S., Shatokhina, A.V., Rudyka, E.G. 2013. pp. 1075–1077 in: Marhold, K. (ed.), IAPT/IOPB chromosome data 15. *Taxon* 62(5): 1073–1083. https://doi.org/10.12705/625.16
Kožuharov, S., Kuzmanov, B. 1968. Cytotaxonomic studies on Bulgarian Gramineae. *C. R. Acad. Bulg. Sci.* 21: 269–272.
Kožuharov, S.I., Petrova, A.V. 1991. Chromosome numbers of Bulgarian angiosperms. *Fitologija* 39: 72–77.
Krogulevich, R.E. 1971. Rol poliploidii v genezise vysokogornoy flory Stanovogo nagorya. Pp. 115–214 in: *Ekologiya flory Zabaikalya*. Irkutsk. Not seen, cited after Probatova & Sokolovskaya (1980) and Murín & al. (1984).
Krogulevich, R.E. 1972. Vliyanie botaniko-geograficheskikh faktorov na poliploidiyu [Effect of botanical-geographic factors on polyploidy]. Pp: 12–13. in: *Nauchnye chteniya pamyati M.G. Popova*. lrkutsk. Not seen, cited after Tzvelev (1976).

Krasnikov, A.A. 1991. Chisla khromosom nekotorykh vidov sosudistykh rasteniy Novosibirskoy oblasti. [Chromosome numbers in some species of vascular plants from Novosibirsk region.] *Bot. Zhurn. (Moscow & Leningrad)* 76(3): 476–479.
Kumar, R., Kumari, V., Singhal, V.K. 2018. Pp. 1238–1239 in: Marhold, K., Kučera, J. (eds.), IAPT chromosome data 28. *Taxon* 67(6): 1235–1245. https://doi.org/10.12705/676.39
Kumar, V., Subramaniam, B. 1987. *Chromosome atlas of flowering plants of the Indian subcontinent*, vol. 2, *Monocotyledons and bibliography.* Calcutta: Botanical Survey of India. Pp. 465–1095.
Kumari, V., Kumar, R., Singhal, V.K. 2019. P. 1379 in: Marhold, K., Kučera, J. (eds.), IAPT chromosome data 31. *Taxon* 68(6): 1374–1380. https://doi.org/10.1002/tax.12176
Kuzmanov, B. 1993. Chromosome numbers of Bulgarian angiosperms: an introduction to a chromosome atlas of the Bulgarian flora. *Fl. Medit.* 3: 19–163.
Labadie, J.P. 1979a. Pp. 628–629 in: Löve, Á. (ed.), IOPB chromosome number reports. LXV. *Taxon* 28: 627–637.
Labadie, J. 1979b. Étude caryosystematique de quelques espèces de la flore d’Algérie. *Naturalia monspeliensia, sér. Bot.* 32: 1–11.
de Litardière, R. 1950. Nombres chromosomiques de diverses graminées. *Bol. Soc. Brot.* sér. 2, 24: 79–87.
Lorenzo-Andreu, A. 1951. Cromosomas de plantas de la estepa de Aragón. III. *An. Estac. Exp. Aula Dei* 2(2): 195–203. http://hdl.handle.net/10261/38176
Lorenzo-Andreu, A. 1953. Notas sobre cariología. Pp. 87–91 in: Servicio del Esparto (ed.), *Estudios y experiencias sobre el esparto, 2.ª parte.* Madrid: Ministerios de Industria, Comercio y Agricultura. Not seen, cited after Vazques & Devesa (1991)
Löve, Á., Kjellqvist, E. 1973. Cytotaxonomy of Spanish plants. II. Moncotyledons. *Lagascalia* 3(2): 147–182.
Löve, Á., Löve, D. 1961. Chromosome numbers of central and northwest European plant species. *Opera Bot.* 5: 1–581. Not seen, cited after Fedorov (1969).
Löve, Á., Löve, D. 1974. *Cytotaxonomical atlas of the Slovenian flora.* Lehre: Cramer.
Löve, Á., Löve, D. 1981. Pp. 509–511 in: Löve, Á. (ed.), Chromosome number reports. LXXI. *Taxon* 30(2): 507–516. https://doi.org/10.1002/j.1996-8175.1981.tb00780.x
Love, R.M. 1946. Interspecific hybridization in *Stipa* L. I. Natural hybrids. *Amer. Naturalist.* 80 (no. 788): 189–192. https://doi.org/10.1086/281376
Love, R.M. 1954. Interspecific hybridization in *Stipa* II. Hybrids of *S. cernua*, *S. lepida*, and *S. pulchra*. *Amer. J. Bot.* 41: 107–110. https://doi.org/10.1002/j.1537-2197.1954.tb14311.x
Lungeanu, I. 1975. Pp. 503–504 in: Löve, Á. (ed.), IOPB chromosome number reports XLIX. *Taxon* 24(4): 501–516. https://doi.org/10.1002/j.1996-8175.1975.tb00341.x
Lungeanu, I. 1980. Pp. 164–165 in: Löve, Á. (ed.), IOPB chromosome number reports. LXVI. *Taxon* 29(1): 163–169. https://doi.org/10.1002/j.1996-8175.1980.tb00607.x
Luque, T., Diaz Lifante, Z. 1991. Chromosome numbers of plants collected during Iter Mediterraneum I in the SE of Spain. *Bocconea* 1: 303–364. http://www.herbmedit.org/bocconea/1-303.pdf
Magulaev, A.Yu. 1984. Tzitotaksonomicheskoe izuchenie nekotorykh tzvetkovykh rasteniy Severnogo Kavkasa. [Cytotaxonomic study in some flowering plants of the North Caucasus.] *Bot. Zhurn. (Moscow & Leningrad)* 69(4): 511–517.
Májovský, J. (ed.). 1974. Index of chromosome numbers of Slovakian flora. (Part 3). *Acta Fac. Rerum Nat. Univ. Comen., Bot.* 22: 1–20.
Májovský, J. (ed.). 1978. Index of chromosome numbers of Slovakian flora (Part 6). *Acta Fac. Rerum Nat. Univ. Comen., Bot.* 26: 1–42.
Marhold, K., Mártonfi, P., Mereďa, P., Mráz, P. (eds.). 2007. *Chromosome number survey of the ferns and flowering plants of Slovakia.* Bratislava: Veda.
Mehra, P.N., Sharma, M.L. 1975a. Cytological studies in some central and eastern Himalayan grasses. IV. The Arundinelleae, Eragrosteae, Isachneae, Chlorideae, Sporoboleae, Meliceae, Stipeae, Arundineae and Garnotieae. *Cytologia* 40: 453–462. https://doi.org/10.1508/cytologia.40.453
Mehra, P.N., Sharma, M.L. 1975b. Pp. 501–502 in: Löve, Á. (ed.), IOPB chromosome number reports XLIX. *Taxon* 24(4): 501–516. https://doi.org/10.1002/j.1996-8175.1975.tb00341.x
Mehra, P.N., Sharma, M.L. 1977. Cytological studies on some grasses of Kashmir. *Cytologia* 42: 111–123. https://doi.org/10.1508/cytologia.42.111

Mehra, P.N., Sunder, S. 1969. Cytological studies in the north Indian grasses. Part II. *Res. Bull. Panjab Univ. Sci.* 20: 503–539.
Měsíček, J., Javůrcová-Jarolímová, V. 1992. List of chromosome numbers of the Czech vascular plants. Praha: Academia. 144 pp.
Měsíček, J., Soják, J. 1972. Chromosome studies in Mongolian plants. *Preslia* 44: 334–358.
Moinuddin, M., Vahidy, A.A., Ali, S.I. 1994. Chromosome counts in Arundinoideae, Chloridoideae, and Pooideae (Poaceae) from Pakistan. *Ann. Missouri Bot. Gard.* 81(4): 784–791. doi: 10.2307/2399923
Moraldo, B. 1986. Il genere *Stipa* L. (Gramineae) in Italia. *Webbia* 40(2): 203–278. https://doi.org/10.1080/00837792.1986.10670388
Murín, A., Háberová, I., Žamsran, C. 1984. Further karyological studies of the Mongolian flora. *Folia Geobot. Phytotax.*19: 28–39. https://www.jstor.org/stable/4180474
Murray, B.G., de Lange, P.J., Ferguson, A.R. 2005. Nuclear DNA variation, chromosome numbers and polyploidy in the endemic and indigenous grass flora of New Zealand. *Ann. Bot. (Oxford)* 96: 1293–1305. https://doi.org/10.1093/aob/mci281
Myers, W.M. 1947. Cytology and genetics of forage grasses. *Bot. Rev. (Lancaster)* 13(6–7): 319–422. https://www.jstor.org/stable/4353363 and https://www.jstor.org/stable/4353364
Nielsen, E.L. 1939. Grass studies III. Additional somatic chromosome complements. *Amer. J. Bot.* 26(6): 369–372. https://doi.org/10.1002/j.1537-2197.1939.tb09289.x
Nilsson, Ö., Lassen, P. 1971. Chromosome numbers of vascular plants from Austria, Mallorca and Yugoslavia. *Bot. Not.* 124: 270–276.
Ono, H., Tateoka, T. 1953. Karyotaxonomy in Poaceae I. Chromosomes and taxonomic relations in some Japanese grasses. *Bot. Mag. (Tokyo)* 66: 18–27.
Parodi, L.R. 1946. *Gramíneas bonarienses. Clave para la determinación de los géneros y enumeración de las especies*, 4th ed. Buenos Aires: Centro Estudiantes de Agronomía.
Petrova, O.A. 1968. Khromosomnyi sostav nekotorykh zlakov flory Ukrainy v svyasi s usloviami ikh proizrastaniya. [Chromosomal composition of some Ukrainian grasses according to their growing conditions.] Pp. 37–39 in: Nikitin, V.N., (ed.), *Biologicheskaya nauka v universitetakh I pedagogicheskikh institutakh Ukrainy za 50 let. [Biological science in universities and pedagogical institutes of Ukraine for 50 years].* Charkov: Charkov University. Not seen, cited after Prokudin & al. (1977) and Agapova & al. (1993).
Podlech, D., Dieterle, A. 1969. Chromosomenstudien an afghanischen Pflanzen. *Candollea* 24: 185–243.
Pohl, R.W. 1954.The allopolyploid *Stipa latiglumis*. *Madroño* 12(5): 145–150. https://www.jstor.org/stable/41422803
Pohl, R.W., Davidse, G. 1971. Chromosome numbers of Costa Rican grasses. *Brittonia* 23(3): 293–324. doi: 10.2307/2805632
Probatova, N.S., Seledets, V. 2008. Pp. 553–558 in: Marhold, K. (ed.), IAPT/IOPB chromosome data 5. *Taxon* 57(2): 553–562. https://doi.org/10.2307/25066021
Probatova, N.S., Sokolovskaya, A.P. 1980. K kariotaksonomicheskomu isucheniyu zlakov gornogo Ataya. [To the karyotaxonomic study of the grasses of Mountain Altai.] *Bot. Zhurn. (Moscow & Leningrad)* 65(4): 509–520.
Probatova, N.S., Rudyka, E.G., Pavlova, N.S., Verkholat, V.P., Nechaev, V.A. 2006. Chisla khromosom vidov rasteniy iz Primorskogo kraya, Priamurya i Magadanskoy oblasti. [Chromosome numbers of plants of the Primorsky Territory, the Amur River basin and Magadan region.] *Bot. Zhurn. (Moscow & Leningrad)* 91(3): 491–509.
Probatova, N.S., Rudyka, E.G., Seledets, V.P., Nechaev, V.A. 2008. Pp. 1268–1271 in: Marhold, K. (ed.), 2008. IAPT/IOPB chromosome data 6. *Taxon* 57(4): 1267–1273. https://doi.org/10.1002/tax.574017
Probatova, N.S., Sedelets, V.P., Rudyka, E.G., Gnutikov, A.A., Kozhevnikova, Z.V., Barkalov, V.Yu. 2009. Pp.1284–1288 in: Marhold, K. (ed.), IAPT/IOPB chromosome data 8. *Taxon* 58(4): 1281–1289. https://doi.org/10.1002/tax.584017
Probatova, N.S., Kazanovsky, S.G., Rudyka, E.G., Barkalov, V.Yu., Seledets, V.P., Nechaev, V.A. 2011. Pp. 1790–1794 in: Marhold, K. (ed.), IAPT/IOPB chromosome data 12. *Taxon* 60(6): 1784–1796. https://doi.org/10.1002/tax.606033

Probatova, N.S., Kazanovsky, S.G., Shatokhina, A.V. Rudyka, E.G., Verkhozina, A.V., Krivenko, D.A. 2012. Pp. 1342–1344 in: Marhold, K. (ed.), IAPT/IOPB chromosome data 14. *Taxon* 61(6): 1336–1345. https://doi.org/10.1002/tax.616027

Probatova, N.S., Kazanovsky, S.G., Rudyka, E.G., Gnutikov, A.A., Verkhozina, A.V. 2013. Pp. 1080–1081 in: Marhold, K. (ed.), IAPT/IOPB chromosome data 15. *Taxon* 62(5): 1073–1083. https://doi.org/10.12705/625.16

Probatova, N.S., Kazanovsky, S.G., Barkalov, V.Yu, Rudyka, E.G., Shatokhina, A.V. 2015. Pp. 1348–1349 in: Marhold, K. (ed.), IAPT/IOPB chromosome data 20. *Taxon* 64(6): 1344–1350. https://doi.org/10.12705/646.42

Probatova, N.S., Kazanovsky, S.G., Barkalov, V.Yu., Nechaev, V.A. 2016a. Pp. 1202–1204 in: Marhold, K., Kučera, J. (eds.), IAPT/IOPB chromosome data 22. *Taxon* Vol. 65(5): 1200–1207. https://doi.org/10.12705/655.40

Probatova, N.S., Krivenko, D.A., Ebel, A.L. 2016b. Pp. 1204–1205 in: Marhold, K., Kučera, J. (eds.), IAPT/IOPB chromosome data 22. *Taxon* Vol. 65(5): 1200–1207. https://doi.org/10.12705/655.40

Prokudin, Yu.N, Vovk, A.G, Petrova, O.A, Ermolenko, E.D., Vernichenko, Yu.V. (ed.). 1977. *Zlaki Ukrainy* [*Grasses of Ukraine*]. Kiev: Naukova Dumka.

Queirós, M. 1973. Contribuição para o conhecimento citotaxonómico das Spermatophyta de Portugal. I. Gramineae, supl. 1. *Bol. Soc. Brot. Ser. 2*, 47: 77–103.

Queirós, M. 1974. Contribuição para o conhecimento citotaxonómico das Spermatophyta de Portugal. I. Gramineae, supl. 2. *Bol. Soc. Brot. Ser. 2*, 48: 81–98.

Reeder, J.R. 1967. Notes on Mexican grasses VI. Miscellaneous chromosome numbers. *Bull. Torrey Bot. Club* 94(1): 1–17. https://www.jstor.org/stable/2483595

Reeder, J.R. 1968. Notes on Mexican Grasses VIII. Miscellaneous chromosome numbers – 2. *Bull. Torrey Bot. Club* 95(1): 69–86. https://www.jstor.org/stable/2483808

Reeder, J.R. 1977. Chromosome numbers in western grasses. *Amer. J. Bot* 64(1): 102–110. https://doi.org/10.1002/j.1537-2197.1977.tb07611.x

Reeder, J.R. 1984. Pp. 132–133 in: Löve, Á. (ed.), Chromosome number reports LXXXII. *Taxon* 33(1): 126–134. https://doi.org/10.1002/j.1996-8175.1984.tb02474.x

Reeder, J.R., Reeder, C.G. 1968. *Parodiella*, a new genus of grasses from the high Andes. *Bol. Soc. Argent. Bot.* 12: 268–283.

Reese, G. 1957. Über die Polyploidiespektren in der nordsaharischen Wüstenflora. *Flora* 144(4): 598–634.

Romaschenko, K., Peterson, P.M., Soreng, R.J., Garcia-Jacas, N., Futorna, O., Susanna, A. 2012. Systematics and evolution of the needle grasses (Poaceae: Pooideae: Stipeae) based on analysis of multiple chloroplast loci, ITS, and lemma micromorphology. *Taxon* 61(1): 18–44. https://doi.org/10.1002/tax.611002

Rudyka, E.G. 1990. Chisla khromosom sosudistykh rasteniy iz raslichnykh regionov SSSR. [Chromosome numbers of vascular plants from the various regions of the USSR.] *Bot. Zhurn. (Moscow & Leningrad)* 75(12): 1783–1786.

Saura, F. 1943. Cariología de Gramíneas. Géneros *Paspalum*, *Stipa*, *Poa*, *Andropogon* y *Phalaris*. *Rev. Fac. Agron. Vet. (Buenos Aires)* 10: 344–353. Not seen, cited after Myers (1947) and Bowden & Senn (1962).

Saura, F. 1948. Cariología de Gramíneas en Argentina. *Rev. Fac. Agron. Vet. (Buenos Aires)* 12: 56–67. Not seen, cited after Bowden & Senn (1962).

Scholz, H., Oberprieler, C., Vogt, R. 1998. Chromosome numbers of North African phanerogams. VII. Some notes on North African Gramineae. *Lagascalia* 20: 265–275.

Sharma, M.L., Sharma, K. 1978. Pp. 390–391 in: Löve, Á. (ed.), IOPB chromosome number reports LXI. *Taxon* 27(4): 375–392. https://doi.org/10.1002/j.1996-8175.1978.tb04261.x

Sharma, M.L., Sharma, K. 1979. Cytological studies in the north Indian grasses. *Cytologia* 44: 861–872.

Shechter, Y., Johnson, B.L. 1966. A new species of *Oryzopsis* (Gramineae) from Wyoming. *Brittonia* 18(4): 342–347. doi: 10.2307/2805150

Sheidai, M., Attaei, S. 2005. Meiotic studies of some *Stipa* (Poaceae) species and populations in Iran. *Cytologia* 70(1): 23–31.

Singhal, V.K., Kumari, V., Kumar, P.V. 2014. Cytomorphological diversity in some selected members of Poaceae from Parvati Valley in Kullu district of Himachal Pradesh, India. *Pl. Syst. Evol.* 300(6): 1385-1408. doi: 10.1007/s00606-013-0969-5
Skalińska, M., Pogan, E., Jankun, A. 1968. Dalsze badania nad kariologią flory polskiej Cz. VII. [Further studies in chromosome numbers of Polish angiosperms. Seventh contribution.] *Acta Biol. Cracov., Ser. Bot.* 11(2): 199–224.
Sokolovskaya, A.P., Probatova, N.S. 1977. Kariologicheskoe issledovanie zlakov (Poaceae) yuzhnoy chasti Sovetskogo Dalnego Vostoka. [Karyological investigation of grasses (Poaceae) in southern part of the Soviet Far East.] *Bot. Zhurn. (Moscow & Leningrad)* 62(8): 1143–1153.
Sokolovskaya, A.P., Probatova, N.S. 1978. Khromosomnye chisla nekotorykh zlakov (Poaceae) flory SSSR. II. [Chromosome numbers of some grasses (Poaceae) of the U.S.S.R. flora. II.] *Bot. Zhurn. (Moscow & Leningrad)* 63(9): 1247–1257.
Sokolovskaya, A.P., Strelkova, O.S. 1939. Geograficheskoye raspredelenie poliploidov. I. Issledovanie rastitelnosti Pamira. *Uch. zap. Leningr. gor. univ*., ser. biol. 9: 35; *Tr. Petergofsk. boil. inst.* 17: 42–63. Not seen, cited after Fedorov (1969).
Sokolovskaya, A.P., Strelkova, O.S. 1948. Geograficheskoye raspredelenie poliploidov. II. Issledovanie flory Altaya. *Uch. zap. Ped. Inst. im. Gertzena*, Leningrad 66: 179–193. Not seen, cited after Fedorov (1969).
Solntzeva, M.P. 1967. Mikrosporogenez i razvitie muzhskogo gametofita u kovyley. [The microsporogenesis and the development of the male gamethophyte in feather-grasses.] *Bot. Zhurn. (Moscow & Leningrad)* 52(12): 1757–1772.
Spellenberg, R. 1968. Notes on *Oryzopsis hendersonii* (Gramineae). *Madroño* 19(7) 283–286. https://www.jstor.org/stable/41423321
Spellenberg, R. 1970. Pp. 112–113 in: Löve, A. (ed.), IOPB chromosome number reports. XXV. *Taxon* 19(1): 102–113. https://doi.org/10.1002/j.1996-8175.1970.tb02985.x
Stebbins, G.L., Love, R.M. 1941. A cytological study of California forage grasses. *Amer. J. Bot.* 28(5): 371−382. https://doi.org/10.1002/j.1537-2197.1941.tb07983.x
Stepanov, N.V. 1994. Chisla khromosom nekotorykh taksonov vysshikh rasteniy flory Krasnoyarskogo kraya. [Chromosome numbers of some higher plants taxa of the flora of Krasnoyarsk region.] *Bot. Zhurn. (Moscow & Leningrad)* 79(2): 135–139.
Strid, A. 1983. Pp. 139–140 in: Löve, A. (ed.), IOPB chromosome number reports LXXVIII. *Taxon* 32(1): 138–141. https://doi.org/10.1002/j.1996-8175.1983.tb02410.x
Strid, A. 1987. Chromosome numbers of Turkish mountain plants. *Notes Roy. Bot. Gard. Edinburgh* 44(2): 351–356.
Strid, A., Andersson, I.A. 1985. Chromosome numbers of Greek mountain plants. An annotated list of 115 species. *Bot. Jahrb. Syst.* 107(1–4): 203–228.
Strobl, W., Wittmann, H. 1985. Beiträge zur Kenntnis von Verbreitung, Soziologie und Karyologie von *Achnatherum calamagrostis* (L.) P.B. im Bundesland Salzburg (Österreich). *Ber. Bayer. Bot. Ges.* 56: 95–102.
Tarnavschi, I.T. 1948. Die Chromosomenzahlen der Anthophyten-Flora von Rumänien mit einem Ausblick auf das Polyploidie-Problem. *Bot. Mus. Bot. Univ. Cluj* 28, suppl. 1–130. [Buletinul Grădinii Botanice şi al Muzeului Botanic dela Universitatea din Cluj]. Not seen, cited after Fedorov (1969) and Vázquez & Devesa (1996).
Tarnavschi, I.T., Lungeanu, I. 1970a. Pp. 609–610 in: Löve, A. (ed.), IOPB chromosome number reports XXVIII. *Taxon* 19: 608–610. https://doi.org/10.1002/j.1996-8175.1970.tb03062.x
Tarnavschi, I.T., Lungeanu, I. 1970b. Chromosomenzahlen von einigen in Rumänien wildwachsenden Anthophyten. *Rev. Roum. Biol., Bot.* 15(6): 381–383.
Tateoka, T. 1955. Karyotaxonomy in Poaceae III. Further studies of somatic chromosomes. *Cytologia* 20(4): 296–306. https://doi.org/10.1508/cytologia.20.296
Tateoka, T. 1957. Notes on some grasses VI. *Bot. Mag. (Tokyo)* 70: 119–125. https://doi.org/10.15281/jplantres1887.70.119
Tateoka, T. 1962. A cytological study of some Mexican grasses. *Bull. Torrey Bot. Club.* 89(2): 77–82. doi: 10.2307/2482528
Tateoka, T. 1967. P. 561–564 in: Löve, A. (ed.), IOPB chromosome number reports. XIV. *Taxon* 16(6): 552–571. https://doi.org/10.1002/j.1996-8175.1967.tb02139.x

Tateoka, T. 1986. Chromosome numbers in the tribe Stipeae (Poaceae) in Japan. *Bull. Natl. Sci. Mus. Tokyo*, B, 12: 151–154.

Titova, N.N. 1935. Poiski rastitelnoy drosofily. [Searches for plant fruit flies.] Sov. botanika 2: 61–62. Not seen, cited after Fedorov (1969).

Tzvelev, N.N. 1976. *Zlaki SSSR*. Leningrad: Nauka.

Vázquez, F.M., Devesa, J.A. 1996. Revisión del género *Stipa* L. y *Nassella* Desv. (Poaceae) en la Península Ibérica e Islas Baleares. *Acta Bot. Malac.* 21: 125–189.

Valencia, J. I., Costas, M. 1968. Estudios citotaxonómicos sobre *Piptochaetium* (Gramineae). *Bol. Soc. Argent. Bot.* 12: 168–179.

Verlaque, R., Reynaud, C., Aboucaya, A. 1997. Pp. 240–246 in: Kamari, G., Felber, F., Garbari, F. (eds.), Mediterranean chromosome number reports 7. *Fl. Medit* 7: 197–275. http://www.herbmedit.org/flora/7-197.pdf

Winterfeld, G. 2006. Molekular-cytogenetische Untersuchungen an Hafergräsern (Aveneae) und anderen Poaceae. *Stapfia* 86: 1–170.

Winterfeld, G., Schneider, J., Becher, H., Dickie, J., Röser, M. 2015. Karyosystematics of the Australasian stipoid grass *Austrostipa* and related genera: chromosome sizes, ploidy, chromosome base numbers, and phylogeny. *Austral. Syst. Bot.* 28: 145–159. https://doi.org/10.1071/SB14029

Zhang, Z.S., Li, L.L., Chen, W.L. 2017. *Ptilagrostis contracta* (Stipeae, Poaceae), a new species endemic to Qinghai-Tibet Plateau. *PloS ONE* 12(1): e0166603. https://doi.org/10.1371/journal.pone.0166603
